# Rules of Engagement for Components of Membrane Protein Biogenesis at the Human Endoplasmic Reticulum

**DOI:** 10.3390/ijms26188823

**Published:** 2025-09-10

**Authors:** Richard Zimmermann

**Affiliations:** Medical Biochemistry and Molecular Biology, Saarland University, 66421 Homburg, Germany; richard.zimmermann@uks.eu

**Keywords:** endoplasmic reticulum (ER), membrane proteins, targeting pathways, membrane protein insertases, N-terminal methionine excision, N-terminal acetylation, siRNA-mediated component depletion, label-free quantitative mass spectrometry, differential protein abundance analysis, identification and characterization of clients

## Abstract

In human cells, the biogenesis of membrane proteins, which account for one quarter of polypeptides and sixty percent of human drug targets, is initiated at the membrane of the endoplasmic reticulum (ER). This process involves N-terminal signal peptides or transmembrane helices in the membrane protein precursors. Over one hundred proteins enable membrane-targeting and -insertion of the precursors as well as their folding and covalent modifications. Four targeting pathways to the Sec61 channel in the ER membrane with their effectors and three cooperating or independent membrane protein–insertases have been identified. We combined knock-down of individual components of these pathways and insertases in HeLa cells with label-free quantitative mass spectrometric analysis of the proteomes. Differential protein abundance analysis in comparison to control cells was employed to identify clients of components involved in the targeting or membrane insertion of precursors. Alternatively, knock-out cells or relevant patient fibroblasts were employed. The features of the client polypeptides were characterized to identify the client types of the different components and, ideally, their rules of engagement. In this review/article-hybrid, the focus is on global lessons from and limitations of the proteomic approach in answering the cell biological question, as well as on new aspects, such as N-terminal acetylation of membrane protein precursors.

## 1. Introduction

**Figure 1:**
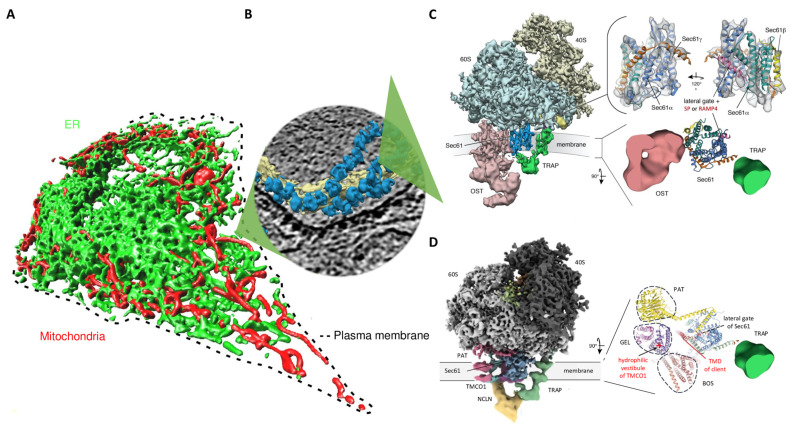
The human endoplasmic reticulum (ER) forms a vast membrane network in nucleated cells and plays a central role in membrane protein biogenesis. (**A**) A 3D reconstruction after live cell fluorescence imaging with ER-resident green fluorescent protein and mitochondrial red fluorescent protein. (**B**,**C**) 3D reconstruction of the native ribosome-translocon complex in the human ER membrane after cryoelectron tomography. In human cells, the heterotrimeric Sec61 complex forms various supercomplexes, e.g., the most abundant one comprising TRAP and OST-A, termed OSTA translocon. Briefly, insertion of precursors with signal peptides (SPs) or transmembrane helices (TMHs) into the Sec61 channel and the concomitant gating of the Sec61 channel to the open conformation can occur spontaneously or involve client-specific auxiliary components of the Sec61 channel, such as TRAP or the Sec62/Sec63 complex. The fully open state of the Sec61 channel allows the translocation of hydrophilic domains of MPs into the ER lumen (via the aqueous channel pore) and the insertion of transmembrane domains (TMDs) into the ER membrane (via the lateral gate). (**D**) Alternatively, the TRAP translocon can form the multipass-TRAP translocon together with the PAT-GEL-BOS supercomplex and allow membrane insertion of TMDs (via the hydrophilic vestibule of TMCO1). In this multipass-TRAP translocon, however, TRAPα and BOS subunit TMEM147 compete for binding to Sec61α and the ribosome [44,45]. Furthermore, binding of BOS and OST-A to Sec61 is mutually exclusive. The Figure was adapted from references [46,47]; panel D was inspired by references [32,44,45]. Abbreviations such as 40S, small ribosomal subunit (shown in yellow); 60S, large ribosomal subunit (shown in blue); BOS, back of Sec61 complex; GEL, GET- and EMC-like, comprising an Oxa1 superfamily MP insertase; OST, Oligosaccharyl transferase; PAT, PAT 10 comprising complex; RAMP4, ribosome-associated MP 4; TRAP, Translocon-associated protein are summarized at the end of the manuscript.

All human cells are surrounded by the plasma membrane and contain the cytosol as the main site of protein synthesis. Nucleated human cells comprise additional membranes and aqueous compartments, which in turn are surrounded by at least one membrane and are referred to as cell organelles, such as mitochondria and the endoplasmic reticulum (ER) (Figure 1A). With the exception of some mitochondrial proteins, all proteins are synthesized on cytosolic 80S ribosomes and, unless their site of action is in the cytosol, must either be inserted into a membrane or transported across a membrane to reach their site of action. With regard to protein biogenesis, questions regarding the mechanisms of targeting and subsequent membrane insertion or transport had to be answered for each non-cytosolic protein. The ER occupies a central position among the cell organelles (Figure 1B). This is where, as discovered by G. Palade in the 1950s, not only the biogenesis of secretory proteins begins, but also the biogenesis of about a third of the respective cellular proteome, i.e., of around 10,000 different soluble and membrane proteins (MPs) [1,2]. Thus, during their biogenesis, proteins of the membranes and the aqueous lumen of the ER, ERGIC, Golgi, endosomes, lysosomes, and secretory vesicles as well as proteins of the nuclear envelope, peroxisomes, lipid droplets, and mitochondria, are first directed to the ER membrane and, subsequently, incorporated into or transported across this membrane. Next, the newly synthesized proteins, with the exception of ER-resident proteins, reach their respective site of action in organelles of endo- and exocytosis or on the cell surface (i.e., in the plasma membrane or in the extracellular space) by vesicular transport, are pinched off as components of newly formed peroxisomes or lipid droplets, or are passed on to the mitochondria via the ER-SURF pathway [3,4,5,6,7,8,9,10].

**Figure 2:**
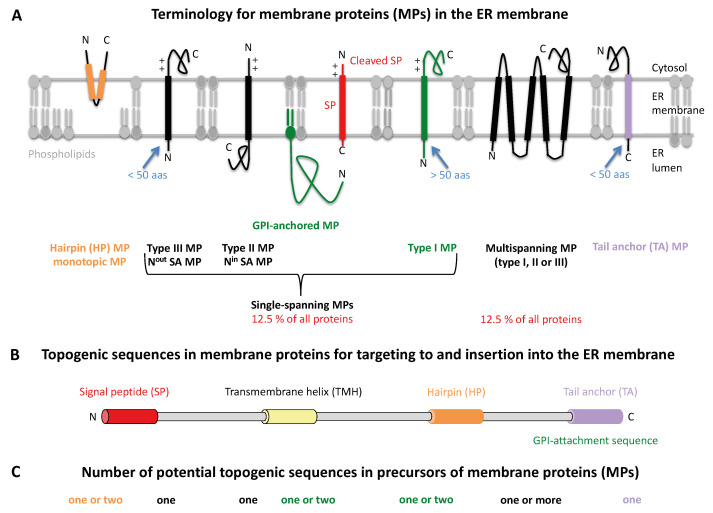
Terminology for membrane proteins in the ER membrane and their topogenic sequences for targeting to and insertion into the ER membrane. (**A**–**C**) The cartoons depict (i) the seven classes of ER MPs with their terminology in bold face in (**A**) and abundance in human cells (in red), (ii) various types of topogenic sequences, such as SP, TMH and GPI-attachment sequence with their typical distributions over a polypeptide chain in (**B**), and (iii) the number of potential topogenic sequences in the seven classes of ER MPs that are shown in (**A**,**C**). (**A**) Cleavable SPs facilitate ER membrane insertion of single-spanning MPs (also termed single-pass MPs, in green) as well as multispanning type I MPs (also termed multipass MPs). TMHs mediate ER membrane insertion of hairpin-, single-spanning type II or III, TA, and multispanning MPs of type II or III (by definition, the shown multispanning MP is of type I or III). The latter MPs depend on more or less amino-terminal TMHs that serve as SP equivalents. (**C**) Precursors of single-spanning type II or III and HP, as well as TA MPs, contain only one topogenic sequence. In precursors of multispanning type I, II, or III and HP, as well as GPI-anchored MPs, the additional TMDs or the GPI-attachment sequence may serve as alternative or additional topogenic sequences. Following ER import and simultaneous cleavage of the SP as well as the C-terminal GPI-attachment sequence, GPI-MPs become membrane-anchored via carboxy-terminal GPI-attachment. (**A**) Size of the ER lumenal domain of MPs matters (given as allowed number of amino acid residues in blue) and positively charged amino acid residues (+) play an important role in the orientation of SPs and MPs in the ER membrane, where the orientation follows the positive inside rule [16,26,27,28,29,30]. The Figure was adapted from references [46,47]. Abbreviations: aas, amino acid residues; C, carboxy-terminus; GPI, glycosylphosphatidylinositol; N, amino-terminus; SA, signal anchor.

One of the key observations by G. Palade was that ribosomes are bound to the ER membrane and are involved in the synthesis of secretory proteins (Figure 1) [1,2]. The establishment of the first cell-free system for studying ER protein import by G. Blobel in the 1970s led to the so-called signal hypothesis, which described ER protein import with its first features [11,12]. The signal hypothesis states that N-terminal signal peptides (SPs) are present in the N-termini of precursors of secretory proteins and ensure the targeting of these nascent polypeptide chains, together with the translating ribosomes, to the ER membrane and, thus, their ER import [3,13,14,15,16,17,18]. Accordingly, the mechanism was defined as cotranslational transport, which involves the proteolytic removal of the respective SP by a signal peptidase complex (SPC) and often various N-gylcosylation events by an oligosaccharyl transferase (OST) (Table S1) [19,20,21,22]. In contrast, the fully synthesized precursors of nuclear-encoded mitochondrial proteins were found to accumulate in the cytosol before being imported into the mitochondria, which was defined as posttranslational protein transport [23,24,25]. Later, it was shown that the biogenesis of various MPs at the ER shares several aspects with the biogenesis of secretory proteins, with the exception that not all MP precursors contain cleavable N-terminal SPs, but rely on physically and functionally similar peptides that represent the only one or one of several transmembrane domains (TMDs) in the mature MPs. The most N-terminal of these are referred to here as transmembrane helices (TMHs) and can be located anywhere in the nascent precursor polypeptide [26,27,28,29,30,31,32]. Several types of MPs are present in the ER membrane and referred to as type I, II, or III MPs, single-spanning or multispanning MPs, and tail anchor- or hairpin-MPs, with the terminology reflecting the number and position of their TMDs (Figure 2A–C) [26,27,28,29,30,31,32,33,34,35,36,37,38,39,40,41,42,43]. Apparently, these different types of MPs also differ in their demands on the machinery for ER targeting and membrane insertion, which will be described next.

**Figure 3:**
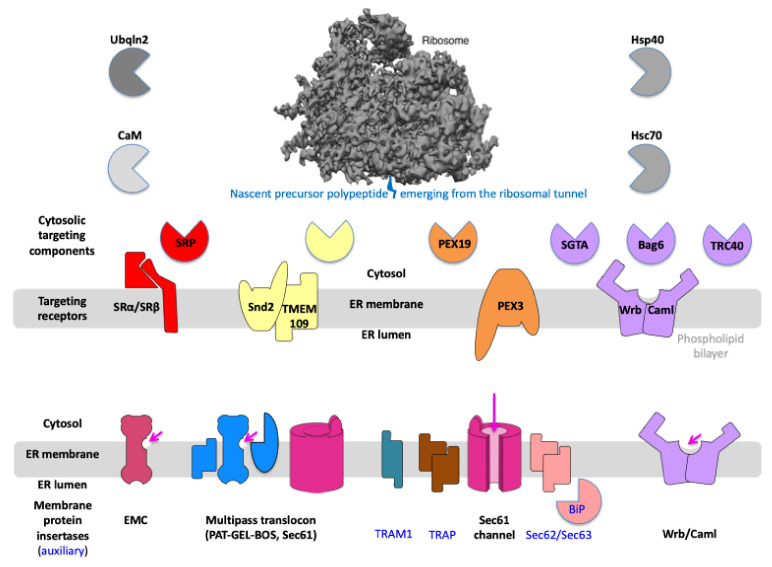
Components and mechanisms for targeting of precursor polypeptides to receptors and membrane protein insertases in the ER membrane. Nascent or fully synthesized precursor polypeptides are targeted to the ER, depending on their topogenic sequences and targeting pathways, which involve cytosolic components as well as their corresponding receptors in the ER membrane, such as SRα/SRβ, Snd2/TMEM109, PEX3/(PEX16), and Wrb/Caml. The membrane translocation is mediated by the heterotrimeric and polypeptide-conducting Sec61 channel, which may be supported by either the TRAP complex or the Sec62/Sec63 complex. Membrane insertion is mediated by several MP insertases, i.e., (i) the Sec61 channel, (ii) the Sec61 channel with the supercomplex comprising the heterodimeric intramembrane chaperone PAT, the heterotrimeric GEL, and the heterotrimeric BOS (together termed multipass translocon or MPT), (iii) the decameric EMC, or (iv) the heterodimeric Wrb/Caml complex. The long arrow (in magenta) points to the open aqueous channel and open lateral gate, respectively, of the fully open Sec61 channel; the short arrows (in magenta) point to the characteristic hydrophilic vestibules of the MP insertases that are related to Oxa1 (specifically the subunits EMC3, TMCO1, and Wrb) (Table S1). Notably, (i) calmodulin (CaM) and Hsp40 plus Hsc70 are additional cytosolic chaperones for precursor polypeptides, such as small presecretory proteins [81,90], (ii) the cytosolic N-terminus of Sec61α comprises an IQ-motif that may serve as CaM binding site [153] and, therefore, CaM was proposed to act in targeting [90], (iii) according to structural prediction, the yeast and human Snd2 may also be able to form a hydrophilic vestibule near the cytosolic surface of the ER membrane and, therefore, may also be able to facilitate membrane insertion [154,155,156], (iv) PEX3 is present in an ER subdomain which may be related to the pre-peroxisomal ER and/or ER exit sites (ERES) [157], (v) there are functionally ill-characterized homologs of Sec61α1 and TRAM1 present in human cells (Sec61α2, TRAM1L1 and TRAM2) (vi) orthologs of BiP, Sec61α1, Sec61γ, Sec62, Sec63, are essential in yeast but not orthologs of Caml, Sec61β, Snd1, Snd2, SRα, SRβ, Wrb, (vii) synthetic lethality in yeast was observed for SND2 or SND3 in combination with SEC65-1ts as well as for GET and SND pathway genes, such as SND2 and GET3, and GET and EMC genes [154], and (viii) orthologs of GEL-BOS, TRAM1, heterotetrameric TRAP, are not kown in yeast, (ix) SGTA interacts with the ß subunit of NAC according to https://thebiogrid.org (accessed on 15 May 2025), (x) in yeast Sec62 and Sec63 are present in a heterotetrameic complex together with Sec66 and Sec67, which do not have orthologs in mammalia. The Figure was adapted from reference [156].

In the 1980s, biochemical studies in cell-free systems led to the discovery of the first components of ER protein import in mammals. These were the cytosolic signal recognition particle (SRP) and its heterodimeric receptor in the ER membrane (SR), which typically ensure cotranslational and GTP-dependent targeting of nascent polypeptide chains with N-terminal SPs and the translating ribosomes to the ER (Figure 3 and Table S1) [5,48,49,50,51,52,53,54,55,56,57,58,59,60,61,62,63,64,65]. Around the same time, R. Schekman began characterizing yeast mutants defective in protein secretion, which were accordingly termed SEC mutants [4,66,67,68,69,70,71,72,73,74,75,76]. Some of these complementation groups encoded components of ER protein import, including, e.g., orthologs of SRP and SR subunits. Furthermore, the work in yeast (*Saccharomyces cerevisiae*) led to the realization that posttranslational protein import into the ER also occurs, which has also been observed for small presecretory proteins in the established mammalian cell-free systems [77,78,79,80,81,82,83,84,85,86,87,88,89,90,91,92,93,94,95,96,97,98,99]. In both systems, molecular chaperones of the HSP70 protein family were found to be involved in this posttranslational and ATP-dependent transport in both the cytosol (Hsc70) and in the ER lumen (BiP) [66,69,70,71,72,73,74,77,80,83,85,88,91,95].

In the 1990s, the heterotrimeric Sec61 complex was discovered in both experimental systems and functionally characterized by T. Rapoport, with the successful reconstitution of the cotranslational ER import of a presecretory protein into proteoliposomes containing purified Sec61 complex and SR representing a milestone (Figure 1C and Figure 3) [44,45,67,68,100,101,102,103,104,105,106,107,108,109,110,111,112,113,114,115,116,117,118]. In yeast, the ER MPs Sec62 and Sec63 were discovered as interaction partners of the Sec61 complex, which, in cooperation with BiP, supports its transport activity, as observed later for small presecretory proteins in human cells [67,68,69,70,71,86,87,89,92,93]. Furthermore, MPs with C-terminal TMH also became the focus of interest, leading to the designation of these proteins as tail anchor(ed)-or TA MPs [33,34,35,36,39,40,41,42,119,120,121,122,123,124,125,126,127,128,129,130,131,132,133,134,135] and, thus, the identification of a second protein type that does not enter the ER cotranslationally via SRP and SR.

In the first decade of this century, G. Blobel succeeded in elucidating the architecture of the yeast Sec61 complex using 3D-reconstruction after cryo-electron microscopy and single particle analysis, which has led to a large number of further structural analyses with ever higher resolution up to today [44,45,105,111,114,115,116,117,118]. The high-resolution 3D reconstructions of the Sec61 complex were facilitated by T. Rapoport’s success in crystallizing an orthologous complex from archaea in the 2000s and elucidating its structure at the atomic level using X-ray crystallography [107]. Structural biology work in recent years culminated in the successful elucidation of the architecture of the Sec61 complex and its interaction partners OST, SPC, and TRAP in human cells using cryo-electron tomography (Figure 1B–D) [20,21,22,44,45,110,115,116,117,118,136,137,138,139,140,141]. This work also employed human cells whose individual components had been depleted using siRNA or CRISPR/Cas9, allowing the assignment of individual electron densities by differential image analysis. Interestingly, such engineered cells can also be used for functional analyses in the form of semipermeabilized cells in the established cell-free system for studying ER protein import [142]. This further milestone is of particular interest as it also enables the functional characterization of mutated forms of the Sec61 complex and components such as Sec62, Sec63, and TRAP (Figure 3), which are associated with human hereditary diseases, such as Sec61 channelopathies [46,47,143,144,145,146,147,148,149,150,151,152].

In the 2000s, the Sec61 complex from mammalian cells, purified and reconstituted into proteoliposomes, was found to possess ion conductivity, which had previously been described in connection with ER protein import for ER-derived microsomes [100,106]. Furthermore, the open Sec61 channel was characterized at the cellular level as a passive ER membrane-resident calcium efflux channel [153,158,159,160]. If left uncontrolled, this ion conductivity of the Sec61 complex can apparently have pathophysiological consequences, for example, in some of the above-mentioned Sec61 channelopathies [46,47,91,143,144,145,146,147,148,149]. In addition to the medical significance of the ion conductivity of the Sec61 channel, its observation also contributed to the refinement of the concept of Sec61 gating, i.e., the idea that the complex exists in at least two states, namely the open and the closed state, and that various allosteric effectors of the channel can assist gating, namely BiP, Calmodulin, Sec62, Sec63, and TRAP [91,95,110,115,153,160,161,162]. Apparently, the open state is responsible for ER protein import, and the closed state maintains the steep gradient of calcium concentrations between the cytosol and the ER lumen. Both states, as well as at least one transition state termed primed state, have now been structurally characterized, confirming the original mechanistic predictions of a heterotrimeric channel protein consisting of two similarly structured halves that open and close a central aqueous pore by rigid body movement [44,45,107,109,110,111,112,113,114,115,116,117,118].

Furthermore, additional new components of ER protein import were discovered, including several receptors for mRNAs, 60S ribosomal subunits, and 80S ribosomes in the ER membrane (Figure S1 and Table S1) [163,164,165,166,167,168,169,170,171,172], as well as the components involved in the targeting and membrane insertion of TA proteins. The latter components include the heterodimeric Wrb/Caml complex in the ER membrane, which functions both as a targeting receptor for TA proteins in complex with their cytosolic interaction partners Bag6 complex (with TRC35, Ubl4a, and Bag6) and TRC40, and as a stand-alone Oxa1-type MP insertase (Figure 3 and Table S1) [34,36,39,119,120,121,122,123,124,125,126,127,128,129,130,131,132,133,134,135]. These components were also discovered simultaneously in yeast and in mammalian cells and apparently also target certain precursor proteins to the Sec61 complex [94,134]. In addition, an SRP-independent targeting pathway, termed the SND pathway, was discovered in yeast and also involves a cytosolic protein (Snd1) and a heterodimeric receptor in the ER membrane (Snd2/Snd3) [154,155,156,173,174,175]. This pathway apparently prefers MPs of all kinds as clients, and Snd2 also exists in human cells (Figure 3 and Table S1). Recently, yet another targeting pathway was discovered for human cells, which, in addition to the cytosolic protein PEX19, also involves the peroxisomal and ER membrane protein PEX3. This pathway directs not only peroxisomal MPs but also hairpin (HP) proteins of the lipid droplets and the ER membrane to subdomains of the ER (Figure 3 and Table S1) [8,37,38,40,135,157]. Concurrent work in yeast and mammalian cells described the ER membrane complex (EMC) as a heterodecameric MP insertase that can function independently or in cooperation with the Sec61 complex. Work in human cells characterized the PAT-GEL-BOS-supercomplex as an insertase that functions in cooperation with the Sec61 complex in the multipass translocon (MPT) (Figure 3 and Table S1) [44,118,176,177,178,179,180,181,182,183,184,185,186,187,188,189,190]. Remarkably, subunits EMC3 within EMC and TMCO1 within GEL, as well as Wrb, belong to the Oxa1 protein family of MP insertases, which are characterized by a hydrophilic vestibule on the cytosolic membrane surface instead of an aqueous pore with a lateral gate as present in the Sec61 channel (Figure 3) [31,176,185].

**Figure 4:**
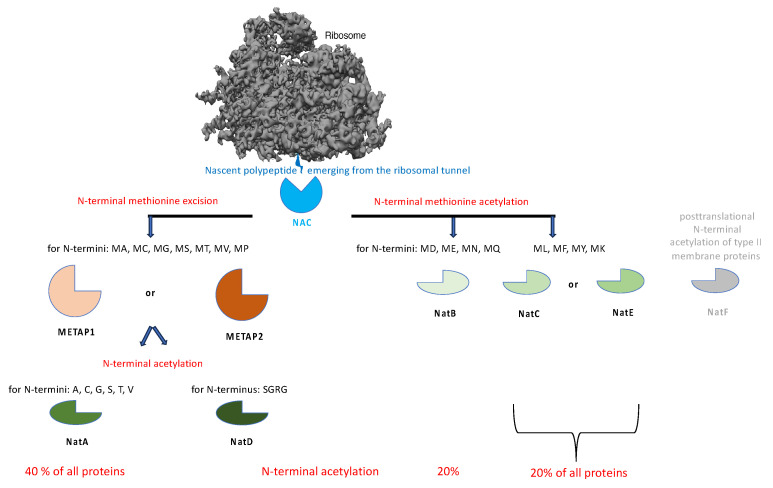
Molecular chaperones and enzymes for covalent N-terminal modification of nascent polypeptide chains in the cytosol. Binding of the nascent polypeptide, as it emerges from the ribosomal exit tunnel, to NAC is mutually exclusive with binding to SRP and the Sec61 complex [59,193,194]. This guarantees that complexes of ribosomes and nascent chains with SP or TMH are targeted to the Sec61 channel in the ER membrane and that complexes of ribosomes and nascent cytosolic polypeptides that were initiated at ER-bound ribosomes are released from Sec61 [192]. Interestingly, the α subunit of NAC recruits SRP to complexes of ribosomes and nascent chains with SP or TMH [194], and the ß subunit of NAC can interact with SGTA, which is a component of the TRC/GET pathway (https://thebiogrid.org (accessed on 15 May 2025)). METAP, methionine aminopeptidase; NAC, nascent polypeptide associated complex; Nat, N-acetyltransferase. The Figure layout was inspired by reference [194].

Recent work in both experimental systems has also shown that the nascent chain-associated complex (NAC) coordinates N-terminal methionine excision and N-terminal acetylation of the majority of nascent polypeptides in the cytosol (Figure 4 and Table S1) [191,192,193,194,195,196,197,198,199]. Based on protein databases, an estimated 80% of all cellular proteins are enzymatically modified in this way, including MPs [194,196,197]. However, some of the latter are thought to be posttranslationally N-terminally acetylated in the Golgi apparatus [199]. In short, the heterodimeric chaperone NAC, which is equimolar to ribosomes in the cytosol and is typically bound to them (Table S1), binds first to the nascent chains during polypeptide synthesis as they emerge from the ribosomal tunnel exit. It then recruits one of the two methionine aminopeptidases (METAPs) and subsequently one of several N-acetyltransferases (NATs). In both cases, substrate specificity is determined by the amino acid residue following the initiating methionine (Figure 4) [194]. Alternatively, the initiating methionine is retained and becomes N-acetylated. SRP can antagonize NAC binding in the case of SP-containing precursor polypeptides of secretory and membrane proteins, thus inhibiting cotranslational N-terminal methionine excision and N-terminal acetylation. Apparently, the hydrophobic SPs trigger NAC release through direct interaction. Notably, in yeast, the artificial enzymatic modification of an SP was observed to inhibit ER import of the polypeptide [195]. However, as noted above, many TMHs of MP precursors are not N-terminal, raising the question of whether or not these precursors can undergo these particular enzymatic modifications. Regardless, many nascent polypeptides are covalently modified by the above-mentioned enzymes SPC (SPC-A or SPC-C) and OST (specifically, OST-A) on the lumenal side of the ER membrane (Figure S2 and Table S1).

**Figure 5:**
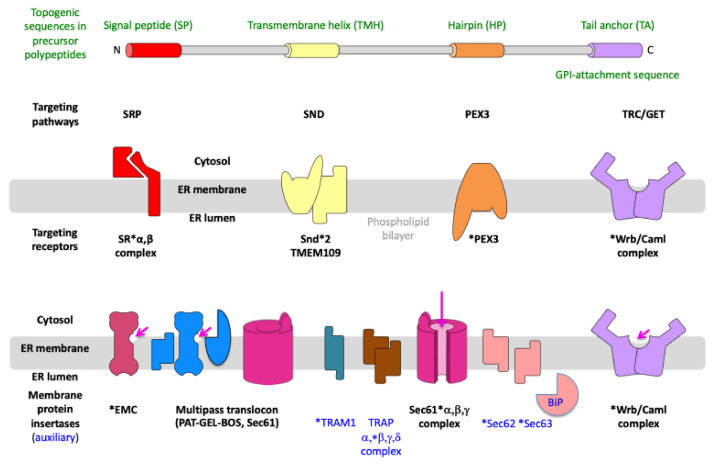
Targeting of precursors of membrane proteins to and insertion into the ER membrane depending on the position of their topogenic sequences. The asterisks (*) highlight the components that were addressed by depletion in human cells in combination with quantitative MS and differential protein abundance analysis by us [98,156,157,200,201] and others (EMC) [178,179]. See text and Figure 3 for details.

Although MPs account for one quarter of polypeptides in human cells and sixty percent of human drug targets, little is known about substrate specificities and affinities of most of the components that enable membrane-targeting and -insertion of MP precursors, putative pathway overlaps, and regulatory mechanisms. The expectation is that a complete picture of this cell biological problem will eventually define the biosynthetic pathways of already known drug targets, as well as potential novel ones that are involved in the biogenesis of certain MP types. About ten years ago, we started to combine knock-down or knock-out of various of the above-mentioned components in human cells with label-free quantitative mass spectrometric (MS) analysis of the total proteomes of depleted and non-depleted cells. This was followed by differential protein abundance analysis to identify putative clients of components involved in targeting or membrane insertion of precursor polypeptides into the human ER under cellular conditions (Figure 5) [200]. The timing of these experiments was such that the cellular integrity was affected as little as possible, which resulted in relatively low numbers of negatively affected precursors. Nevertheless, the properties of the client polypeptides were analyzed using various online tools to identify putative substrate specificities and possible reasons for the underlying rules of engagement. The original results [98,156,157,170,200,201] as well as the first comparative analyses have been published previously [18,202]. Furthermore, these publications noted that the analysis confirmed the detection of representatives of certain protein classes among the substrates of a given component. In addition, the proof-of-principle publication confirmed through quantitative RT-PCR that the mRNAs of TRAP clients remained unaffected by TRAP silencing and through mutagenesis studies that TRAP indeed supports ER import of SP-bearing precursors, including type I MPs with high glycine/proline- or GP-content and therefore low alpha-helical propensity in their SPs [200]. The focus in this article/review-hybrid is on MP clients, i.e., general lessons from and limitations of the proteomic approach to find answers for the cell biological problem at hand. Furthermore, novel and previously neglected aspects are addressed, such as N-terminal methionine excision plus N-terminal acetylation and the effect of the number of TMDs in MP clients, which were identified in our proteomic analyses.

## 2. Results

Our unbiased experimental approach combined the knock-down of the respective targeting (SRα, Snd2, Wrb, PEX3) or membrane insertion component (Sec61α, Sec62, Sec63, TRAM1, TRAPβ) using two different siRNAs in separate HeLa cell pools for 96 h (Figure S3A) with label-free quantitative MS of the total proteomes of depleted and non-depleted cells, followed by differential protein abundance analysis [110,116,128,137,138,139,140,141]. The MS data have been deposited with the ProteomeXchange Consortium with the data set identifiers indicated in Table S2. Typically, siRNA-mediated depletion resulted in > 90% depletion of the target component without substantially affecting cell growth and viability, as well as cellular and ER morphology. To identify additional clients, separate CRISPR/Cas9-mediated knock-outs were performed for Sec62 and Sec63 in HEK293 cells instead of siRNA-mediated knock-down (Figure S3B). For TRAP and PEX3, fibroblasts from patients with Congenital Disorders of Glycosylation and Zellweger Syndrome patients, respectively, were analyzed in parallel with control fibroblasts (Figure S3C) [137,141]. We also interrogated the clients of three putative components for mRNA- and/or ribosome-targeting to the ER (ERj1, KTN1, RRBP1).

### 2.1. Identification and Classification of Putative Clients

**Figure 6:**
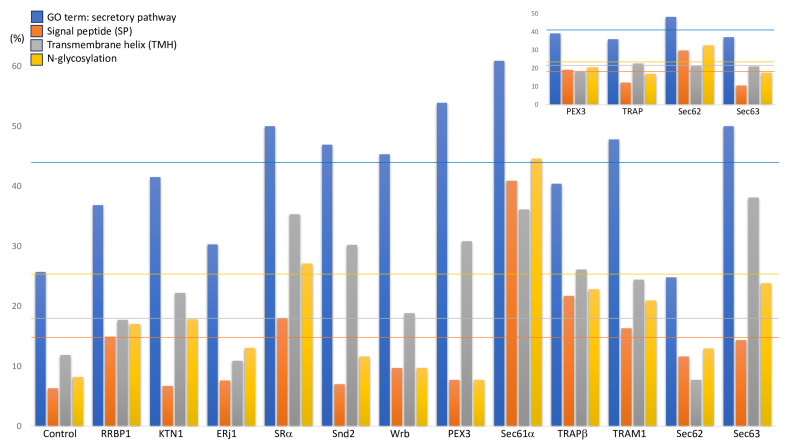
Comparative client characteristics. The clients of the indicated components for targeting of precursor polypeptides to the ER and their membrane insertion were determined by quantitative MS and differential protein abundance analysis following siRNA-mediated depletion of the respective component, as outlined in Figure S3A. Alternatively, knock-out cell lines were employed as shown in Figure S3B,C, and the results are shown in the insert on the upper right. Clients were defined as such by the presence of an SP or at least one TMH, and where applicable by N-glycosylation (see legend in insert on the upper left). Original data are given in references [98,156,157,170,200,201]. The respective average values are indicated by a line in matching colors to the legend insert.

The approach relied on the expectation that precursor polypeptides are degraded by the cytosolic proteasome upon interference with their biogenesis. Therefore, their cellular concentrations are negatively affected in the two separate pools of depleted cells compared to control cells. This change was demonstrated using quantitative MS combined with differential protein abundance analysis (Figure S3) [137,200]. Typically, 5000 to 6500 different proteins were quantified and statistically analyzed [200]. Under conditions of depletion of an ER protein import component, Gene Ontology (GO) terms assigned on average 44% of the negatively affected proteins to organelles of the endocytic and exocytic pathways, corresponding to an average 1.7-fold enrichment over the 25.7% of the control cells. A slightly higher enrichment of precursor proteins with SP (average 2.4), TMH (2.1), and N-glycosylation (2.3), respectively, was observed (Figure 6) [200].

**Figure 7:**
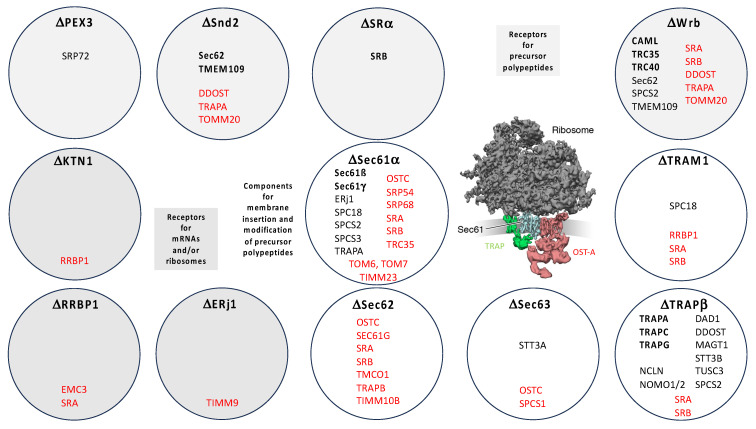
Secondary negative and positive effects of component depletion. The cartoon summarizes all our depletions and highlights physical (shown in black) as well as putative genetic interactions (shown in red). The inset of the OST-A translocon illustrates some of the physical interactions between its subunits. In the case of Wrb depletion, the increase in SR was only detected by Western blot; in the case of Snd2, the decrease in Sec62 and TMEM109 was only detected after the simultaneous depletion of Snd2 and Wrb as was the increase in DDOST, TRAPA and TOMM20. Notably, this observed decrease in TMEM109, together with reciprocal co-immunprecipitations of Snd2 and TMEM109 and bimolecular luminescence complementation (nano-BiT) in cells, led us to the suggestion of TMEM109 as a Snd3 candidate in humans [156]. The following numbers of additional genetic interactions were observed for ubiquitin related enzymes after depletion of ERj1 (2), KTN1 (0), RRBP1 (4), PEX 3 (0, 1 for ko), SRα (0), Snd2 (3), Wrb (6), Snd2 plus Wrb (7), TRAM1 (2), TRAPβ (2, 3 for ko), Sec61α (11), Sec62 (11, 4 for ko), Sec63 (0, 1 for ko) (Table S17). SRP knock-out cells showed depletion of SPCS1 and OST subunits [172]. Original data are given in references [98,156,157,170,200,201].

The absence of the target components, typically, also led to degradation of the other subunit(s) of the target complex or even other components of the same pathway, confirming physical interactions as well as pointing towards potential novel ones (Figure 7, in black) [200]. Furthermore, the depletion of the target components was accompanied by various positively affected proteins, such as an increase in ubiquitin-conjugating enzymes in the cytosol (Figure 7, legend), and in some cases an increase in other protein transport components, which could indicate genetic interactions between pathways (Figure 7, in red) [200]. All these phenomena correlated inversely with the severity of the negative effects on secretory pathway proteins. Only after depletion of Sec61, which had the strongest effect on ER protein import (resulting in enrichment factors of 2.4, 6.5, 5.4, and 3.1, respectively), did we observe an increase in components for protein import into mitochondria (TOM6, TOM7, TIMM23), which appears to be an alternative to protein degradation in the cytosol [200]. However, there was no evidence of ERAD or UPR activation in the depletions shown in Figure 7. Collectively, these results confirmed the validity of the experimental approach.

### 2.2. Weaknesses of the Experimental Approach

The interpretation of the proteomic data was clearly weakest where the total number of negatively affected MPs of the secretory pathway, and thus of putative MP clients, was lowest. This was the case of KTN1 and ERj1 depletion, with a number of 9 and 11 putative MP clients, respectively (Tables S3–S5). Therefore, I refrained from a detailed discussion of the results on clients of mRNA- and ribosome-receptors. Another potential weakness of the approach concerns depletions that did not lead to an increase in the enrichment factors discussed above (GO term, SP, TMH, N-glycosylation) and were therefore not previously considered. This issue will be discussed based on two experiments, one involving BiP depletion and the other a hitherto unpublished Snd2/Wrb double depletion.

In contrast to Sec62 and Sec63 depletion, *HSPA5* silencing was expected a priori to lead to more complex results, as BiP, which is encoded by the *HSPA5* gene, is involved not only in ER protein import but also in the folding and assembly of newly imported proteins. It has previously been shown that transient *HSPA5* silencing for 48 h resulted in approximately 75% BiP depletion without significantly affecting cell growth, cell viability, or cell/ER morphology [91]. To increase silencing efficiency, cells were treated with two different *HSPA5*-targeting siRNAs or a non-targeting siRNA in triplicate for 72 h. The efficacy of silencing was confirmed by Western blot. After BiP depletion for 72 h, 5543 different proteins detected in all samples were quantitatively characterized by MS (Figure S4, Tables S9–S12). Applying established statistical analysis, we found that transient and partial BiP depletion significantly affected the steady-state levels of 746 proteins: 340 negatively and 406 positively (q < 0.05). As expected, BiP itself was negatively affected (Figure S4A), which was confirmed by Western blot (Figure S4B). Of the negatively affected proteins, GO terms assigned approximately 27.5% to organelles of the endocytic and exocytic pathways (Figure S4C, large pies). We also did not detect significant enrichment of proteins containing SP, N-glycosylated proteins, and MPs (Figure S4C, small pies). However, the affected precursors included 33 proteins with cleavable SP (including the Sec62 clients FKBP10, FKBP14, SUMF1, and TAPBP) and 22 MPs with an amino-terminal TMH (including the Sec62/Sec63-complex client SLC20A2) (Figure S5). As mentioned above, however, these precursors may not necessarily be affected during ER import, but rather during their subsequent folding or assembly, which, if failed, is also expected to lead to proteasomal degradation.

Proteins positively affected by BiP depletion for 72 h included SRP and SRP receptor subunits (SRP54, SRP68, SRPRA, SRPRB) (Figure S4A and Table S12), the mRNA and ribosome receptor RRBP1, and various components for protein import into mitochondria (TIMM2, TIMM44, TOMM6). This is all consistent with precursor accumulation in the cytosol due to ER import defects following BiP depletion (Table S12). Furthermore, *SEC63* and the *DNAJC1* (encoding the putative Sec63 paralog ERj1) were positively affected by BiP depletion, again suggesting ER import defects due to BiP depletion (Table S19). The latter may also apply to the overproduction of ten ubiquitin-conjugating enzymes (UBE3A, UBE20, UEVLD) and ligases (ITCH, KCMF1, MID1, NEDD4, RNF13, UBR1) (Table S12), which is consistent with increased proteasomal degradation. Furthermore, there was considerably evidence of activation of the unfolded protein response (UPR) during the 72 h knock-down, i.e., typical UPR-regulated genes such as *ATF6* (encoding the UPR regulator and transcription factor ATF6), *HSP90B1* (encoding the ER lumenal chaperone Grp94)*, HYOU1* (encoding the ER lumenal chaperone Grp170), and *SEL1* (encoding the ERAD component Sel1) were up-regulated (Table S12). These results are consistent with the original expectation that BiP depletion for 72 h also impairs protein folding and assembly.

We previously reported that transient Snd2/Wrb double depletion for 96 h resulted in approximately 90% depletion of both target molecules without significantly affecting cell growth, cell viability, or cell/ER morphology [156]. In an unpublished second Snd2/Wrb double depletion, cells were treated with a different combination of targeting siRNAs as before or a non-targeting siRNA in triplicate for 96 h. Silencing efficiencies were confirmed by Western blot. After double depletion for 96 h, 6052 different proteins were quantitatively characterized by MS and detected in all samples (Figure S6, Tables S13–S16). Using established statistical analysis, we found that the novel transient double depletion of Snd2/Wrb significantly affected the steady-state levels of 1286 proteins: 494 negatively and 792 positively (q < 0.05). As expected, the Wrb proxy Caml was negatively affected (Figure S6A), which was indirectly confirmed by Western blot analysis for Wrb (Figure S6B). Of the negatively affected proteins, GO terms assigned approximately 26.1% to organelles of the endocytic and exocytic pathways (Figure S6C, large pies). We also did not detect significant enrichment of proteins containing SP, N-glycosylated proteins, and MPs (Figure S6C, small pies). However, the affected precursors included 16 proteins with cleavable SP (including the established Wrb clients FAM172A, TGFB1, TMX3, TPP1) and 37 MPs with amino-terminal TMH (including the Wrb clients EXT2, GOLGA5, MARCH1, MFSD7, PET100, TMEM126B, UBE2J2) (Figure S7).

Proteins positively affected by the novel Snd2/Wrb double depletion for 96 h included both SRP receptor-subunits (SRA, SRB) plus PEX3 (Figure S6A, Tables S16 and S17), the mRNA and/or ribosome receptors RRBP1 and ERj1, several MPT- or EMC- subunits (ASTERIX, NOMO2, OPTI, SEC61G; EMC3, EMC7, EMC10) and various components for protein import into mitochondria (MTCH1, OXA1L, TIMM13, TIMM17A, TOMM1L1, TOMM22, TOMM40), all consistent with extensive precursor accumulation in the cytosol due to ER import defects (Table S16). The latter is also consistent with the observed overproduction of approximately twenty ubiquitin-conjugating enzymes (such as UBE2A, UBE2B, UBE2G1, UBE2K, UBE2L3, UEVLD) and ligases (including HERC1, HLTF, ITCH, KCMF1, MID1, NEDD4L, RBBP6, RNF5, RNF25, RNF138, SMURF2, UBR1), plus SUMO-conjugating UBA2A (Tables S16 and S17). However, the upregulation of autophagy-, ERAD- and apoptosis-related components (ATG101; DERL2, SEL1; BCL2L2, CASP9, PDCD2, PDCD4) as well as of UPR-controlled genes (such as *HSPA5*) suggests the development of a severe cellular defect (Tables S16 and S17).

Thus, there were good reasons not to interpret the results of the previously published BiP depletion [98] and the novel concurrent Snd2/Wrb depletion in the context of protein biogenesis at the ER. Furthermore, these two data sets confirmed that the used enrichment factors for GO terms, SP- and TMH-content, and N-glycosylation are valid criteria for successful experiments that are directed at the identification of substrates of ER protein import components (Figure 7).

### 2.3. Clients in Precursor Polypeptide Targeting to the Human Endoplasmic Reticulum

#### 2.3.1. The SRP/SR Targeting Pathway

The SRP/SR pathway represents the archetypal cotranslational targeting mechanism and delivers nascent precursor polypeptides early in their synthesis to the insertion and translocation machinery in the ER membrane [48,49,50,51,52,53,60,61,62,63]. Additionally, SRP may play a general role in mRNA targeting to the ER [172].

**Figure 8:**
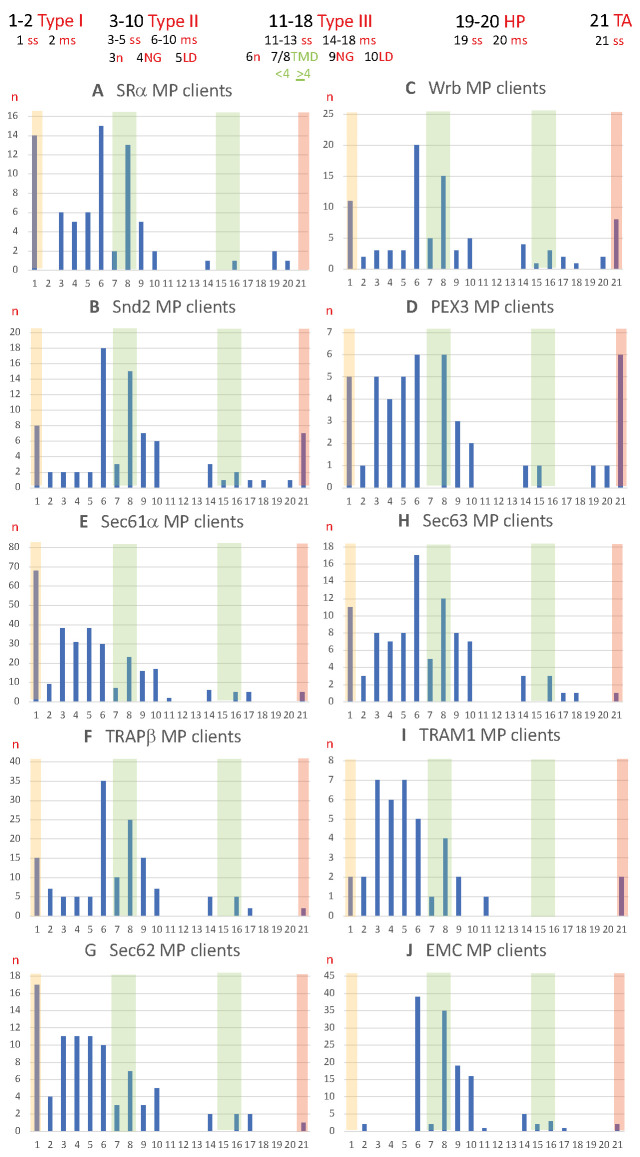
Details of membrane protein client characteristics. (**A**–**J**) The clients of the indicated components for targeting of precursor polypeptides to the ER (**A**–**D**) and their subsequent translocation or membrane insertion (**E**–**J**) were determined by quantitative MS and differential protein abundance analysis following depletion of the respective component as outlined in Figure S3. Clients were defined as such by the presence of either an SP or at least one TMH. To characterize the clients of the various targeting and insertion components, the absolute numbers of SP and TMH containing clients were calculated as given in Table S4 and converted to percent values as given in Table S5. The legend of the figure is illustrated on the top for all bar diagrams, single-stranded type I MPs are highlighted in yellow, multispanning type II and type III MPs, respectively, with either <4 or ≥4 TMDs in green, and TA proteins in red. LD, ER lumenal domain(s) with >50 amino acid residues; ms, multispanning; n, total number; NG, N-glycosylation; ss, single-spanning. The number of TMDs in MP precursors was determined in our proteomic data sets for the first time.

In the MS approach, the SR clients included 25% precursors of single-spanning type I MPs but no multispanning type I MPs. Furthermore, they included 57.1% precursors of MPs with TMH (Figure 8A and Table S3) [156]. Thus, 82.1% of all SR clients were MPs; the remaining 17.9% were precursors of soluble proteins. The precursors with TMH included 19.6% single-spanning MPs (11.1% known type II), 32.1% multispanning MPs (27.8% type II and 1.9% type III), and 5.4% HP proteins (3.6% single-spanning and 1.8% double-spanning) (Figure 8A, Tables S3–S6). The discrepancy between the percentages of single-spanning and multispanning MPs and their subpopulations (indicated in parentheses) is due to the problem that the topology remains unknown in some cases, as is also true for the presence of large ER lumenal domains (see below). When the single-spanning type II precursors with TMH were analyzed for the presence of ER lumenal domains with more than 50 amino acid residues, the number was 11.1%, meaning all MPs of this class had a large ER lumenal domain [202]. When the multispanning precursors with TMH were analyzed for further details, i.e., number of TMDs (including the TMH) and the presence of large ER lumenal domains, the numbers for type II MPs were 3.7% below four TMDs and 24.1% above four TMDs, and 3.7% had large ER lumenal domains. The number for the type III MPs was 1.9% above four TMDs. Thus, for MPs, a general preference for SR was found for single-spanning type I MPs and for multispanning type II MPs with four or more than four TMDs, but hardly any large ER lumenal domains (13% of multispanning type II MPs).

**Figure 9:**
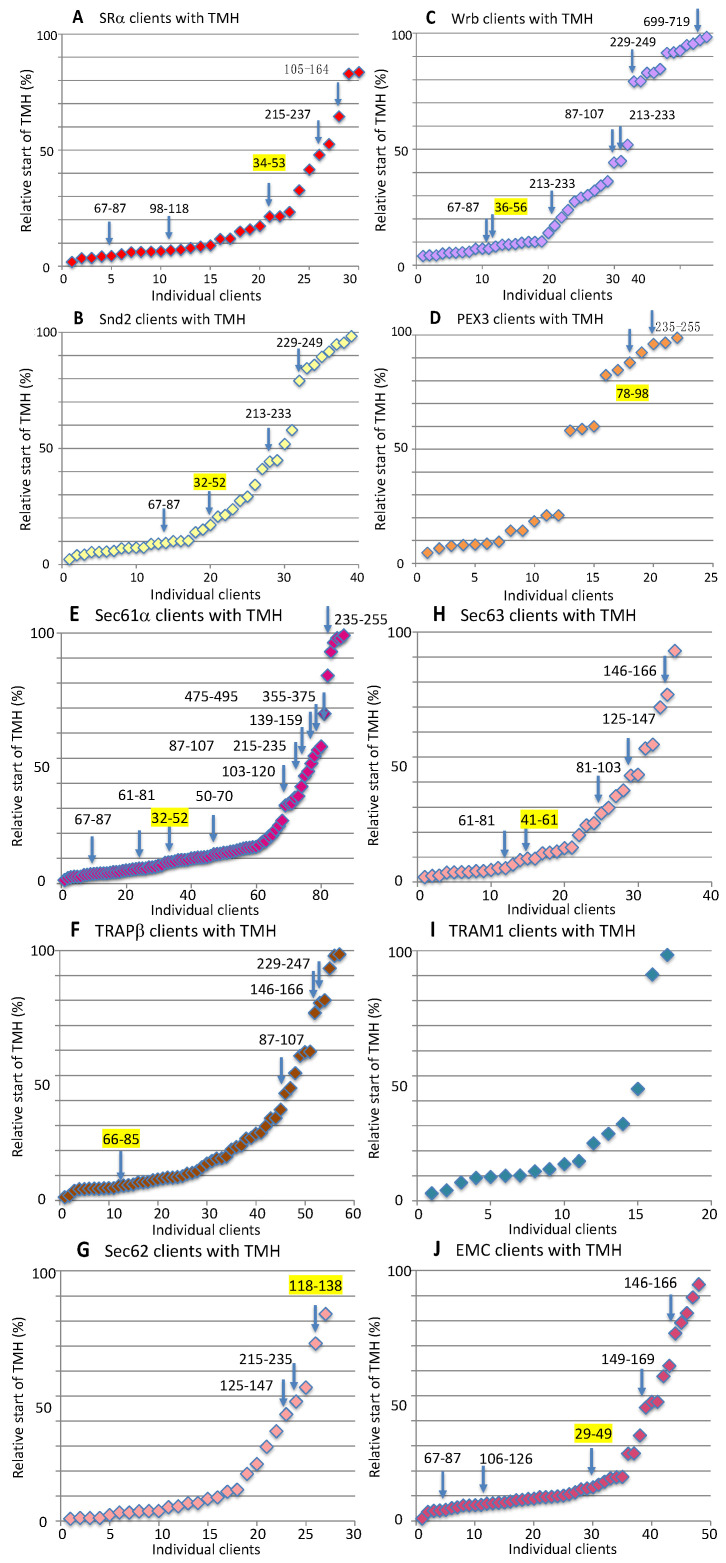
N-terminal methionine excision and N-acetylation of membrane protein clients. (**A**–**J**) TMH containing MP clients of the indicated components for targeting of precursor polypeptides to the ER (**A**–**D**) and their subsequent membrane insertion (**E**–**J**) were plotted against the location of their TMH, i.e., position of the central amino acid residue of TMH in % of client amino acid residues. (**D**) PEX3 clients refer to the pool of clients that were detected after PEX3 depletion in HeLa cells, plus in the Zellweger patient fibroblasts with a PEX3 deficiency, and are shown in Table S6. (**F**) TRAP clients refer to the pool of clients that were detected after TRAP depletion in HeLa cells plus in the CDG patient fibroblasts with a TRAP deficiency, due to either TRAPγ or TRAPδ deficiency, and are shown in Table S8; (**G**,**H**) Sec62 and Sec63 clients, respectively, refer to the pool of clients that were detected after knock-down in HeLa cells plus knock-out in HEK293 cells and are shown in Table S8. Clients were screened for N-terminal methionine excision and N-acetylation in the NCBI protein database (https://www.ncbi.nlm.nih.gov/protein (accessed on 1 March 2025)). MPs with N-terminal methionine excision plus N-acetylation are highlighted by blue arrows and indicate the respective client’s most N-terminal TMH; the most N-terminal TMH of the respective client compilation is additionally highlighted in yellow. The Figure was adapted from reference [202]. The N-terminal methionine excision and N-acetylation of MP precursors were determined in our proteomic data sets for the first time.

Previous analysis of the TMHs of type II and III MPs for their positioning within the precursor proteins revealed that 77% of SR clients had comparatively N-terminal TMHs (Figure 9A and Table S6) [202]. Considering type I MPs, an SR preference for MPs with aminoterminal topogenic sequences of 80% and an overall preference of SR for aminoterminal topogenic sequences of 84% were found.

#### 2.3.2. The SRP-Independent Targeting Pathways

The SND pathway can act posttranslationally and therefore accept small presecretory proteins, small-MPs, TA-MPs, and GPI-anchored MPs as clients [154,155,156,173,174,175]. Therefore, some of these clients also involve Sec62, an auxiliary component of the Sec61 channel (see below), and TMEM109, both of which were suggested as interaction partners by our experimental approach (Figure 7). The observed depletion of TMEM109 after Snd2 depletion, together with reciprocal co-immunprecipitations of Snd2 and TMEM109 and bimolecular luminescence complementation (nano-BiT), led us to suggest TMEM109 as an Snd2 interaction partner [155,156]. See Section 3.2.1. for further discussion of this issue. Notably, however, recent work has proposed Snd2 as a player in the SRP/SR pathway [65].

The Snd2 clients included 14% precursors of single spanning type I MPs, 3.5% precursors of multispanning type I MPs, and 71.9% precursors of MPs with TMH (Figure 8B, Table S3) [156]. Thus, 89.5% of the Snd2 clients were precursors of MPs. The Snd2 clients with TMH included 10.5% single-spanning MPs (3.5% known type II), 47.4% multispanning MPs (31.6% type II and 5.3% type III), 1.8% double-spanning HP proteins, and 12.3% TA proteins (Figure 8B, Tables S3–S6). When the multispanning precursors with TMH were analyzed for further details, the numbers for the type II MPs were 5.3% with fewer than four TMDs, 26.3% with four or more TMDs, and 10.5% with large ER lumenal domains and a corresponding high proportion of N-gylcosylation (39%). The numbers for type III MPs were 1.8% for fewer than four TMDs and 3.5% for more than four TMDs, and 1.8% had large ER lumenal domains. Thus, among MPs with TMH, there was a general preference of Snd2 for multispanning type II MPs with four or more TMDs, plus, to a moderate extent, large ER lumenal domains (33% of multispanning type II MPs) and for TA proteins. The average hydropathy of the seven TAs was 2.864.

Previous analysis of TMHs for their positioning within the precursor proteins revealed that 42% of Snd clients possessed central or even C-terminal TMHs; for comparison, the number for SR was 23% (Figure 9B and Table S6) [202]. Considering type I MPs, Snd2 showed a 65% preference for MPs with aminoterminal topogenic sequences and a 68% overall preference for aminoterminal topogenic sequences; the respective numbers for SR were 80% and 84%.

Although the ribosome-associated Bag6 complex is also involved, the TRC/GET system was expected to act posttranslationally due to the carboxy-terminal location of the TA [33,34,35,36]. Therefore, it can also accept small presecretory proteins as clients [94,95,96] and also target HP proteins to the ER in yeast [135]. The Wrb clients included 13.9% precursors of single-spanning type I MPs, 2.5% precursors of multispanning type I MPs, and 64.4% precursors of MPs with TMH (Figure 8C and Table S3) [156]. Thus, 82.2% of the Wrb clients were precursors of MPs. The precursors with TMH included 8.2% single-spanning MPs (4.1% known type II), 42.5% multipanning MPs (27.4% type II and 5.5% type III), 2.7% double-spanning HP proteins, and 11% TA proteins (Figure 8C, Tables S3–S6). When analyzing the multispanning precursors with TMH for further details, the numbers for type II MPs were 6.8% below and 20.5% above the four TMDs, and 6.8% had large ER lumenal domains. The numbers for the type III MPs were 1.4% below and 4.1% above the four TMDs, and 1.4% had large ER lumenal domains. Thus, for MPs, Wrb showed a general preference for multispanning type II MPs with four or more TMDs plus, to a moderate extent, large ER lumenal domains (25% of multispanning type II MPs) and for TA proteins. The average hydropathy of the eight TAs was 2.464.

Previous analysis of TMHs for their positioning within the precursor proteins revealed that 47% of Wrb clients possessed central or even C-terminal TMHs, which is similar to Snd2 (Figure 9C and Table S6) [202]. Considering type I MPs, Wrb showed a 57% preference for MPs with aminoterminal topogenic sequences and an overall preference for aminoterminal topogenic sequences of 67%. These results suggested a more general targeting role of the TRC pathway than previously assumed. Initial evidence in this direction, however, was provided by the observation that SGTA, a component of the cytosolic arm of the TRC pathway, is cotranslationally recruited to ribosomes that synthesize a variety of MPs, including those with a cleavable SP (Figure 3 and Table S1) [134].

The PEX19/PEX3 pathway has been characterized as yet another pathway for targeting of precursor polypeptides to the ER and their subsequent insertion into the ER membrane [7,8]. These precursor proteins include HP proteins that persist in the ER or are pinched off as part of lipid droplets [37,38]. As mentioned previously, other HP proteins have been observed to involve the GET pathway in yeast (Erg1) [135] and EMC in the human system [40].

The PEX3 clients included 9.8% precursors of single-spanning type I MPs, 2% precursors of multispanning type I MPs, and 45.1% precursors of MPs with TMH, including five peroxisomal MPs with TMH (Figure 8D, Table S3) [157]. Thus, 56.8% of the PEX3 clients were precursors of MPs. The PEX3 clients with TMH included 15.7% single-spanning MPs (10% known type II), 13.7% multispanning MPs (12% type II and 2% type III), 3.9% HP proteins (2% single-spanning and 2% double-spanning), and 11.8% TA proteins (Figure 8D, Tables S3–S6). When the multispanning precursors with TMH were analyzed for further details, the numbers for the type II MPs were 12% over four TMDs, and 4% had large ER lumenal domains. The numbers for type III MPs were 2% under four TMDs. Thus, for MPs, there was a general preference for PEX3 for single-spanning type I and type II MPs (almost 20% in total), as well as for TA proteins. The average hydropathy of the six TAs was 2.231.

A previous analysis of TMHs for their positioning within the precursor proteins revealed that 46% of PEX3 clients had central or C-terminal TMHs, which is comparable to Snd2 and Wrb (Figure 9D and Table S6) [202]. Considering type I MPs, PEX3 showed a 55% preference for MPs with aminoterminal topogenic sequences and an 78% overall preference for aminoterminal topogenic sequences.

### 2.4. Clients in Insertion of Precursor Polypeptides into the Membrane of the Human Endoplasmic Reticulum

#### 2.4.1. The Sec61 Complex as the Central Entry Gateway into the ER Membrane

The heterotrimeric Sec61 complex provides the general entry gateway for precursor polypeptides with SPs or TMHs into the ER. It is also part of the multipass- and the multipass-TRAP translocon and cooperates with EMC in membrane insertion of some precursors [44,45,107,116,117,118]. In our MS approach, the Sec61 clients included 68.9% precursors with SP (including 23.8% precursors of type I MPs and 3.1% precursors of multispanning type I MPs) and 31.1% precursors of MPs with TMH (Figure 8E and Table S7) [200]. Overall, 58% of Sec61 clients were precursors of MPs. Furthermore, the Sec61-dependent precursors with TMH included 14% single-spanning MPs (13.3% known type II and 0.7% type III), 14% multispanning MPs (10.5% known type II and 2.1% type III), and 1.7% TA proteins (Figure 8E, Tables S5, S7, and S8). The average hydropathy of the five TAs was 2.4. Notably, no HP proteins were found among the Sec61 clients. When the multispanning precursors with TMH were analyzed for further details, the numbers for the type II MPs were 2.4% below four TMDs, 8% above four TMDs, and 5.9% had large ER lumenal domains and a corresponding high proportion of N-gylcosylation (53%). The numbers for the type III MPs were 1,7% above four TMDs. Sec61 thus showed a clear preference for amino-terminal topogenic sequences and for single-spanning MPs, and to a lesser extent for multispanning type II MPs with a substantial percentage of large ER lumenal domains (57%).

The previous analysis of TMHs with regard to their positioning within the precursor proteins revealed that 77% of Sec61 clients had comparatively amino-terminal TMHs, which is similar to SR (Figure 9E and Table S8) [202]. Taking type I MPs into account, Sec61 showed a preference of 86% for MPs with aminoterminal topogenic sequences and an overall preference of 92% for aminoterminal topogenic sequences, i.e., also similar to SR.

#### 2.4.2. The Auxiliary Sec61 Components: TRAP, TRAM1, Sec62, and Sec63

TRAP was originally characterized as a heterotetrameric signal-sequence receptor (SSR) complex and a Sec61 interaction partner [136,137,138,139,140,141]. There is no obvious TRAP ortholog in yeast. TRAP clients include 12.1% type I MP precursors, 5.6% multispanning type I MP precursors, and 46% multispanning MP precursors with TMH (Figure 8F, Table S7) [200]. Thus, 63.7% of TRAP clients were precursors of MPs. The precursors with TMH included 10.5% single-spanning MPs (4% known type II and 0.7% known type III), 33.9% multispanning MPs (28.2% type II and 4% type III), and 1.6% TA proteins (Figure 8F, Tables S5, S7, and S8). The average hydropathy of the two TAs was 2.182. In the detailed analysis of the multispanning precursors with TMH, the number of type II MPs was 8.1% below four TMDs and 20% above four TMDs; 5.6% had large ER lumenal domains. The number of type III MPs was 4% above four TMDs. TRAP thus showed a preference for amino-terminal topogenic sequences and a general preference for multispanning type II MPs with more than four TMDs, which is in perfect agreement with the observation of the multipass-TRAP translocon [44].

Previous analysis of TMHs for their positioning within the precursor proteins revealed that 67% of TRAP clients had comparatively amino-terminal TMHs (Figure 9F and Table S8) [202]. Considering type I MPs, TRAP showed a 76% preference for MPs with aminoterminal topogenic sequences and an overall preference for aminoterminal topogenic sequences of 85%.

TRAM1 represents a multispanning MP of the human ER and has no obvious yeast ortholog [203,204,205,206,207]. The TRAM1 clients included 6.7% precursors of type I MPs, 6.7% precursors of multispanning type I MPs, and 56.7% precursors of MPs with TMH (Figure 8I and Table S7) [201]. Thus, 70% of the TRAM1 clients were precursors of MPs. The precursors with TMH included 33.3% single-spanning MPs (23.3% known type II), 16.7% multispanning MPs (16.7% type II), and 6.7% TA proteins (Figure 8I, Tables S5, S7, and S8). The average hydropathy of the two TAs was 2.081. In the detailed analysis of the multispanning precursors of type II MPs, the numbers were 3.3% below four TMDs and 13.3% above four TMDs. Thus, among the auxiliary components of the Sec61 channel, TRAM1 showed a clear preference for amino-terminal topogenic sequences and the highest preference for single-spanning type II MPs.

The previous analysis of TMHs with respect to their positioning within the precursor proteins revealed that 71% of TRAM 1 clients had comparatively amino-terminal TMHs, i.e., similar to SR and Sec61 (Figure 9I and Table S8) [202]. Taking type I MPs into account, TRAM1 showed a 76% preference for MPs with aminoterminal topogenic sequences and an overall preference for aminoterminal topogenic sequences of 83%, i.e., also similar to SR and Sec61.

The Sec61-associated Sec62 protein supports co- and posttranslational ER protein import in a client-specific manner, in some cases with support from its interaction partner in the ER membrane and co-chaperone, Sec63, and the ER lumenal chaperone BiP [87,88,89,91,92,93,95,96,97,113,208,209,210,211]. Sec62 clients included 16.7% precursors of type I MPs, 3.9% precursors of multispanning type I MPs, and 27.5% precursors of MPs with TMH (Figure 8G and Table S7) [98]. Thus, 48.1% of Sec62 clients were precursors of MPs. The precursors with TMH included 11.8% single-spanning MPs (10.9% known type II), 14.7% multispanning MPs (9.9% known type II and 2% known type II), and 1% TA proteins (Figure 8G, Tables S5, S7, and S8). The hydropathy of the single TA was 1.983. When analyzing the multispanning MPs for further details, the numbers for type II MPs were 3% below four TMDs and 6.9% above four TMDs, and 5% had large ER lumenal domains; the numbers for type III MPs were 2% above four TMDs. Thus, Sec62 demonstrated a clear overall preference for amino-terminal topogenic sequences (92.2%), especially SPs, and for multispanning type II MPs with a significant proportion of large ER lumenal domains (50%).

Previous analysis of TMHs with respect to their positioning within the precursor proteins revealed that 74% of Sec62 clients had comparatively amino-terminal TMHs, i.e., similar to SR and Sec61 (Figure 9G and Table S8) [202]. Considering type I MPs, Sec62 showed an 84% preference for MPs with aminoterminal topogenic sequences and an overall preference of 92% for aminoterminal topogenic sequences, i.e., also similar to SR and Sec61.

The Sec63 clients included 16.4% precursors of single-spanning type I MPs, 4.5% precursors of multispanning type I MPs, and 52.2% precursors of MPs with TMH (Figure 8H and Table S7) [98]. Thus, 73.1% of the Sec63 clients were precursors of MPs. The precursors with TMH included 19.4% single-spanning MPs (11.9% known type II), 32.3% multispanning MPs (25.4% type II and 4.5% type III), and 1.5% TA proteins (Figure 8H, Tables S5, S7, and S8). The hydropathy of the single TA was 2.152. When the multispanning precursors with TMH were analyzed for further details, the numbers for type II MPs were 7.5% below four TMDs and 17.9% above four TMDs, and 10.4% had large ER lumenal domains and a correspondingly high proportion of N-gylcosylation (47%); the numbers for the type III MPs were 4.5% above four TMDs. Thus, compared to Sec62, Sec63 showed a less pronounced preference for amino-terminal topogenic sequences and a general preference for multispanning type II MPs with typically more than four TMDs and a significant proportion of large ER lumenal domains (more than 41%).

In the previous analysis of TMHs for their positioning within the precursor proteins revealed that 69% of Sec63 clients had comparatively amino-terminal TMHs (Figure 9H and Table S8) [202]. Considering type I MPs, Sec63 showed a 78% preference for MPs with aminoterminal topogenic sequences and a general preference for aminoterminal topogenic sequences of 84%.

#### 2.4.3. The ER Membrane Complex or EMC

The decameric EMC comprises an Oxa1-related MP insertase (EMC3) [28,176,185]. As concluded by several groups from proteomic analyses of yeast as well as human cells after knock-out of EMC subunits, TA-MPs are some of their clients, but most of them are multispanning MPs that are cotranslationally inserted into the ER membrane in cooperation with the Sec61 channel [176,177,178,179,180,181,182,183,184,185]. Remarkably, EMC involvement has also been observed in the human system for some single-spanning type III MPs as well as some HP proteins [40,184]. Recently, a model was proposed in which EMC can also pass on clients to the MPT and cooperate through direct interaction with the PAT-GEL-BOS supercomplex [190].

According to our analysis of published data from our colleagues, the EMC clients identified after EMC2, EMC4, or EMC6 knock-out in HeLa cells included 18.6% precursors with SP (including no single-spanning MPs, but 3.4% precursors of multispanning MPs) and 81.3% precursors of MPs with TMH (Figure 8J and Table S7) [178,179]. Thus, 84.7% of EMC clients were precursors of MPs. The EMC clients with TMH included 1.7% single-spanning type III MPs, 76.3% multispanning MPs (66.1% known type II and 8% type III MPs), and 3.4% TA proteins (Figure 8J, Tables S5, S7, and S8) [202]. The average hydropathy of the two TAs was 2.0. In the detailed analysis of the multispanning MPs with TMH, the numbers for type II MPs were 3.4% below and 59.3% above four TMDs; 27.1% had large ER lumenal domains and a correspondingly high proportion of N-gylcosylation (49%). The number of type III MPs was 3.4% below and 5.1% above four TMDs. Thus, the EMC showed a general preference for clients with amino-terminal topogenic sequences and a strong overall preference for multispanning type II MPs with more than four TMDs and, in almost half the cases (41%), large ER lumenal domains.

The previous analysis of the TMHs for their positioning within the precursor proteins revealed that 73% of EMC clients had comparatively amino-terminal TMHs, i.e., similar to SR and Sec61 (Figure 9J and Table S8) [202]. Considering type I MPs, the EMC showed a 74% preference for MPs with aminoterminal topogenic sequences and an overall preference for aminoterminal topogenic sequences of 78%, i.e., also similar to SR and Sec61.

### 2.5. N-Terminal Methionine Excision and Acetylation of Membrane Protein Precursors

The heterodimeric chaperone NAC coordinates the cotranslational N-terminal methionine excision and N-terminal acetylation of about 80% of all nascent polypeptides, including the MPs (Figure 4) [194]. The abundant cytosolic NAC, in contrast to the less abundant SRP, is recruited to the ribosome during translation initiation and then ideally positioned at the ribosomal tunnel exit to scan each nascent polypeptide chain as it emerges from the tunnel (Figure 4). In the absence of an N-terminal SP, NAC recruits a METAP and subsequently an N-acetyltransferase (Nat) to facilitate the two covalent modifications, N-terminal methionine excision and N-terminal acetylation. SRP antagonizes NAC binding in the case of SP-containing precursors and thus cotranslational N-terminal methionine excision and N-terminal acetylation. However, many TMHs of MP precursors are not N-terminal, raising the question of whether these proteins can undergo these two covalent modifications. Therefore, clients of components involved in the biogenesis of MPs at the human ER were screened for N-terminal methionine excision and N-acetylation in the NCBI protein database (https://www.ncbi.nlm.nih.gov/protein (accessed on 1 March 2025)). The results are shown in Figure 9 (Tables S6 and S8).

The results clearly demonstrate that the two cotranslational N-terminal modifications are compatible with ER targeting and membrane insertion of MP clients for almost all analyzed components in human cells, with the exception of TRAM1 with a comparatively low number of putative MP clients (Figure 9, Tables S6 and S8). Starting from a modified EMC client with a TMH in the range from amino acid residues 29 to 49, the critical starting point of the TMH enabling the two modifications is on average 52 amino acid residues, i.e., just downstream of the average SP with a typical length of 15 to 50 amino acid residues [13]. Considering that at any given time of protein synthesis, approximately 40 amino acid residues of a nascent polypeptide chain are buried in the ribosomal polypeptide tunnel, it appears that cotranslational N-terminal methionine excision and N-acetylation of MPs with TMH is possible in human cells, as long as the TMH is not located at the immediate N-terminus.

## 3. Discussion

### 3.1. Unexpected Insights into General Protein Biogenesis at the ER, Revisited

#### 3.1.1. mRNA- and/or Ribosome-Receptors and the TIGER Domain

With respect to mRNA- and/or ribosome-targeting to the human ER, we addressed the two receptors, termed RRBP1 and KTN1, with our proteomic approach and identified putative clients that are directed towards the secretory pathway (Figure S1, Tables S3 and S6) [170]. For RRBP1, a role as an RNC-receptor in the biogenesis of 22 precursors with SP (including 6 single-spanning type I MPs) and 17 precursors with TMH (7 single-spanning and 8 multispanning MPs, as well as 2 HP proteins, ATL2 and ATL3) as well as a genetic interaction with SRA and SRB has been suggested (Tables S3, S6 and S17, Figure 7). For KTN1, the negative effect on the biogenesis of 3 precursors with SP (including the multi-spanning type I MP CD47) and 4 precursors with TMH is consistent with the idea that KTN1 also plays a role in the biogenesis of proteins of the secretory pathway, such as CD47, as is the genetic interaction with RRBP1 (Tables S3, S6 and S17, Figure 7). Furthermore, the negative effect on CD47 suggested a function of KTN1 as a putative ER-resident mRNA receptor in the so-called TIGER domain, which was proposed to form a cytosolic micro-domain enabling the enrichment of MP-encoding mRNAs with multiple AU-rich elements (AREs) in their 3′ UTRs in the ER vicinity [166,167] (Figure S1). The proteomic approach also suggested a function in MP biogenesis at the ER for ERj1 [170,212,213,214,215], which represents another ER MP that presumably interacts with mRNAs or RNCs (Tables S3 and S6). Interestingly, ERj1 shared five of its nine MP clients with TMH, with KTN1 (GALT4, PTPLB, QPCTL, ATP6V0C, BCL2L1). Therefore, it has been proposed that KTN1 represents the mRNA-binding protein localized in the ER membrane and enriched in the TIGER domain to take over mRNAs from the cytosolic RNA-binding protein TIS11B and to initiate their translation at Sec61-associated ribosomes (Figure S1) [170]. When the mRNA encodes an MP precursor with SP (CD47) or with an amino-terminal TMH (GALT4, PTPLB, QPCTL, ATP6V0C), the nascent precursor begins to sample the Sec61 channel. This leads to spontaneous channel opening or the recruitment of Sec61 channel auxiliary factors, such as ERj1. Therefore, we proposed that ERj1 cooperates with KTN1 in allowing Sec61 channel opening when BiP is bound to the J-domain of ERj1 [170]. Subsequently, CD47 is expected to insert into the ER membrane via the lateral gate of the Sec61 channel.

#### 3.1.2. PEX3 and ER Exit Sites (ERES)

With respect to targeting precursor polypeptides to the human ER, the results of classical in vitro studies for ER protein import were confirmed in our experimental approach. This means that all four known targeting pathways were able to target SPs and TMHs to the Sec61 complex in the ER membrane. The PEX3/PEX19-dependent pathway plays an important role in targeting peroxisomal MPs and certain hairpin MPs of the ER and lipid droplets to an ill-defined ER subdomain [7,8,37,38]. The putative clients found in PEX3 knock-down or knock-out cells included seven peroxisomal MPs and two ER HP proteins (ATL1, RTN3). This confirms the two previously identified classes of PEX19/PEX3 clients for ER targeting in human cells (Table S6) [157]. In addition, 28 proteins with SP (including 14 collagens plus collagen-related proteins) were identified as putative PEX3 clients (Table S6). In analogy to our suggestion related to KTN1 and its putative presence in the TIGER domain, the latter findings led us to propose that the subdomain involved in the budding of peroxisomal precursor vesicles and the pinching off of lipid droplets might be physically or even spatially related to ER exit sites for large cargo vesicles critical for collagen secretion [157]. This suggestion was recently confirmed in *Drosophila* [216]. Furthermore, we hypothesized that defects in the biogenesis of certain collagens might contribute to the devastating effects of PEX3 deficiency in Zellweger patients [157].

### 3.2. Unexpected Results Regarding Membrane Protein Biogenesis at the ER Membrane

#### 3.2.1. Membrane Protein Insertases in the ER Membrane: How Many Are There?

Since the demands on the TMHs of single-spanning type III MPs and TA proteins are similar in hydrophobicity and allow hydrophilic add-on, and only the N- versus C-terminal orientation of the polypeptide chains is different, these types of precursors and, possibly, even multispanning type III MPs could be inserted into the ER membrane by the same insertases (Figure 2). This speculation will obviously need to be addressed in future work using the purified and reconstituted components in classical in vitro assays.

Among the results of our proteomic approach was the unexpected finding of multispanning MPs as Wrb/Caml clients [156]. This supported a more general targeting function of the TRC/GET pathway in targeting or possibly even membrane insertion, which had previously been suggested based on classical in vitro studies and in the context of related human diseases (Table S1) [134,149,151]. This view is supported by the observations that the mitochondrial Oxa1 insertases, Oxa1 and Oxa2, can insert multispanning MPs and that an artificial EMC3/EMC6-hybrid can replace Oxa1 in mitochondria [31,185].

The Snd2/TMEM109 appears to enable targeting or maybe even membrane insertion for certain MPs, such as TA proteins, as well as multispanning MPs with negligible ER lumenal domains and comparatively negative ΔG for TMH [156,202]. The latter could involve a putative coiled-coil domain within the cytosolic carboxy-terminal domain, as well as a putative hydrophilic vestibule near the membrane surface of Snd2, reminiscent of the insertases of the Oxa1 superfamily [156]. It is tempting to imagine the respective clients, such as Sec62, as bitopic HP-like proteins [217,218], which is consistent with the observation that a monotopic HP protein was found among the clients of the SND pathway [135]. With respect to this highly speculative insertase activity of Snd2/TMEM109, at least for TA clients, the ΔG values were more negative for Snd2 clients as compared to Wrb clients [202]. Obviously, this speculation must also be interrogated in future work using the purified and reconstituted components in classical in vitro assays.

Of note, in a recent preprint, *Chaetomium thermophilum* Snd3 was indeed structurally characterized as an MP insertase with a membrane-embedded hydrophilic groove reminiscent of, but distinct from, Oxa1, and as part of a putative fungal multipass translocon together with Sec61 complex, CCDC47, and TRAPα [219]. However, combined with the reported lack of sequence homology between human TMEM109 and yeast Snd3 [156], this also excluded human TMEM109 as a Snd3 homolog. It remains to be seen whether the human Snd2/TMEM109 heterodimer is capable of forming another MP insertase or whether it only acts in targeting, as originally proposed [154,155].

As noted above, recent work has proposed Snd2 as a player in the SRP/SR pathway rather than a component of SRP-independent ER protein import [65]. This apparent conflict was addressed here by revisiting the original proteomic data [156]. According to the recent proposal, Snd2 depletion should result in a negative effect on similar types of precursor polypeptides as compared to SR depletion, and might even show an overlap in negatively affected precursors, as we had observed for Sec62 and Sec63 [98]. However, neither of these was the case, regardless of whether SR clients were compared with Snd2 clients after Snd2 depletion or Snd2/Wrb double depletion (Figure 8, Figure 9, and Figure S9). Furthermore, there clearly is a need for SRP-independent ER protein import since human cells are viable in the absence of SRP or SR [172]. Nevertheless, I see no reason why Snd2 should not be involved in SRP-dependent as well as SRP-independent ER protein import.

Based on both its proposed ceramide and sphingolipid binding site as well as its putative MP clients [201], TRAM1 might function as an MP insertase for single- and multispanning type II MPs with weakly hydrophobic TMHs and with negligible ER lumenal domains. This could occur by providing a lipid-filled cavity analogous to MPT or by local membrane thinning or even lipid scrambling, as has been proposed for TRAP [220,221,222,223,224,225,226,227,228]. Following this highly speculative insertase activity of TRAM1, at least for TA proteins, the ΔG values of TRAM1 clients were striking, as they exhibited the lowest observed hydrophobicity values of all TA proteins [202].

#### 3.2.2. GPI-Anchored MPs

The SND pathway can act posttranslationally and therefore accept GPI-anchored MPs as clients, at least sometimes in concert with Sec62 and possibly when the GPI-attachment sequence acts as a membrane targeting sequence [173,175]. We also made observations related to GPI-anchored MPs, although only about 130 of these are known in human cells [173]. Our proteomic approach identified the GPI-anchored MPs with SP, CD59, and CD109, as clients of RRBP1 (CD109) and Sec63 (CD59), respectively, and the putative GPI-anchored and TMH-containing BST2 as a client of SR, Sec61, Sec62, and Sec63 [98,156,170,200].

#### 3.2.3. Examples for Client Pathways or System Redundancy

Comparative analysis of quantitative MS data and subsequent differential protein abundance analyses characterized the topogenic sequences of MP clients of four membrane-targeting and four established membrane insertion components for the human ER. As expected, MP precursors with cleavable amino-terminal SPs or TMHs were proven to be the predominant clients of the SRP and the Sec61 complex, while precursors with more central or even carboxy-terminal TMHs dominated the client spectra of the SND- and TRC/GET-pathways for membrane targeting in living human cells. Furthermore, it was confirmed that the MP insertase EMC exhibits a preference for multispanning MPs with weakly hydrophobic TMHs and possesses TA protein clients with less hydrophobic TAs as compared to the Wrb/Caml insertase.

Some MPs were found to be negatively affected in multiple depletion experiments and were therefore characterized as putative clients of various targeting and membrane insertion components. Examples include (i) the single-spanning type II MP ERLIN2, which was identified as a substrate of SR, Sec61, Sec62, and TRAM1; (ii) the single-spanning type II and/or GPI-anchored BST2, which involved SR, Sec61, Sec62, and Sec63; (iii) the multispanning, type II MP SOAT1, which was characterized as a putative client of RRBP1, Snd2, Wrb, ERj1, TRAP, and EMC; and (iv) the TA FAR1, which appeared upon depletion of Wrb, PEX3, and Sec61.

These observations provided insights into possible biosynthetic pathways of these MPs in living cells: FAR1 with a hydrophobicity score of 1.589, appears to be targeted and membrane inserted by the TRC/Wrb system, or alternatively, targeted to the Sec61 complex by the PEX19/PEX3 or the TRC system and inserted into the ER membrane by Sec61; BST2 is annotated as single-spanning type II MP as well as GPI-anchored MP and appears to involve a classical biosynthetic pathway including SR for ER targeting and Sec61, Sec62, and Sec63 for membrane insertion, thus confirming the previously reported cotranslational role of Sec62 and Sec63 [106]; a similar conclusion can be drawn for ERLIN2, except that TRAM1 also plays a role; in the case of SOAT1 the picture is most complex: RRBP1 (and possibly also ERj1) provides a first level, namely an mRNA- or ribosome-targeting step, Snd2 or Wrb act in protein targeting, and the multipass-TRAP translocon in collaboration with EMC facilitates membrane insertion (see below). The latter, in particular, raises the question of what features of SOAT1 determine this complex scheme. The eleven ATTA motifs in the 3′UTR of the SOAT1 mRNA could be crucial for the first targeting step, while the non-aminoterminal and almost central TMH could be crucial for the second targeting step and, together with the nine TMDs, also for the choice of EMC plus the multipass-TRAP translocon (see below) [170].

#### 3.2.4. Putative Regulatory Mechanisms

As previously proposed [202], specific client features may enable differential regulation of ER protein import under different cellular conditions through reversible phosphorylation and/or Ca^2+^ binding of ER protein import components, such as Sec62/Sec63 and TRAPα, favoring certain clients under certain cellular conditions, such as stress [229,230,231,232].

Another recently described regulatory mechanism involves ribosome-associated cytosolic proteins such as Lsg1. Lsg1 apparently tethers 60S ribosomal subunits to the ER and regulates the initiation of synthesis of select proteins, such as MPs, at the ER surface. This is reminiscent of the presence of 60S ribosomal subunits at the ER and the role of mRNA, 60S, and ribosome receptors, discussed above (Section 3.1.1.) [164,233].

#### 3.2.5. EMC Clients

**Figure 10:**
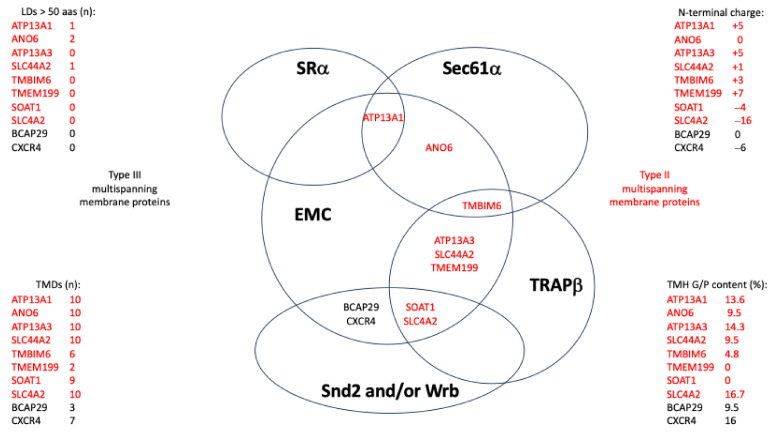
EMC client overlaps and client characteristics. See text for details.

In a previous analysis, we observed a low client overlap for the EMC and Sec61 complex [18]. In these experiments, eight precursors were found that showed dependence on both complexes: five with SP and three with TMH. No MPs were found among the precursors with SP. The three precursors with TMH were multispanning MPs (ANO6, ATP13A1, TMBIM6) with relatively amino-terminal TMHs, an N_in_ or type II membrane topology, and, in two of the cases, with large ER lumenal domains (Figure 10). In addition, we discovered an overlap of six multispanning type II MPs between EMC and TRAP (ATP13A3, SLC4A2, SLC44A2, SOAT1, TMBIM6, TMEM199). This suggests that not only the Sec61 channel/translocon cooperates with EMC in the biogenesis of some MPs, but the TRAP- or even the OSTA translocon. This is consistent with the fact that several of the EMC clients are N-glycoproteins. Notably, ANO6 was also found among the SR clients, and SLC4A2 and SOAT1 were characterized as Snd2 as well as Wrb clients (Figure 10, Tables S6 and S8). Interestingly, we also identified two multispanning type III or N_out_ MPs (BCAP29, CXCR4) as EMC and Snd2, as well as Wrb clients.

**Figure 11:**
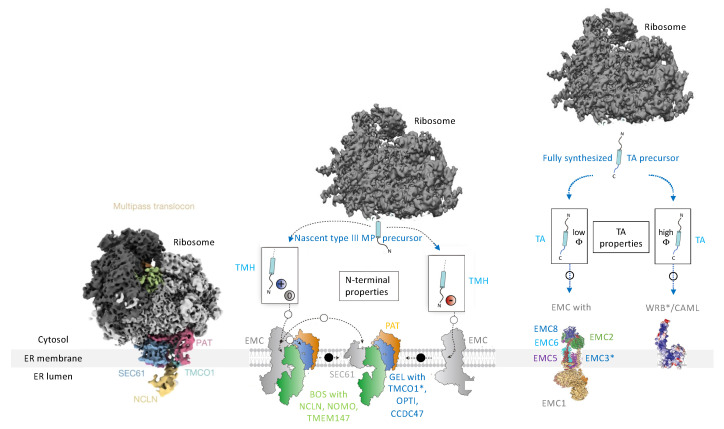
Model for the cooperations of membrane protein insertases in the ER membrane. Notably, BOS binding to EMC and Sec61 is mutually exclusive; BOS binding to the TRAP translocon results in the multipass-TRAP translocon (Figure 1D). The asterisks (*) highlight the Oxa1 superfamily MP insertases. The Figure layout was inspired by reference [190]. See text for details.

In her recent work, R. Voorhees interrogated the functional interactions of EMC and showed that the PAT-GEL-BOS supercomplex not only associates and cooperates with the Sec61 complex to form the MPT (Figure 11, left part), but also associates and cooperates with EMC in the membrane insertion of various client proteins (Figure 11, central part) [190]. Furthermore, it has long been known that EMC can hand over certain client proteins to the Sec61 complex. It was concluded that multispanning type III MPs are initially targeted to the EMC, which, depending on the properties of their aminoterminal domain, either inserts the TMH (first TMD of the MP) directly into the ER membrane or associates with the PAT-GEL-BOS supercomplex via interaction with BOS. This cooperatively facilitates membrane insertion of the first TMD at the EMC or hands the client over to the GEL or Sec61 complex for membrane insertion of the following TMDs by the MPT or -as proposed based on our results, to the multipass-TRAP translocon. The latter has also been proposed for clients initially inserted into the ER membrane by EMC [190]. Thus, it appears that there are multiple pathways by which MPs achieve their correct orientation in the ER membrane, and that membrane insertion pathways are at least as complex and possibly redundant as membrane targeting pathways. Regarding targeting pathways, our results indicate that BCAP29 and CXCR4 are clients of Snd2 and Wrb. Therefore, targeting does not necessarily occur via SRP and SR, even cotranslational targeting that involves SGTA and Bag6 complex, reminiscent of TA MPs (Figure 11, right part).

As noted above, the identification of putative EMC substrates with SP was dismissed in previous work as a possible indirect effect. However, given these recent findings, I see no reason to ignore the possibility that EMC can also hand over SP-containing precursors of type I MPs or even soluble proteins to the Sec61 channel for complete ER import.

### 3.3. Medical Aspects

The proteomic approach also led to some interesting suggestions with potential medical relevance, such as links between PEX3, which is associated with the Zellweger syndrome, and the biogenesis of various collagens, as well as between a subclass of Congenital Disorders of Glycosylation (CDGs) and the role of TRAP in the biogenesis of many glycoproteins [7,149,151]. In the first case, we suggested that defects in the biogenesis of certain collagens could contribute to the devastating effects of PEX3 deficiency in Zellweger patients [157]. The second case highlighted that CDGs do not necessarily result from defects in components directly involved in N-glycosylation or GPI-attachment, but can also be caused by deficiencies in ER targeting and import components that act upstream of these covalent modifications (Table S1) [139,200]. The latter was reiterated by the recent observation that deficits in the TRC/GET pathway can also cause CDGs [149,151].

The expectation is that a complete picture of substrate specificities of components that enable membrane-targeting and insertion of MP precursors, putative pathway overlaps, and regulatory mechanisms will eventually also define potential human drug targets that are involved in the biogenesis of certain MP types. Recently, there was progress with small molecule inhibitors in this direction with respect to the central Sec61 complex [47,234,235,236,237,238,239,240,241,242,243]. In the future, this avenue may be even more promising for less central auxiliary components or MP insertases [47,244].

### 3.4. Novel Aspects: N-Terminal Methionine Excision and N-Acetylation of Membrane Protein Precursors

An estimated 80% of all cellular proteins are enzymatically modified by N-acetylation, including MPs [194,196,197,245,246]. However, some of the latter are thought to be posttranslationally N-terminally acetylated in the Golgi apparatus [199]. In short, NAC binds first to the nascent chains during polypeptide synthesis as they emerge from the ribosomal tunnel exit. It then recruits one of the two METAPs and subsequently one of several NATs. Substrate specificity is thought to be determined by the amino acid residue following the initiating methionine (Figure 4) [194]. SRP can antagonize NAC binding in the case of SP-containing precursor polypeptides of secretory and membrane proteins, thus inhibiting cotranslational N-terminal methionine excision and N-terminal acetylation.

The interpretation of the present analysis of our proteomic data is that cotranslational N-terminal methionine excision and N-acetylation of MPs with TMH is possible in human cells, as long as the TMH is not located directly at the N-terminus (Figure 9, Tables S6 and S8). These two modifications were observed in clients of almost all analyzed components, regardless of whether the components are considered to act co- or posttranslationally. The critical starting point of the TMH that allows the two modifications appears to be at least approximately 30 amino acid residues and on average 52 amino acid residues downstream of the N-terminus, i.e., the hydrophobicity of the nascent polypeptide chain antagonizes binding of the cytosolic chaperone NAC only up to this point in polypeptide chain elongation [194,197]. In general, N-acetylation of precursor polypeptides does not appear to inhibit ER protein targeting and membrane insertion in human cells as long as the TMH is not located at the immediate N-terminus [195].

### 3.5. Limitations and Critical Review of the Experimental Approach

#### 3.5.1. Limitations of the Experimental Approach

The results that are summarized in Figure 6 and Figure 7 and Tables S3–S8 indicate weaknesses and limitations of the experimental approach. In addition, there were no small presecretory proteins, as well as small MPs and hardly any GPI-anchored MPs, detected by the approach. However, it should be noted that only approximately 130 GPI-anchored MPs are known in human cells [173]. For comparison, the numbers are similar for small precursors, TA proteins of the secretory pathway, and even lower for HP proteins [38,39,92]. Therefore, the detection of a small number of representatives of a particular protein class among the substrates of a certain component was not discussed here in any detail.

As stated above, the interpretation of the proteomic data was weakest where the total number of negatively affected MPs of the secretory pathway, and thus of putative MP clients, was lowest. This was the case of KTN1 and ERj1 depletion, with a number of 9 and 11 putative MP clients, respectively (Tables S3–S5). This problem originally also occurred after transient depletion of PEX3, Sec62, and Sec63 and was the reason for the use of CRISPR/Cas9-mediated Sec62- as well as Sec63-knock-out cells or fibroblasts from PEX3-deficient Zellweger Syndrome patients [98,157]. The additional proteomic data solved the problem for PEX3, Sec62, and Sec63.

Typically, 30% of the proteome of a human cell comprises clients of ER protein import. However, even in the case of Sec61 depletion, only 197 proteins with SP plus 98 with TMH, i.e., about 300 of the 6000 quantified proteins or 5%, were negatively affected by the depletion [200]. This raises the question of why we saw only the tips of the icebergs, so to speak. We envision several responsible factors under conditions of siRNA-mediated knockdown: The depletion efficiency and its duration, which were optimized for minimal effects on cell growth and viability, were not high enough to cause significant accumulation and degradation of the respective clients. The MS data suggested a depletion efficiency of approximately 90% for the respective component. The residual amount of the component may have been sufficient to sustain the physiological functions of depleted proteins over the duration of the experiment. A certain function in ER protein import in human cells is compensated for by other components or pathways. Except for the Sec61 complex, we expected that to be the case. Some client proteins may have remained largely unaffected due to either longer half-lives than the respective component or higher than average affinities for the component. Furthermore, some accumulating precursors may have stayed soluble in the cytosol, aggregated, or ended up in mitochondria, where they were out of reach for degradation by the ubiquitin/proteasome system. Notably, we have observed mistargeting of certain precursors of secretory proteins into mitochondria in the absence of Sec61 function in human cells [247]. Therefore, we propose that all these factors together may have contributed, possibly to a different extent for different precursors.

As illustrated in Figure 7, another potential weakness of our experimental approach is that it does not, per se, distinguish between clients and interaction partners of ER protein import components. Therefore, it should ideally be complemented by proximity labeling and/or proximity-specific ribosome profiling, such as described for EMC or the alternative Sec61 complex in yeast [63,76,168,180,248].

Here, this potential problem was addressed for TRAP by re-evaluating the original data (Figure 1 and Table S1) [200]. The key observation of our first study, in which we investigated client specificity using transient component depletion, quantitative MS, and differential protein abundance analysis, was that TRAP supports ER import of SP-bearing precursors, including type I MPs, with high GP-content and therefore low alpha-helical propensity in their SPs [200]. The data reported an average of 18.4% GP content for SPs of TRAP clients and defined a 15% GP content as a threshold for TRAP-dependence of SPs. In addition, they suggested a higher than average GP content of 9.2% for TMHs of TRAP clients. However, at the time, we classified the precursors of the type I MPs and OST subunits DDOST, MAGT1, TUSC3, as well as MPT subunits NCLN and NOMO2, as TRAP clients and included them in the calculation of the average GP-content of SPs of TRAP clients. Furthermore, the TMH-containing precursors of the MPs and OST subunits DAD1, Stt3b, the SPC subunit SPCS2, and TRAM1 were defined as TRAP clients. Although MAGT1 and TUSC3 are subunits of OST-B and, therefore, unlikely TRAP interaction partners, we calculated the GP-contents of SPs and TMHs of TRAP interaction partners and determined an average GP content of 17.6% for SPs and 9.6% for TMHs of the five and four TRAP interaction partners, respectively. Revisiting the client properties thus confirmed the nine TRAP interaction partners as putative clients and, more importantly, the original conclusion with respect to G/P content of SPs and TMHs for TRAP clients [200]. This interpretation is consistent with the facts that the log2 fold change and thus depletion efficiency of the TRAP subunits were more pronounced as compared to the aforementioned OST subunits, and that no significant OST depletion was detected by differential image analysis after cryoelectron tomography of human cells in which TRAP had been depleted using the same siRNAs as in the MS experiments (Figure S8).

An undisputable limitation of the present summary of global analyses of MP biogenesis at the ER concerns the Sec61 complex. Since this complex contributes to MP biogenesis in two ways: it functions as an MP insertase and provides a ribosome binding site to the MP insertase that is termed PAT-GEL-BOS supercomplex, Sec61 clients cannot be distinguished between true clients of the Sec61 channel and clients of the heterotrimeric supercomplex within the MPT, i.e., the ribosome binding site of Sec61 (Figure 3). This puzzle will have to be resolved in future research.

#### 3.5.2. Critical Review of the Experimental Approach

As stated above, the interpretation of the proteomic data was weakest where the total number of putative MP clients was lowest, i.e., the case of KTN1 and ERj1 depletion. As also stated above, this problem originally also occurred after transient depletion of Sec62 and Sec63 and was the reason for the use of CRISPR/Cas9-mediated Sec62- as well as Sec63-knock-out cells [98]. Indeed, the additional proteomic data solved the problem for Sec62 and Sec63.

Therefore, the question arises whether CRISPR/Cas9-mediated knock-out cells would not have been the better choice to begin with. Indeed, the EMC clients were identified in this kind of cells, where the half-lives of clients should not matter [178,179,190]. To address this question here, the positively affected proteins after transient knock-down of Sec62 or Sec63 and after knock-out were re-interrogated (Table S17). Although the number of negatively affected proteins were higher in the Sec62 knock-out versus knock-down cells, as had to be expected, both the total numbers of positively affected proteins as well as the number of proteins that are related to the ubiquitin/proteasome system were lower in the Sec62 knock-out (13 and 5, respectively) versus knock-down cells (25 and 14, respectively). The Sec63-depleted cells are not considered here, since the numbers were low for both cell types and the knock-out cells were mixed guide RNA cells, i.e., not complete knock-out cells [98]. Thus, the comparison between knock-down and knock-out relies on a single example and is further complicated by the fact that in our experiments, the knock-out cells were derived from HEK293 cells, and the knock-down cells were HeLa cells. Nevertheless, the impression from this single comparison is that in the knock-out cells, different adaptation processes must have occurred as compared to the knock-down cells. It appears that adaptation in the latter occurred at the level of the clients via increased chaperone and ubiquitin/proteasome activity and/or capacity. In contrast, knock-out cells may have adapted by globally lowering the expression of genes or mRNAs that code for clients. Notably, we originally refrained from using knock-out cells because we wanted to compare all depleted components with the essential Sec61α1 protein.

## 4. Materials and Methods

The methodological details, except for the siRNAs used in the BiP and Snd2 plus Wrb depletions that are reported here (Table S18, Figures S4 and S6), were described previously [18,98,156,157,170,200,201,202], and the original MS data were deposited at the ProteomeXchange Consortium (http://www.proteomexchange.org (accessed on 9 August 2025)) with the data set identifiers that are given in Table S2. Furthermore, the original results [98,156,157,170,200,201] as well as the first comparative analyses have been published previously [18,202]. All statistical analyses were performed using the SAM R-package (http://www-stat.class.stanford.edu (notably, the original link is not accessible anymore but the tool is still available on the internet)) [200] and are summarized here in Tables S19 and S20. As also described previously in detail, characterization of putative client genes was screened for AU-rich elements (http://rna.tbi.univie.ac.at/AREsite2/welcome (accessed on 2 May 2021)) [170], and topogenic sequences (SP, TMH) were analyzed for grand average of hydropathy (https://www.bioinformatics.org/sms2/protein_gravy.html (accessed on 12 May 2025)), GP-content, and apparent ΔG for membrane insertion (http://dgpred.cbr.su.se (accessed on 12 May 2025)) [200]. Type II and III membrane topologies and the numbers of ER lumenal domains with a content of >50 amino acid residues were determined (https://dtu.biolib.com/DeepTMHMM/ (accessed on 12 May 2025)) [202]. Putative MP clients were also screened for intrinsically disordered domains as well as N-terminal methionine excision and N-acetylation in the NCBI protein database (https://www.ncbi.nlm.nih.gov/protein (accessed on 1 March 2025)). The number of TMDs in MP precursors was determined in our proteomic data sets for the first time here. The N-terminal methionine excision and N-acetylation of MP precursors were determined in our proteomic data sets for the first time here.

## 5. Conclusions

In nucleated human cells, the biogenesis of most membrane proteins (MPs) is initiated at the ER membrane. This process involves decisive oligopeptides in the precursor polypeptides, such as N-terminal signal peptides (SPs) or not-necessarily N-terminal transmembrane helices (TMHs). In addition, more than one hundred cytosolic and ER proteins facilitate membrane-targeting and -insertion, folding, or covalent modification of MPs. Four targeting pathways are known to direct precursor polypeptides to the central Sec61 complex and its various allosteric effectors in the ER membrane, or to one of the three Sec61-cooperating or stand-alone MP insertases.

We and others combined the depletion of components of most of these pathways with label-free quantitative mass spectrometric analysis of the total proteome of depleted and non-depleted cells. This was followed by differential protein abundance analysis to identify clients of components involved in ER targeting or membrane insertion of precursor polypeptides, and their rules of engagement. The results from this in vivo-like approach confirmed and further consolidated many of the lessons from the classical in vitro experiments, but also added new ones, such as that not only the polarity or hydrophobicity of the SP or TMH matters, and that not only what is up-front, i.e., at the N-terminus, that counts. In addition, the glycine and proline content, i.e., the helix propensity of SP or TMH, as well as clusters of charged amino acid residues downstream of SP, play a role in substrate specificity and affinity.

Further interesting new observations are that cotranslational N-terminal methionine excision and N-acetylation of MPs with TMH is possible in human cells, provided the TMH is located an average of 52 amino acid residues downstream of the N-terminus. Furthermore, N-acetylation of MP precursors does not appear to inhibit MP biogenesis unless the TMH is located directly at the N-terminus.

Finally, the proteomic approach led to some interesting insights with potential medical relevance, for example, a link between PEX3, associated with Zellweger syndrome, and the biogenesis of various collagens. Since sixty percent of human drug targets are MPs, the expectation is that a complete picture of their biogenesis will define novel drug targets.

## Data Availability

The original MS data (.raw and .txt files) have been deposited to the ProteomeXchange Consortium via the PRIDE partner repository with the indicated data set identifiers (http://www.proteomexchange.org (accessed on 9 August 2025)) as given in Table S2. Any additional information required to analyze the data reported in this paper is available from the corresponding author upon request.

## Supplementary Materials

The following supporting information can be downloaded at: https://www.mdpi.com/article/10.3390/ijms26188823/s1.

## Abbreviations

The following abbreviations are used in this manuscript:

BiP	Immunoglobulin heavy chain binding protein, an HSP70 protein family member
BOS	Back of Sec61 complex
CAML	Calcium modulating ligand
EMC	ER membrane complex, comprising an Oxa1 superfamily membrane protein insertase
ER	Endoplasmic reticulum
ERj	ER J-domain protein, an HSP40 protein family member
GEL	GET- and EMC-like, comprising an Oxa1 superfamily membrane protein insertase
GET	Guided entry of TA proteins
GO	Gene ontology
GPI	Glycosylphosphatidylinositol
GPI-T	GPI-transamidase
HP	Hairpin
HSP	Heat-shock protein
KTN	Kinectin
METAP	Methionine aminopeptidase
MP	Membrane protein
NAC	Nascent polypeptide-associated complex
MPT	Multipass translocon
NAT	N-acetyltransferase
OST	Oligosaccharyl transferase
PAT	PAT 10 comprising complex
PDI	Protein disulfide isomerase
PEX	Peroxisomal protein
PPI	Peptidyl-prolyl cis/trans isomerase
RNC	Ribosome-nascent chain
SEC	Protein involved in secretion
SND	SRP-independent
SP	Signal peptide
SPC	Signal peptidase complex
SR	SRP-receptor
SRP	Signal recognition particle
TA	Tail anchor
TMD	Transmembrane domain
TMEM	Transmembrane protein
TMH	Most N-terminal transmembrane helix
TRAM	Translocating chain-associated membrane protein
TRAP	Translocon-associated protein
TRC	Transmembrane recognition complex
WRB	Tryptophan-rich basic protein, an Oxa1 superfamily membrane protein insertase

## References

[1] Palade G. (1975). Intracellular aspects of protein synthesis. Science.

[2] Palade G., Porter K.R. (1954). Studies on the endoplasmic reticulum. J. Exp. Med..

[3] Blobel G. (1980). Intracellular protein topogenesis. Proc. Natl. Acad. Sci. USA.

[4] Schekman R., Novick P. (2004). 23 genes, 23 years later. Cell.

[5] Egea P.F., Stroud R.M., Walter P. (2005). Targeting proteins to membranes: Structure of the signal recognition particle. Curr. Opin. Struct. Biol..

[6] Aviram N., Schuldiner M. (2017). Targeting and translocation of proteins to the endoplasmic reticulum at a glance. J. Cell Sci..

[7] Jansen R.L.M., Van der Klei I.J. (2019). The peroxisome biogenesis factors Pex3 and Pex19: Multitasking proteins with disputed functions. FEBS Lett..

[8] Dhiman R., Caesar S., Thiam A.R., Schrul B. (2020). Mechanisms of protein targeting to lipid droplets: A unified cell biological and biophysical perspective. Semin. Cell Dev. Biol..

[9] Hansen K.G., Aviram N., Laborenz J., Bibi C., Meyer M., Spang A., Schuldiner M., Herrmann J.M. (2018). An ER surface retrieval pathway safeguaerds the import of mitochondrial membrane proteins in yeast. Science.

[10] Koch C., Schuldiner M., Herrmann J.M. (2021). ER-SURF: Riding the endoplasmic reticulum SURFace to mitochondria. Int. J. Mol. Sci..

[11] Blobel G., Dobberstein B. (1975). Transfer of proteins across membranes: I. Presence of proteolytically processed and unprocessed nascent immunoglobulin light chains on membrane-bound ribosomes of murine myeloma. J. Cell Biol..

[12] Blobel G., Dobberstein B. (1975). Transfer of proteins across membranes: II. Reconstitution of functional rough microsomes from heterologous components. J. Cell Biol..

[13] von Heijne G. (1985). Signal sequences. J. Mol. Biol..

[14] Ng D.T., Brown J.D., Walter P. (1996). Signal sequences specify the targeting route to the endoplasmic reticulum membrane. J. Cell Biol..

[15] Hegde R.S., Bernstein H. (2006). The surprising complexity of signal sequences. Trends Biochem. Sci..

[16] Goder V., Spiess M. (2003). Molecular mechanism of signal sequence orientation in the endoplasmic reticulum. EMBO J..

[17] Teufel F., Armenteros J.J.A., Johansen A.R., Gislason M.K., Pihl S.I., Tsiigos K.D., Winther O., Brunak S., Von Heijne G., Nielsen H. (2022). Signal 6.0 predicts all five types of signal peptides using protein language models. Nat. Biotechnol..

[18] Lang S., Nguyen D., Bhadra P., Jung M., Helms V., Zimmermann R. (2022). Signal peptide features determining the substrate specificities of targeting and translocation components in human ER protein import. Front. Physiol..

[19] Chen X., Van Valkenburgh C., Liang H., Fang H., Green N. (2001). Signal peptidase and oligosaccharyltransferase interact in a sequential and dependent manner within the endoplasmic reticulum. J. Biol. Chem..

[20] Liaci A.M., Steigenberger B., Tamara S., de Souza P.T., Gröllers-Mulderij M., Ogrissek P., Marrink S.-J., Scheltema R.A., Förster F. (2021). Structure of the human signal peptidase complex reveals the determinants for signal peptide cleavage. Mol. Cell.

[21] Braunger K., Pfeffer S., Shrimal S., Gilmore R., Berninhausen O., Mandon E.C., Becker T., Förster F., Beckmann R. (2018). Structural basis for coupling protein transport and N-glycosylation at the mammalian endoplasmic reticulum. Science.

[22] Wild R., Kowal J., Eyring J., Ngwa E.M., Aebi M., Locher K.P. (2018). Structure of the yeast oligosaccharyltransferase complex gives insight into eukaryotic N-glycosylation. Science.

[23] Neupert W. (2012). A mitochondrial odyssey. Annu. Rev. Biochem..

[24] Schatz G. (2012). The fires of life. Annu. Rev. Biochem..

[25] Busch J.D., Fielden L.F., Pfanner N., Wiedemann N. (2023). Mitochondrial protein transport: Versatility of translocases and mechanisms. Mol. Cell.

[26] von Heijne G., Gavel Y. (1988). Topogenic signals in integral membrane proteins. Eur. J. Biochem..

[27] Baker J.A., Wong W.-C., Eisenhaber B., Warwicker J., Eisenhaber F. (2017). Charged residues next to transmembrane regions revisited: “Positive-inside rule” is complemented by the “negative inside depletion/outside enrichment rule”. BMC Biol..

[28] Hegde R.S., Keenan R.J. (2022). The mechanism of integral membrane protein biogenesis. Nat. Rev. Mol. Cell Biol..

[29] O’Keefe S., Pool M.R., High S. (2022). Membrane protein biogenesis at the ER: The highways and byways. FEBS J..

[30] Rapoport T.A. (2023). A life of translocations. Annu. Rev. Biochem..

[31] Kizmaz B., Herrmann J.M. (2023). Membrane insertases at a glance. J. Cell Sci..

[32] Chen J., Zhou X., Yang Y., Li L. (2025). Protein translocation through α-helical channels and insertases. Structure.

[33] Kutay U., Hartmann E., Rapoport T.A. (1993). A class of membrane proteins with a C-terminal anchor. Trends Cell Biol..

[34] Schuldiner M., Metz J., Schmid V., Denic V., Rakwalska M., Schmitt H.D., Schwappach B., Weissman J.S. (2008). The GET complex mediates insertion of tail-anchored proteins into the ER membrane. Cell.

[35] Rabu C., Schmid V., Schwappach B., High S. (2009). Biogenesis of tail-anchored proteins: The beginning for the end?. J. Cell Sci..

[36] Ast T., Cohen G., Schuldiner M. (2013). A network of cytosolic factors targets SRP-independent proteins to the endoplasmic reticulum. Cell.

[37] Schrul B., Kopito R.R. (2016). Peroxin-dependent targeting of a lipid-droplet-destined membrane protein to ER subdomains. Nat. Cell Biol..

[38] Yamamoto Y., Sakisaka T. (2018). The peroxisome biogenesis factors posttranslationally target reticulon homology-domain containing proteins to the endoplasmic reticulum membrane. Sci. Rep..

[39] Borgese N., Coy-Vergara J., Colombo S.F., Schwappach B. (2019). The ways of tails: The GET pathway and more. Proteins.

[40] Leznicki P., Schneider H.O., Harvey J.V., Shi W.Q., High S. (2021). Co-translational biogenesis of lipid droplet integral membrane proteins. J. Cell Sci..

[41] Kinoshita T. (2020). Biosynthesis and biology of mammalian GPI-anchored proteins. Open Biol..

[42] Sinning I., McDowell M.A. (2022). Cryo-EM insights into tail-anchored membrane protein biogenesis. Curr. Opin. Struct. Biol..

[43] Jung M., Zimmermann R. (2023). Quantitative mass spectrometry characterizes client spectra of components for targeting of membrane proteins to and their insertion into the membrane of the human ER. Int. J. Mol. Sci..

[44] Gemmer M., Chaillet M., Förster F. (2024). Exploring the molecular composition of the multipass translocon in its native membrane environment. Life Sci. Alliance.

[45] Vismpas D., Förster F. (2024). RAMPing up the knowledge of the translocon. eLife.

[46] Lang S., Pfeffer S., Lee P.-H., Cavalié A., Helms V., Förster F., Zimmermann R. (2017). An update on Sec61 channel function, mechanisms, and related diseases. Front. Physiol..

[47] Sicking M., Lang S., Bochen F., Drenth J.P.H., Zacharia M., Zimmermann R., Roos A., Linxweiler M. (2021). Complexity and specificity of Sec61 channelopathies: Human diseases affecting gating of the Sec61 complex. Cells.

[48] Walter P., Ibrahimi I., Blobel G. (1981). Translocation of proteins across the endoplasmic reticulum, I. Signal recognition protein (SRP) binds to in-vitro-assembled polysomes synthesizing secretory protein. J. Cell Biol..

[49] Walter P., Blobel G. (1981). Translocation of proteins across the endoplasmic reticulum, II. Signal recognition protein (SRP) mediates the selective binding to microsomal membranes of in-vitro-assembled polysomes synthesizing secretory protein. J. Cell Biol..

[50] Siegel V., Walter P. (1988). Functional dissection of the signal recognition particle. Trends Biochem. Sci..

[51] Hann B.C., Walter P. (1991). The signal recognition particle in Saccharomyces cerevisiae. Cell.

[52] Hann B.C., Stirling C.J., Walter P. (1992). SEC65 gene product is a subunit of the yeast signal recognition particle required for its integrity. Nature.

[53] Halic M., Beckmann R. (2005). The signal recognition particle and its interactions during protein targeting. Curr. Opin. Struct. Biol..

[54] Halic M., Blau M., Becker T., Mielke T., Pool M.R., Wild K., Sinning I., Beckmann R. (2006). Following the signal sequence from ribosomal tunnel exit to signal recognition particle. Nature.

[55] Voorhees R.M., Hegde R.S. (2015). Structures of the scanning and engaged states of the mammalian SRP-ribosome complex. eLife.

[56] Gamerdinger M., Hanebuth M.A., Frickey T., Deuerling E. (2015). The principle of antagonism ensures protein targeting specificity at the endoplasmic reticulum. Science.

[57] Hsieh H.-H., Lee J.H., Chandrasekar S., Shan S.O. (2020). A ribosome-associated chaperone enables substrate triage in a cotranslational protein targeting complex. Nat. Commun..

[58] Jomaa A., Eitzinger S., Zhu Z., Chandrasekar S., Kobajashi K., Shan S.-O., Ban N. (2021). Molecular mechanism of cargo recognition and handover by the mammalian signal recognition particle. Cell Rep..

[59] Jomaa A., Gamerdinger M., Hsieh H.-H., Wallisch A., Chandrasekaran V., Ulusoy Z., Scaiola A., Hegde R.S., Shan S.-O., Ban N. (2022). Mechanism of signal sequence handover from NAC to SRP on ribosomes during ER-protein targeting. Science.

[60] Meyer D.I., Dobberstein B. (1980). A membrane component essential for vectorial translocation of nascent proteins across the endoplasmic reticulum: Requirements for its extraction and reassociation with the membrane. J. Cell Biol..

[61] Gilmore R., Blobel G., Walter P. (1982). Protein translocation across the endoplasmic reticulum. I. Detection in the microsomal membrane of a receptor for the signal recognition particle. J. Cell Biol..

[62] Tajima S., Lauffer L., Rath V.L., Walter P. (1986). The signal recognition particle receptor is a complex that contains two distinct polypeptide chains. J. Cell Biol..

[63] Jan C.H., Williams C.C., Weissman J.S. (2014). Principles of ER cotranslational translocation revealed by proximity-specific ribosome profiling. Science.

[64] Chartron J.W., Hunt K.C.L., Frydman J. (2016). Cotranslational signal-independent SRP preloading during membrane targeting. Nature.

[65] Wang H., Hegde R.S. (2024). Identification of a factor that accelerates substrate release from the signal recognition particle. Science.

[66] Deshaies R.J., Koch B.D., Werner-Washburne M., Craig E.A., Schekman R. (1988). A subfamily of stress proteins facilitates translocation of secretory and mitochondrial precursor polypeptides. Nature.

[67] Rothblatt J.A., Deshaies R.J., Sanders S.L., Daum G., Schekman R. (1989). Multiple genes are required for proper insertion of secretory proteins into the endoplasmic reticulum in yeast. J. Cell Biol..

[68] Deshaies R.J., Sanders S.L., Feldheim D.A., Schekman R. (1991). Assembly of yeast Sec proteins involved in translocation into the endoplasmic reticulum into a membrane-bound multisubunit complex. Nature.

[69] Sanders S.L., Whitfield K.M., Vogel J.P., Rose M.D., Schekman R. (1992). Sec61p and BiP directly facilitate polypeptide translocation into the ER. Cell.

[70] Brodsky J.L., Goeckeler J., Schekman R. (1995). BiP and Sec63p are required for both co- and posttranslational protein translocation into the yeast endoplasmic reticulum. Proc. Natl. Acad. Sci. USA.

[71] Lyman S.K., Schekman R. (1995). Interaction between BiP and Sec63p is required for the completion of protein translocation into the ER of *Saccharomyces cerevisiae*. J. Cell Biol..

[72] Craven R.A., Egerton M., Stirling C.J. (1996). A novel Hsp70 of the yeast ER lumen is required for the efficient translocation of a number of protein precursors. EMBO J..

[73] Lyman S.K., Schekman R. (1997). Binding of secretory precursor polypeptides to a translocon subcomplex is regulated by BiP. Cell.

[74] Young B.P., Craven R.A., Reid P.J., Willer M., Stirling C.J. (2001). Sec63p and Kar2p are required for the translocation of SRP-dependent precursors into the yeast endoplasmic reticulum in vivo. EMBO J..

[75] Jung S.-j., Kim J.E.H., Reithinger J.H., Kim H. (2014). The Sec62–Sec63 translocon facilitates translocation of the C-terminus of membrane proteins. J. Cell Sci..

[76] Cohen N., Aviram N., Schuldiner M. (2023). A systematic proximity ligation approach to studying protein-substrate specificity identifies the substrate spectrum of the Ssh1 translocon. EMBO J..

[77] Schlenstedt G., Zimmermann R. (1987). Import of frog prepropeptide GLa into microsomes requires ATP but does not involve docking protein or ribosomes. EMBO J..

[78] Müller G., Zimmermann R. (1987). Import of honeybee prepromelittin into the endoplasmic reticulum: Structural basis for independence of SRP and docking protein. EMBO J..

[79] Müller G., Zimmermann R. (1988). Import of honeybee prepromelittin into the endoplasmic reticulum: Energy requirements for membrane insertion. EMBO J..

[80] Wiech H., Sagstetter M., Müller G., Zimmermann R. (1987). The ATP requiring step in the assembly of M 13 procoat protein into microsomes is related to preservation of transport competence of the precursor protein. EMBO J..

[81] Zimmermann R., Sagstetter M., Lewis M.J., Pelham H.R.B. (1988). Seventy-kilodalton heat shock proteins and an additional component from reticulocyte lysate stimulate import of M 13 procoat protein into microsomes. EMBO J..

[82] Schlenstedt G., Gudmundsson G.H., Boman H.G., Zimmermann R. (1990). A large presecretory protein translocates both cotranslationally, using signal recognition particle and ribosome, and posttranslationally, without these ribonucleoparticles, when synthesized in the presence of mammalian microsomes. J. Biol. Chem..

[83] Klappa P., Mayinger P., Pipkorn R., Zimmermann M., Zimmermann R. (1991). A microsomal protein is involved in ATP-dependent transport of presecretory proteins into mammalian microsomes. EMBO J..

[84] Klappa P., Freedman R., Zimmermann R. (1995). Protein disulfide isomerase and a lumenal cyclophilin-type peptidyl prolyl cis-trans isomerase are in transient contact with secretory proteins during late stages of translocation. Eur. J. Biochem..

[85] Dierks T., Volkmer J., Schlenstedt G., Jung C., Sandholzer U., Zachmann K., Schlotterhose P., Neifer K., Schmidt B., Zimmermann R. (1996). A microsomal ATP-binding protein involved in efficient protein transport into the mammalian endoplasmic reticulum. EMBO J..

[86] Tyedmers J., Lerner M., Bies C., Dudek J., Skowronek M.H., Haas I.G., Heim N., Nastainczyk W., Volkmer J., Zimmermann R. (2000). Homologs of the yeast Sec complex subunits Sec62p and Sec63p are abundant proteins in dog pancreas microsomes. Proc. Natl. Acad. Sci. USA.

[87] Mayer H.-A., Grau H., Kraft R., Prehn S., Kalies K.-U., Hartmann E. (2000). Mammalian Sec61 is associated with Sec62 and Sec63. J. Biol. Chem..

[88] Tyedmers J., Lerner M., Wiedmann M., Volkmer J., Zimmermann R. (2005). Polypeptide chain binding proteins mediate completion of cotranslational protein translocation into the mammalian endoplasmic reticulum. EMBO Rep..

[89] Müller L., Diaz de Escauriaza M., Lajoie P., Theis M., Jung M., Müller A., Burgard C., Greiner M., Snapp E.L., Dudek J. (2010). Evolutionary gain of function of the ER membrane protein Sec62 from yeast to humans. Mol. Biol. Cell.

[90] Shao S., Hegde R.S. (2011). A calmodulin-dependent translocation pathway for small secretory proteins. Cell.

[91] Schäuble N., Lang S., Jung M., Cappel S., Schorr S., Ulucan Ö., Linxweiler J., Dudek J., Blum R., Helms V. (2012). BiP-mediated closing of the Sec61 channel limits Ca^2+^ leakage from the ER. EMBO J..

[92] Lakkaraju A.K.K., Thankappan R., Mary C., Garrison J.L., Taunton J., Strub K. (2012). Efficient secretion of small proteins in mammalian cells relies on Sec62-dependent posttranslational translocation. Mol. Biol. Cell.

[93] Lang S., Benedix J., Fedeles S.V., Schorr S., Schirra C., Schäuble N., Jalal C., Greiner M., Haßdenteufel S., Tatzelt J. (2012). Different effects of Sec61α-, Sec62 and Sec63-depletion on transport of polypeptides into the endoplasmic reticulum of mammalian cells. J. Cell Sci..

[94] Johnson N., Vilardi F., Lang S., Leznicki P., Zimmermann R., High S. (2012). TRC-40 can deliver short secretory proteins to the Sec61 translocon. J. Cell Sci..

[95] Haßdenteufel S., Johnson N., Paton A.W., Paton J.C., High S., Zimmermann R. (2018). Chaperone-mediated Sec61 channel gating during ER import of small precursor proteins overcomes Sec61 inhibitor-reinforced energy barrier. Cell Rep..

[96] Haßdenteufel S., Nguyen D., Helms V., Lang S., Zimmermann R. (2019). Components and mechanisms for ER import of small human presecretory proteins. FEBS Lett..

[97] Ziska A., Tatzelt J., Dudek J., Paton A.W., Paton J.C., Zimmermann R., Haßdenteufel S. (2019). The signal peptide plus a cluster of positive charges in prion protein dictate chaperone-mediated Sec61-channel gating. Biol. Open.

[98] Schorr S., Nguyen D., Haßdenteufel S., Nagaraj N., Cavalié A., Greiner M., Weissgerber P., Loi M., Paton A.W., Paton J.C. (2020). Proteomics identifies signal peptide features determining the substrate specificity in human Sec62/Sec63-dependent ER protein import. FEBS J..

[99] Sun S., Li X., Mariappan M. (2023). Signal sequences encode information for protein folding in the endoplasmic reticulum. J. Cell Biol..

[100] Simon S.M., Blobel G. (1991). A protein-conducting channel in the endoplasmic reticulum. Cell.

[101] Görlich D., Hartmann E., Prehn S., Rapoport T.A. (1992). A protein of the endoplasmic reticulum involved early in polypeptide translocation. Nature.

[102] Görlich D., Prehn S., Hartmann E., Kalies K.-U., Rapoport T.A. (1992). A mammalian homolog of SEC61p and SECYp is associated with ribosomes and nascent polypeptides during translocation. Cell.

[103] Görlich D., Rapoport T.A. (1993). Protein translocation into proteoliposomes reconstituted from purified components of the endoplasmic reticulum membrane. Cell.

[104] Kalies K.-U., Rapoport T.A., Hartmann E. (1998). The beta-subunit of the Sec61 complex facilitates cotranslational protein transport and interacts with the signal peptidase during translocation. J. Cell Biol..

[105] Beckmann R., Spahn C.M., Eswar N., Helmers J., Penczek P.A., Sali A., Frank J., Blobel G. (2001). Architecture of the protein-conducting channel associated with the translating 80S ribosome. Cell.

[106] Wirth A., Jung M., Bies C., Frien M., Tyedmers J., Zimmermann R., Wagner R. (2003). The Sec61p complex is a dynamic precursor activated channel. Mol. Cell.

[107] Van den Berg B., Clemons W.M., Collinson I., Modis Y., Hartmann E., Harrison S.C., Rapoport T.A. (2004). X-ray structure of a protein-conducting channel. Nature.

[108] Devaraneni P.K., Conti B., Matsumara Y., Yang Z., Johnson A.E., Skach W.R. (2011). Stepwise insertion and inversion of a type II signal anchor sequence in the ribosome-Sec61 translocon complex. Cell.

[109] Pfeffer S., Brandt F., Hrabe T., Lang S., Eibauer M., Zimmermann R., Förster F. (2012). Structure and 3D arrangement of ER-membrane associated ribosomes. Structure.

[110] Pfeffer S., Dudek J., Gogala M., Schorr S., Linxweiler J., Lang S., Becker T., Beckmann R., Zimmermann R., Förster F. (2014). Structure of the mammalian oligosaccharyltransferase in the native ER protein translocon. Nat. Commun..

[111] Voorhees R.M., Fernández I.S., Scheres S.H.W., Hegde R.S. (2014). Structure of the mammalian ribosome-Sec61 complex to 3.4 Å resolution. Cell.

[112] Jadhav B., McKenna M., Johnson N., High S., Sinning I., Pool M.R. (2015). Mammalian SRP receptor switches the Sec61 translocase from Sec62 to SRP-dependent translocation. Nat. Commun..

[113] Conti B.J., Devaraneni P.K., Yang Z., David L.L., Skach W.R. (2015). Cotranslational stabilization of Sec62/63 within the ER Sec61 translocon is controlled by distinct substrate-driven translocation events. Mol. Cell.

[114] Voorhees R.M., Hegde R.S. (2016). Structure of the Sec61 channel opened by a signal peptide. Science.

[115] Pfeffer S., Burbaum L., Unverdorben P., Pech M., Chen Y., Zimmermann R., Beckmann R., Förster F. (2015). Structure of the native Sec61 protein-conducting channel. Nat. Commun..

[116] Mahamid J., Pfeffer S., Schaffer M., Villa E., Danev R., Kuhn Cuellar L., Förster F., Hyman A.A., Plitzko J.M., Baumeister W. (2016). Visualizing the molecular sociology at the HeLa cell nuclear periphery. Science.

[117] Gemmer M., Förster F. (2020). A clearer picture of the ER translocon complex. J. Cell Sci..

[118] Gemmer M., Chaillet M., van Loenhout J., Arenas R.C., Vismpas D., Gröllers-Mulderji M., Kohl F.A., Albanese P., Scheltema R.A., Howes S.C. (2023). Visualization of translation and protein biogenesis at the ER membrane. Nature.

[119] Mariappan M., Li X., Stefanovic S., Sharma A., Mateja A., Keenan R.J., Hegde R.S. (2010). A ribosome-associating factor chaperones tail-anchored membrane proteins. Nature.

[120] Leznicki P., Clancy A., Schwappach B., High S. (2010). Bat3 promotes the membrane integration of tail-anchored proteins. J. Cell Sci..

[121] Borgese N., Fasana E. (2011). Targeting pathways of C-tail-anchored proteins. Biochim. Biophys. Acta.

[122] Haßdenteufel S., Schäuble N., Cassella P., Leznicki P., Müller A., High S., Jung M., Zimmermann R. (2011). Calcium-calmodulin inhibits tail-anchored protein insertion into the mammalian endoplasmic reticulum membrane. FEBS Lett..

[123] Vilardi F., Lorenz H., Dobberstein B. (2011). WRB is the receptor for TRC40/Asna1-mediated insertion of tail-anchored proteins into the ER membrane. J. Cell Sci..

[124] Leznicki P., Warwicker J., High S. (2011). A biochemical analysis of the constraints of tail-anchored protein biogenesis. Biochem. J..

[125] Yamamoto Y., Sakisaka T. (2012). Molecular machinery for insertion of tail-anchored membrane proteins into the endoplasmic reticulum membrane in mammalian cells. Mol. Cell.

[126] Leznicki P., High S. (2012). SGTA antagonizes BAG6-mediated protein triage. Proc. Natl. Acad. Sci. USA.

[127] Hegde R.S., Keenan R.J. (2013). Tail-anchored membrane protein insertion into the endoplasmic reticulum. Nat. Rev. Mol. Cell Biol..

[128] Vilardi F., Stephan M., Clancy A., Janshoff A., Schwappach B. (2014). WRB and CAML are necessary and sufficient to mediate tail-anchored protein targeting to the ER membrane. PLoS ONE.

[129] Wang F., Chan C., Weir N.R., Denic V. (2014). The Get1/2 transmembrane complex is an endoplasmic-reticulum membrane protein insertase. Nature.

[130] Casson J., McKenna M., Haßdenteufel S., Aviram N., Zimmerman R., High S. (2017). Multiple pathways facilitate the biogenesis of mammalian tail-anchored proteins. J. Cell Sci..

[131] Zhang Y., De Laurentiis E., Bohnsack K.E., Wahlig M., Ranjan N., Gruseck S., Hackert P., Wölfle T., Rodnina M., Schwappach B. (2021). Ribosome-bound Get4/5 facilitate the capture of tail-anchored proteins by Sgt2 in yeast. Nat. Commun..

[132] Carvalho H.J.F., Del Bondio A., Maltecca F., Colombo S.F., Borgese N. (2019). The WRB subunit of the Get3 receptor is required for the correct integration of its partner CAML into the ER. Sci. Rep..

[133] McDowell M.A., Heimes M., Fiorentino F., Mehmood S., Farka A., Coy-Vergara J., Wu D., Bolla J.R., Schmid V., Heinze R. (2020). Structural basis of tail-anchored membrane protein biogenesis by the GET insertase complex. Mol. Cell.

[134] Leznicki P., High S. (2020). SGTA associates with nascent membrane protein precursors. EMBO Rep..

[135] Farkas A., Urlaub H., Bohnsack K.E., Schwappach B. (2022). Regulated targeting of the monotopic hairpin membrane protein Erg1 requires the GET pathway. J. Cell Biol..

[136] Wiedmann M., Kurzchalia T.V., Hartmann E., Rapoport T.A. (1987). A signal sequence receptor in the endoplasmic reticulum membrane. Nature.

[137] Fons R.D., Bogert B.A., Hegde R.S. (2003). Substrate-specific function of the translocon-associated protein complex during translocation across the ER membrane. J. Cell Biol..

[138] Sommer N., Junne T., Kalies K.-U., Spiess M., Hartmann E. (2013). TRAP assists membrane protein topogenesis at the mammalian ER membrane. Biochim. Biophys. Acta.

[139] Pfeffer S., Dudek J., Ng B., Schaffa M., Albert S., Plitzko J., Baumeister W., Zimmermann R., Freeze H., Engel B.D. (2017). Dissecting the molecular organization of the translocon-associatecd protein complex. Nat. Commun..

[140] Jaskolowski M., Jomaa A., Gamerdinger M., Shresta S., Leibundgut M., Deuerling E., Ban N. (2023). Molecular basis of the TRAP complex function in ER protein biogenesis. Nat. Struct. Mol. Biol..

[141] Pauwels E., Shewakramani N.R., De Wijngaert B., Camps A., Provinciael B., Stroobants J., Kalies K.-U., Hartmann E., Maes P., Vermeire K. (2023). Structural insights into TRAP association with ribosome-Sec61 complex and translocon inhibition by a CADA derivative. Sci. Adv..

[142] Wilson R., Allen A.J., Oliver J., Brookman J.L., High S., Bulleid N. (1995). The translocation, folding, assembly, and redox-dependent degradation of secretory and membrane proteins in semi-permeabilized mammalian cells. Biochem. J..

[143] Haßdenteufel S., Klein M.-C., Melnyk A., Zimmermann R. (2014). Protein transport into the human ER and related diseases: Sec61-channelopathies. Biochem. Cell Biol..

[144] Schubert D., Klein M.-C., Haßdenteufel S., Caballero-Oteyza A., Yang L., Proietti M., Bulashevska A., Kemming J., Kühn J., Winzer S. (2018). Plasma cell deficiency in human subjects with heterozygous mutations in Sec61 translocon alpha 1 (*SEC61A1*). J. Allergy Clin. Immunol..

[145] Van Nieuwenhove E., Barber J., Smeets E., Neumann J., Willemsen M., Pasciuto E., Prezzemolo T., Lagou V., Seldeslachts L., Malengier-Devlies B. (2020). Defective Sec61α1 underlies a novel cause of autosomal dominant severe congenital neutropenia. J. Allergy Clin. Immunol..

[146] Bolar N.A., Golzio C., Živná M., Hayot G., Van Hemelrijk C., Schepers D., Vandeweyer G., Hoischen A., Huyghe J.R., Raes A. (2016). Heterozygous Loss-of-Function SEC61A1 Mutations Cause Autosomal-Dominant Tubulo-Interstitial and Glomerulocystic Kidney Disease with Anemia. Am. J. Hum. Genet..

[147] Fedeles S.V., Tian X., Gallagher A.-R., Mitobe M., Nishio S., Lee S.H., Cai Y., Geng L., Crews C.M., Somlo S. (2011). A genetic interaction network of five genes for human polycystic kidney and liver disease defines polycystin-1 as the central determinant of cyst formation. Nat. Genet..

[148] Besse W., Dong K., Choi J., Punia S., Fedeles S.V., Choi M., Gallagher A.-R., Huang E.B., Gulati A., Knight J. (2017). Isolated polycystic liver disease genes define effector s of polycystin-1 function. J. Clin. Investig..

[149] Wilson M.P., Durin Z., Unal Ö., Ng B.G., Marrecau T., Keldermans L., Souche E., Rymen D., Gündüz M., Köse G. (2022). CAMLG-CDG: A novel Congenital Disorder of Glycosylation linked to defective membrane trafficking. Hum. Mol. Genet..

[150] Weiand M., Sandfort V., Nadzemova O., Schierwagen R., Trebicka J., Schlevogt B., Kabar I., Schmidt H., Zibert A. (2024). Comparative analysis of SEC61A1 mutant R236C in two patient-derived cellular platforms. Sci. Rep..

[151] Ng B.G., Freeze H.F., Himmelreich N., Blau N., Ferreira C.R. (2024). Clinical and biochemical footprints of congenital disorders of glycosylation: Proposed nosology. Mol. Genet. Metab..

[152] Sicking M., Živná M., Bhadra P., Barešová V., Tirincsi A., Hadzibeganovic D., Hodaňová K., Vyleťal P., Sovová J., Jedličkova I. (2022). Phenylbutyrate rescues the transport defect of the Sec61α mutations V67G and T185A for renin. Life Sci. Alliance.

[153] Erdmann F., Schäuble N., Lang S., Jung M., Honigmann A., Ahmad M., Dudek J., Benedix J., Harsman A., Kopp A. (2011). Interaction of calmodulin with Sec61a limits Ca^2+^ leakage from the endoplasmic reticulum. EMBO J..

[154] Aviram N., Ast T., Costa E.A., Arakel E., Chuartzman S.G., Jan C.H., Haßdenteufel S., Dudek J., Jung M., Schorr S. (2016). The SND proteins constitute an alternative targeting route to the endoplasmic reticulum. Nature.

[155] Haßdenteufel S., Sicking M., Schorr S., Aviram N., Fecher-Trost C., Schuldiner M., Jung M., Zimmermann R., Lang S. (2017). hSnd2 protein represents an alternative targeting factor to the endoplasmic reticulum in human cells. FEBS Lett..

[156] Tirincsi A., O’Keefe S., Nguyen D., Sicking M., Dudek J., Förster F., Jung M., Hadzibeganovic D., Helms V., High S. (2022). Proteomics identifies substrates and a novel component in hSnd2-dependent ER protein targeting. Cells.

[157] Zimmermann R., Lang S., Lerner M., Förster F., Nguyen D., Helms V., Schrul B. (2021). Quantitative proteomics and differential protein abundance ananalysis after depletion of PEX3 from human cells identifies additional aspects of protein targeting to the ER. Int. J. Mol. Sci..

[158] Lang S., Erdmann F., Jung M., Wagner R., Cavalié A., Zimmermann R. (2011). Sec61 complexes form ubiquitous ER Ca^2+^ leak channels. Channels.

[159] Schorr S., Klein M.-C., Gamayun I., Melnyk A., Jung M., Schäuble N., Wang Q., Hemmis B., Bochen F., Greiner M. (2015). Co-chaperone specificity in gating of the polypeptide conducting channel in the membrane of the human endoplasmic reticulum. J. Biol. Chem..

[160] Linxweiler M., Schorr S., Jung M., Schäuble N., Linxweiler J., Langer F., Schäfers H.-J., Cavalié A., Zimmermann R., Greiner M. (2013). Targeting cell migration and the ER stress response with calmodulin antagonists: A clinically tested small molecule phenocopy of SEC62 gene silencing in human tumor cells. BMC Cancer.

[161] Gumbart J., Schulten K. (2007). Structural determinants of lateral gate opening in the protein translocon. Biochemistry.

[162] Zhang B., Miller T.F. (2012). Long-timescale dynamics and regulation of Sec-facilitated protein translocation. Cell Rep..

[163] Savitz A.J., Meyer D.I. (1990). Identification of a ribosome receptor in the rough endoplasmic reticulum. Nature.

[164] Seiser R.M., Nicchitta C.V. (2000). The fate of membrane-bound ribosomes following the termination of protein synthesis. J. Biol. Chem..

[165] Potter M.D., Seiser R.M., Nicchitta C.V. (2001). Ribosome exchange revisited: A mechanism for translation-coupled ribosome detachment from the ER membrane. Trends Cell Biol..

[166] Berkovits B.D., Mayr C. (2015). Alternative 3′UTRs act as scaffolds to regulate membrane protein localization. Nature.

[167] Ma W., Mayr C. (2018). A membraneless organelle associated with the endoplasmic reticulum enables 3′UTR-mediated protein-protein interactions. Cell.

[168] Hsu J.C.-C., Reid D.W., Hoffman A.M., Sarkar D., Nicchitta C.V. (2018). Oncoprotein AEG-1 is an endoplasmic reticulum RNA-binding protein whose interactome is enriched in organelle resident protein-encoding mRNAs. RNA.

[169] Hannigan M.M., Hoffman A.M., Thompson J.W., Zheng T., Nicchitta C.V. (2020). Quantitative proteomics links the LRRC59 interactome to mRNA translation on the ER membrane. Mol. Cell. Proteom..

[170] Bhadra P., Schorr S., Lerner M., Nguyen D., Dudek J., Förster F., Helms V., Lang S., Zimmermann R. (2021). Quantitative proteomics and differential protein abundance analysis after depletion of putative mRNA receptors in the ER membrane of human cells identifies novel aspects of mRNA targeting to the ER. Molecules.

[171] Horste E.L., Fansler M.M., Cai T., Chen X., Mitschka S., Zhen G., Lee F.C.Y., Ule J., Mayr C. (2023). Subcytoplasmic location of translation controls protein output. Mol. Cell.

[172] Child J.R., Hofler A.C., Chen Q., Yang B.H., Kristofich J., Zheng T., Hannigan M.M., Elles A.L., Reid D.W., Nicchitta C.V. (2023). Examining SRP pathway function in mRNA localizytion to the endoplasmic reticulum. RNA.

[173] Hirata T., Yang J., Tomida S., Tokoro Y., Kinoshita T., Fujita M., Kizuka Y. (2022). ER entry pathway and glycosylation of GPI-anchored proteins are determined by N-terminal signal sequence and C-terminal GPI-attachment sequence. J. Biol. Chem..

[174] Talbot B.E., Vandorpe D.H., Stotter B.R., Alper S.L., Schlondorff J. (2019). Transmembrane insertases and N-glycosylation crtically determine synthesis, trafficking, and activity of the nonselective cation channel TRPC6. J. Biol. Chem..

[175] Yang J., Hirata T., Liu Y.S., Guo X.Y., Gao X.-D., Kinoshita T., Fujita M. (2021). Human SND2 mediates ER targeting of GPI-anchored proteins with low hydrophobic GPI attachment signals. FEBS Lett..

[176] Anghel S.A., McGilvray P.T., Hegde R.S., Keenan R.J. (2017). Identification of Oxa1 homologs operating in the eukaryotic endoplasmic reticulum. Cell Rep..

[177] Chitwood P.J., Juszkiewicz S., Guna A., Shao S., Hegde R.S. (2018). EMC is required to initiate accurate membrane protein topogenesis. Cell.

[178] Shurtleff M.J., Itzhak D.N., Hussmann J.A., Schirle Oakdale N.T., Costa E.A., Jonikas M., Weibezahn J., Popova K.D., Jan C.H., Sinitcyn P. (2018). The ER membrane protein complex interacts cotranslationally to enable biogenesis of multipass membrane proteins. eLife.

[179] Tian S., Wu Q., Zhou B., Choi M.Y., Ding B., Yang W., Dong M. (2019). Proteomic analysis indentifies membrane proteins dependent on the ER membrane protein complex. Cell Rep..

[180] McGilvray P.T., Anghel S.A., Sundaram A., Zhong F., Trnka M.J., Fuller J.R., Hu H., Burlingame A.L., Keenan R.J. (2020). An ER translocon for multi-pass mambrane protein biogenesis. eLife.

[181] O’Donnel J.P., Philips B.P., Yagita Y., Juszkiewicz S., Wagner A., Malinverni D., Keenan R.J., Mille E.A., Hegde R.S. (2020). The architecture of EMC reveals a path for membrane protein insertion. eLife.

[182] Pleiner T., Tomaleri G.P., Januszyk K., Inglis A.J., Hazu M., Voorhees R.M. (2020). Structural basis for membrane insertion by the human ER membrane protein complex. Science.

[183] Bai L., You Q., Feng X., Kovach A., Li H. (2020). Structure of the ER membrane complex, a transmembrane insertase. Nature.

[184] O’Keefe S., Zong G., Duah K.B., Andrews L.E., Shi W.Q., High S. (2021). An alternative pathway for membrane protein biogenesis at the endoplasmic reticulum. Commun. Biol..

[185] Kizmaz B., Flohr T., Garg S.G., Herrmann J.M. (2022). The ER membrane complex (EMC) can functionally replace the Oxa1 insertase in mitochondria. PLoS Biol..

[186] Meacock S.L., Lecomte F.J.L., Crawshaw S.G., High S. (2002). Different transmembrane domains associate with distinct endoplasmic reticulum components during membrane integration of a polytopic protein. Mol. Biol. Cell.

[187] Sundaram A., Yamsek M., Zhong F., Hooda Y., Hegde R.S., Keenan R.J. (2022). Substrate-driven assembly of a translocon for multipass membrane proteins. Nature.

[188] Samlinskaite L., Kim M.K., Lewis A.J.O., Keenan R.J., Hegde R.S. (2022). Mechanism of an intramembrane chaperone for multipass membrane proteins. Nature.

[189] Wu H., Hegde R.S. (2023). Mechanism of signal-anchor triage during early steps of membrane protein insertion. Mol. Cell.

[190] Page K.R., Nguyen V.N., Pleiner T., Tomaleri G.P., Wang M.L., Guna A., Hazu M., Wang T.-Y., Chou T.-F., Voorhees R.M. (2024). Role of the holo-insertase complex in the biogensis of biophysically diverse ER membrane proteins. Mol. Cell.

[191] Wiedmann B., Saki H., Davis T.A., Wiedmann M. (1994). A protein complex required for signal-sequence-specific sorting and translocation. Nature.

[192] Moeller I., Jung M., Beatrix B., Levy R., Kreibich G., Zimmermann R., Wiedmann M., Lauring B. (1998). A general mechanism for regulation of access to the translocon: Competition for a membrane attachment site on ribosomes. Proc. Natl. Acad. Sci. USA.

[193] Gamerdinger M., Kobayashi K., Wallisch A., Kreft S.G., Sailer C., Schlömer R., Sachs N., Jomaa A., Stengel F., Ban N. (2019). Early scanning of nascent polypeptides inside the ribosomal tunnel by NAC. Mol. Cell.

[194] Gamerdinger M., Deuerling E. (2024). Cotranslational sorting and processing of newly synthesized proteins in eukaryotes. Trends Biochem. Sci..

[195] Forte G.M., Pool M.R., Stirling C.J. (2011). N-terminal acetylation inhibits protein targeting to the endoplasmic reticulum. PLoS Biol..

[196] Aksnes H., Van Damme P., Goris M., Starheim K.K., Marie M., Stove S.I., Hoel C., Kalvik T.V., Hole K., Glomes N. (2015). An organellar N-acetyltransferase Naa60, acetylates cytosolic N termini of transmembrane proteins and maintains Golgi integrity. Cell Rep..

[197] Aksnes H., Drazic A., Marie M., Arnesen T. (2016). First things first: Vital protein marks by N-terminal acetyltransferases. Trends Biochem. Sci..

[198] Knorr A.G., Schmidt C., Tesina P., Berninghausen O., Becker T., Beatrix B., Beckmann R. (2019). Ribosome-NatA architecture reveals that rRNA expansion segments coordinate N-terminal acetylation. Nat. Struct. Mol. Biol..

[199] Tanco S., Jonckheere V., Tharkeshwar A.K., Bogaert A., Gevaert K., Van Damme P. (2025). Proximal partners of organellar N-terminal acetyltransferase NAA60: Insights into Golgi structure and transmembrane topology. Open Biol..

[200] Nguyen D., Stutz R., Schorr S., Lang S., Pfeffer S., Freeze H.F., Förster F., Helms V., Dudek J., Zimmermann R. (2018). Proteomics reveals signal peptide features determining the client specificity in human TRAP-dependent ER protein import. Nat. Commun..

[201] Klein M.-C., Lerner M., Nguyen D., Pfeffer S., Dudek J., Förster F., Helms V., Lang S., Zimmermann R. (2020). TRAM1 protein may support ER protein import by modulating the phospholipid bilayer near the lateral gate of the Sec61 channel. Channels.

[202] High S., Martoglio B., Görlich D., Andersen S.S.L., Ashford A.A., Giner A., Hartmann E., Prehn S., Rapoport T.A., Dobberstein B. (1993). Site-specific photocross-linking reveals that Sec61p and TRAM contact different regions of a membrane-inserted signal sequence. J. Biol. Chem..

[203] Hegde R.S., Voigt S., Rapoport T.A., Lingappa V.R. (1998). TRAM regulates the exposure of nascent secretory proteins to the cytosol during translocation into the endoplasmic reticulum. Cell.

[204] Voigt S., Jungnickel B., Hartmann E., Rapoport T.A. (1996). Signal sequence-dependent function of the TRAM protein during early phases of protein transport across the endoplasmic reticulum membrane. J. Cell Biol..

[205] Sauri A., McCormick P.J., Johnson A.E., Mingarro I. (2007). Sec61alpha and TRAM are sequentially adjacent to a nascent viral membrane protein during its ER integration. J. Mol. Biol..

[206] Itskanov S., Park E. (2019). Structure of the posttranslational Sec protein-translocation channel complex from yeast. Science.

[207] Wu X., Cabanos C., Rapoport T.A. (2019). Structure of the post-translational protein translocation machinery of the ER membrane. Nature.

[208] Itskanov S., Kuo K.M., Gumbart J.C., Park E. (2021). Stepwise gating of the Sec61 protein-conducting channel by Sec62 and Sec63. Nat. Struct. Mol. Biol..

[209] Weng T.-H., Steinchen W., Beatrix B., Berninghausen O., Becker T., Bange G., Cheng J., Beckmann R. (2021). Architecture of the active post-translational SEC translocon. EMBO J..

[210] Dudek J., Volkmer J., Bies C., Guth S., Müller A., Lerner M., Feick P., Schäfer K.H., Morgenstern E., Hennessy F. (2002). A novel type of cochaperone mediates transmembrane recruitment of DnaK-like chaperones to ribosomes. EMBO J..

[211] Dudek J., Greiner M., Müller A., Hendershot L.M., Kopsch K., Nastainczyk W., Zimmermann R. (2005). ERj1p plays a basic role in protein biogenesis at the endoplasmic reticulum. Nat. Struct. Mol. Biol..

[212] Blau M., Mullapudi S., Becker T., Dudek J., Zimmermann R., Penczek P.A., Beckmann R. (2005). ERj1p uses a universal ribosomal adaptor site to coordinate the 80S ribosome at the membrane. Nat. Struct. Mol. Biol..

[213] Benedix J., Lajoie P., Jaiswal H., Burgard C., Greiner M., Zimmermann R., Rospert S., Snapp E.L., Dudek J. (2010). BiP modulates the affinity of its co-chaperone ERj1 to ribosomes. J. Biol. Chem..

[214] Song J., Mizrak A., Lee C.W., Cicconet M., Lai Z.W., Tang W.-C., Mohr S.E., Farese R.V., Walther T.C. (2022). Identification of two pathways mediating protein targeting from ER to lipid droplets. Nat. Cell Biol..

[215] Voeltz G.K., Prinz W.A., Shibata Y., Rist J.M., Rapoport T.A. (2006). A class of membrane proteins shaping the tubular endoplasmic reticulum. Cell.

[216] Allen K.N., Entova S., Ray L.C., Imperiali B. (2019). Monotopic membrane proteins join the fold. Trends Biochem. Sci..

[217] Yang T.-J., Mukherjee S., Langer J.D., Hummer G., McDowell M.A. (2025). SND3 is the membrane insertase within a fungal multipass translocon. bioRxiv.

[218] Javaneinen M., Simek J., Tranter D., O’Keefe S., Karki S., Biriukov D., Sachl R., Paavvilainen V.O. (2025). Lipid scrambling pathways in the Sec61 translocon complex. J. Am. Chem. Soc..

[219] Pool M. (2022). Targeting of proteins for translocation at the endoplasmic reticulum. Int. J. Mol. Sci..

[220] Hsieh H.-H., Shan S.-O. (2022). Fidelity of cotranslational protein targeting to the endoplasmic reticulum. Int. J. Mol. Sci..

[221] Bhadra P., Helms V. (2021). Molecular modeling of signal peptide recognition by eukaryotic Sec complexes. Int. J. Mol. Sci..

[222] Liaci A.M., Förster F. (2021). Take me home, protein roads: Structural insigths into signal peptide interactions during ER translocation. Int. J. Mol. Sci..

[223] Jung S.-j., Kim H. (2021). Emerging view on the molecular functions of Sec62 and Sec63 in protein translocation. Int. J. Mol. Sci..

[224] Tirincsi A., Sicking M., Hadzibeganovic D., Haßdenteufel S., Lang S. (2021). The molecular biodiversity of protein targeting and protein transport related to the endoplasmic reticulum. Int. J. Mol. Sci..

[225] Whitley P., Grau B., Gumbart J.C., Martinez-Gil L., Mingarro I. (2021). Folding and insertion of transmembrane helices at the ER. Int. J. Mol. Sci..

[226] Sanchez W.N., Driessen A.J.M., Wilson C.A.M. (2025). Protein targeting to the ER membrane: Multiple pathways and shared machinery. Crit. Rev. Biochem. Mol. Biol..

[227] Gruss O.J., Feick P., Frank R., Dobberstein B. (1999). Phsophorylation of components of the ER translocation site. Eur. J. Biochem..

[228] Faust M., Jung M., Günther J., Zimmermann R., Montenarh M. (2001). Localization of individual subunits of protein kinase CK2 to the endoplasmic reticulum and to the Golgi apparatus. Mol. Cell. Biochem..

[229] Götz C., Müller A., Montenarh M., Zimmermann R., Dudek J. (2009). ERj1 is a substrate of phosphorylation by CK2. Biochem. Biophys. Res. Commun..

[230] Ampofo E., Welker S., Jung M., Müller L., Greiner M., Zimmermann R., Montenarh M. (2013). CK2 phosphorylation of human Sec63 regulates its interaction with Sec62. Biochim. Biophys. Acta.

[231] Zhang Z., Xu A., Bai Y., Chen Y., Cates K., Kerr C., Bermudez A., Susanto T.T., Wysong K., Marquez F.J.G. (2015). A subcellular map of translational machinery composition and regulation at the single-molecule level. Science.

[232] Garrison J.L., Kunkel E.J., Hegde R.S., Taunton J. (2005). A substrate-specific inhibitor of protein translocation into the endoplasmic reticulum. Nature.

[233] Besemer J., Harent H., Wang S., Oberhauser B., Marquardt K., Foster C.A., Schreiner E.P., de Vries J.E., Dascher-Nadel C., Lindley I.J.D. (2005). Selective inhibition of cotranslational translocation of vascular cell adhesion molecule 1. Nature.

[234] Cross B.C.S., McKibbin C., Callan A.C., Roboti P., Piacenti M., Rabu C., Wilson C.M., Whitehead R., Flitsch S.L., Pool M.R. (2009). Eeyarestatin I inhibits Sec61-mediated protein translocation at the endoplasmic reticulum. J. Cell Sci..

[235] Hall B.S., Hill K., McKenna M., Ogbechi J., High S., Willis A.E., Simmonds R.E. (2014). The pathogenic mechanism of the Mycobacterium ulcerans virulence factor, Mycolactone, depends on blockade of protein translocation into the ER. PloS Pathog..

[236] MacKinnon A.L., Paavilainen V.O., Sharma A., Hegde R.S., Taunton J. (2014). An allosteric Sec61 inhibitor traps nascent transmembrane helices at the lateral gate. eLife.

[237] Paatero A.O., Kellosalo J., Dunyak B.M., Almaliti J., Gestwicki J.E., Gerwick W.H., Taunton J., Paavilainen V.O. (2016). Apratoxin kills cells by direct blockade of the Sec61 protein translocation channel. Cell Chem. Biol..

[238] Baron L., Paatero A.O., Morel J.-D., Impens F., Guenin-Macé L., Saint-Auret S., Blanchard N., Dillmann R., Niang F., Pellegrini S. (2016). Maycolactone subervts immunity by selectively blocking the Sec61 translocon. J. Exp. Med..

[239] Pauwels E., Schülein R., Vermeire K. (2021). Inhibitors of the Sec61 complex and novel high throughput screening strategies to target the protein translocation pathway. Int. J. Mol. Sci..

[240] Pauwels E., Provinciael B., Camps A., Hartmann E., Vermeire K. (2022). Reduced DNAJC3 expression affects protein translocation across the ER membrane and attenuates the down-modulating effect of the translocation inhibitor cyclotriazadisulfonamide. Int. J. Mol. Sci..

[241] Zimmermann J.S.M., Linxweiler J., Radosa J., Linxweiler M., Zimmermann R. (2022). The ER membrane protein Sec62 as potential therapeutic target in SEC62 overexpressing tumors. Front. Physiol..

[242] Aksnes H., Ree R., Arnesen T. (2019). Co-translational, post-translational, and non-catalytic roles of N-acetyltransferases. Mol. Cell.

[243] Lentzsch A.M., Yudin D., Gamerdinger M., Chandrasekar S., Rabl L., Scaiola A., Deuerling E., Ban N., Shan S.-O. (2024). NAC guides a ribosomal multienzyme complex for nascent protein processing. Nature.

[244] Pfeiffer N.V., Dirndorfer D., Lang S., Resenberger U.K., Restelli L.M., Hemion C., Miesbauer M., Frank S., Neutzner A., Zimmermann R. (2013). Structural features within the nascent chain regulate alternative targeting of secretory proteins to mitochondria. EMBO J..

[245] Mick D.U., Rodriguez R.B., Leib R.D., Adams C.M., Chien A.S., Gygi S.P., Nachuri M.V. (2015). Proteomics of primary cilia by proximity labeling. Dev. Cell.

[246] May E.A., Kalocsay M., D’Auriac I.G., Schuster P.S., Gygi S.P., Nachury M.V., Mick D.U. (2021). Time-resolved proteomics profiling of the ciliary hedgehog response. J. Cell Biol..

[247] Go C.D., Knight J.D.R., Rajasekharan A., Rathod B., Hesketh G.G., Abe K.T., Youn J.-Y., Samavarchi-Tehrani P., Zhang H., Zhu L.Y. (2021). A proximity-dependent biotinylation map of a human cell. Nature.

[248] Sicking M., Jung M., Lang S. (2021). Lights, camera, interaction: Studying protein-protein interactions of the ER protein translocase in living cells. Int. J. Mol. Sci..

