# Supplementary material for: Rules of Engagement for Components of Membrane Protein Biogenesis at the Human Endoplasmic Reticulum
Source: Int J Mol Sci. 2025 Sep 10;26(18):8823. doi: 10.3390/ijms26188823 (PMC12469465; doi:10.3390/ijms26188823)
Supplement: Supplementary file 1 [file ijms-26-08823-s001.zip › supplementary files/IJMS-3803115_Figure S3.pdf]

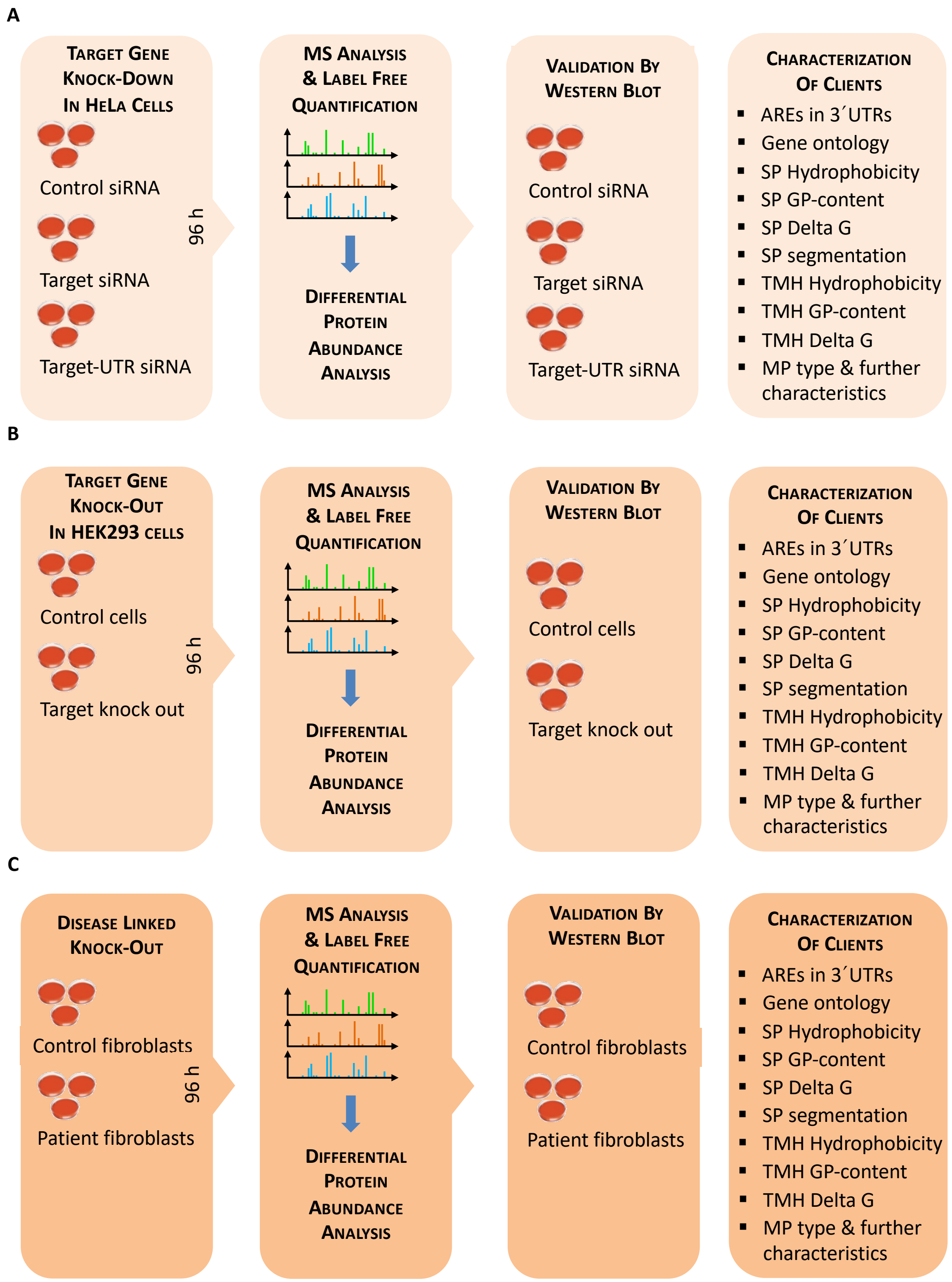

Figure S3. The experimental approach for the characterization of the clients of ER protein import components and their distinguishing features. The Figure and its legend were adapted from Lang et al. [18]. (**A**) Briefly, the experimental strategy involved i) siRNA-mediated gene silencing in HeLa cells for 96 h; ii) label-free quantitative analysis of the total cellular proteome; iii) differential protein abundance analysis to identify negatively affected proteins (i.e. putative clients of the target and putative physical interaction partners) as well as positively affected proteins (potential genetic interactors); iv) independent validation experiments. (**B**, **C**) Alternatively, target knock-out cells were obtained either by CRISPR/Cas9 treatment of HEK293 cells or in form of patient fibroblasts.
