# Supplementary material for: Rules of Engagement for Components of Membrane Protein Biogenesis at the Human Endoplasmic Reticulum
Source: Int J Mol Sci. 2025 Sep 10;26(18):8823. doi: 10.3390/ijms26188823 (PMC12469465; doi:10.3390/ijms26188823)
Supplement: Supplementary file 1 [file ijms-26-08823-s001.zip › supplementary files/IJMS-3803115_Figure S2.pdf]

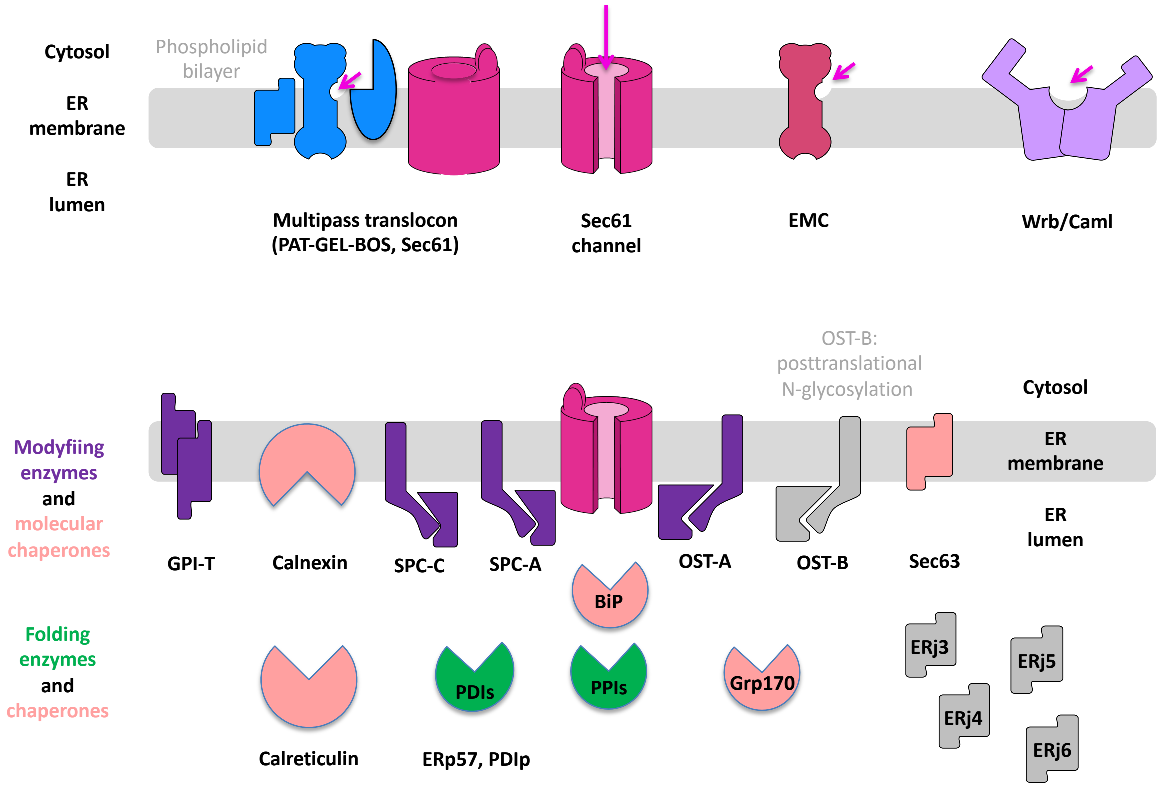

Figure S2. Molecular chaperones and enzymes for covalent modifications of polypeptide chains in the ER. The long arrow (in magenta) points to the open aqueous channel and open lateral gate, respectively, of the fully open Sec61 channel, the short arrows (in magenta) point to the characteristic hydrophilic vestibules of the MP insertases that are related to Oxa1 (specifically the subunits EMC3, TMCO1 and Wrb). The Figure was adapted from Tirincsi et al [174]. See Figure 3, Table S1 and text for further details.
