# Supplementary material for: Rules of Engagement for Components of Membrane Protein Biogenesis at the Human Endoplasmic Reticulum
Source: Int J Mol Sci. 2025 Sep 10;26(18):8823. doi: 10.3390/ijms26188823 (PMC12469465; doi:10.3390/ijms26188823)
Supplement: Supplementary file 1 [file ijms-26-08823-s001.zip › supplementary files/IJMS-3803115_Figure S1.pdf]

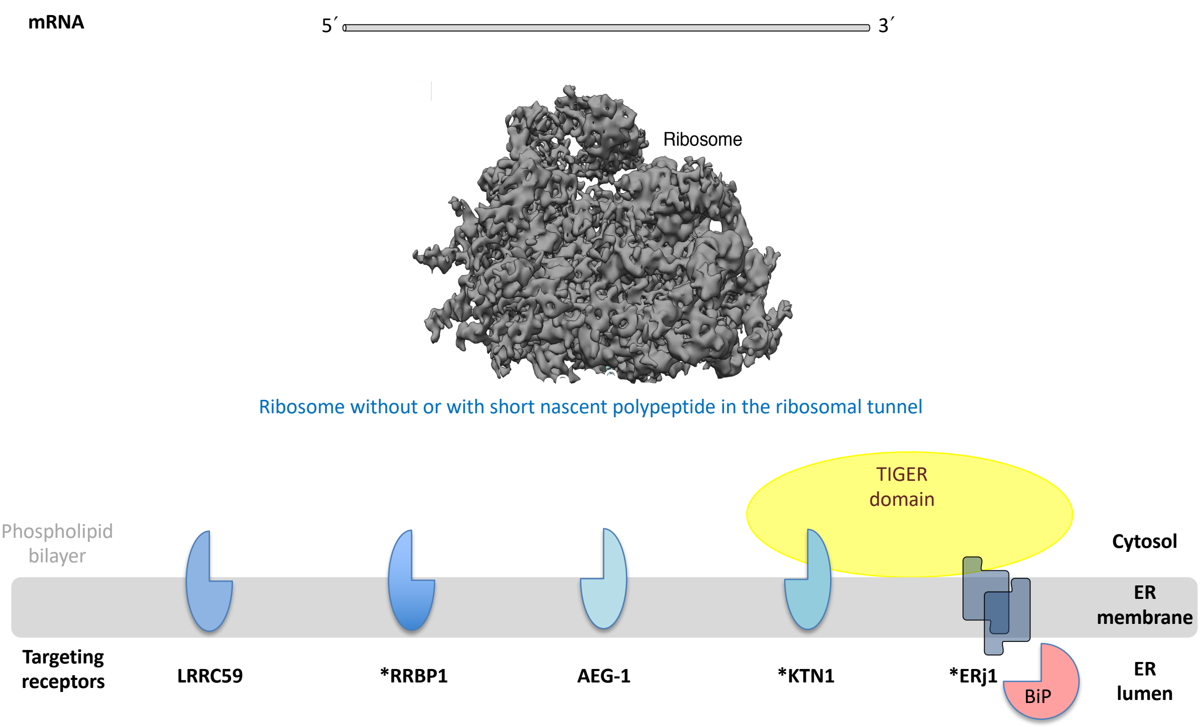

Figure S1. Components for targeting of mRNAs, ribosomes and ribosome-nascent chain complexes to receptors in the ER membrane. ER protein import comprises a targeting step plus a translocation or membrane insertion step of precursor polypeptides and may, additionally, involve targeting of mRNAs or ribosomes with or without short nascent polypeptide chains to the respective receptors at the ER membrane, such as LRRC59, RRBP1, AEG-1, KTN1 [166]. However, the coiled-coil proteins KTN1 and RRBP1 have additional functions, such as orchestrating shape and cellular distribution of the ER and, therefore, can be expected to also have indirect effects when depleted. The TIGER domain represents a cytosolic micro-domain, enriched in MP-encoding mRNAs with multiple AU-rich elements or AREs in their 3´UTRs in the vicinity of the ER [163,167]. In case of ER-targeting of mRNAs that code for cytosolic proteins, NAC can bind to the amino-terminus of the nascent polypeptides and trigger their release from the Sec61 complex [191,192]. The Figure was adapted from Jung & Zimmermann [43]. Notably, LRRC59 was detected as genetic interaction partner of Sec62, i.e. upregulated under conditions of Sec62 depletion (Figure 7) [94], and KTN1 was detected as physical interaction partner of TRAPγ by chemical crosslinking of human rough microsomes and subsequent mass spectrometry of tryptic peptides (M. Jung, Homburg, unpublished results). The asterisks (*) highlight the components that were interrogated by depletion in human cells in combination with quantiative MS and differential protein abundance analysis [166]. The ribosomal image does not differentiate between presence or absence of a short nascent polypeptide chain with < 50 amino acid residues because the chain would be hidden in the ribosomal tunnel.
