# Supplementary material for: Rules of Engagement for Components of Membrane Protein Biogenesis at the Human Endoplasmic Reticulum
Source: Int J Mol Sci. 2025 Sep 10;26(18):8823. doi: 10.3390/ijms26188823 (PMC12469465; doi:10.3390/ijms26188823)
Supplement: Supplementary file 1 [file ijms-26-08823-s001.zip › supplementary files/IJMS-3803115_Figure S5.pdf]

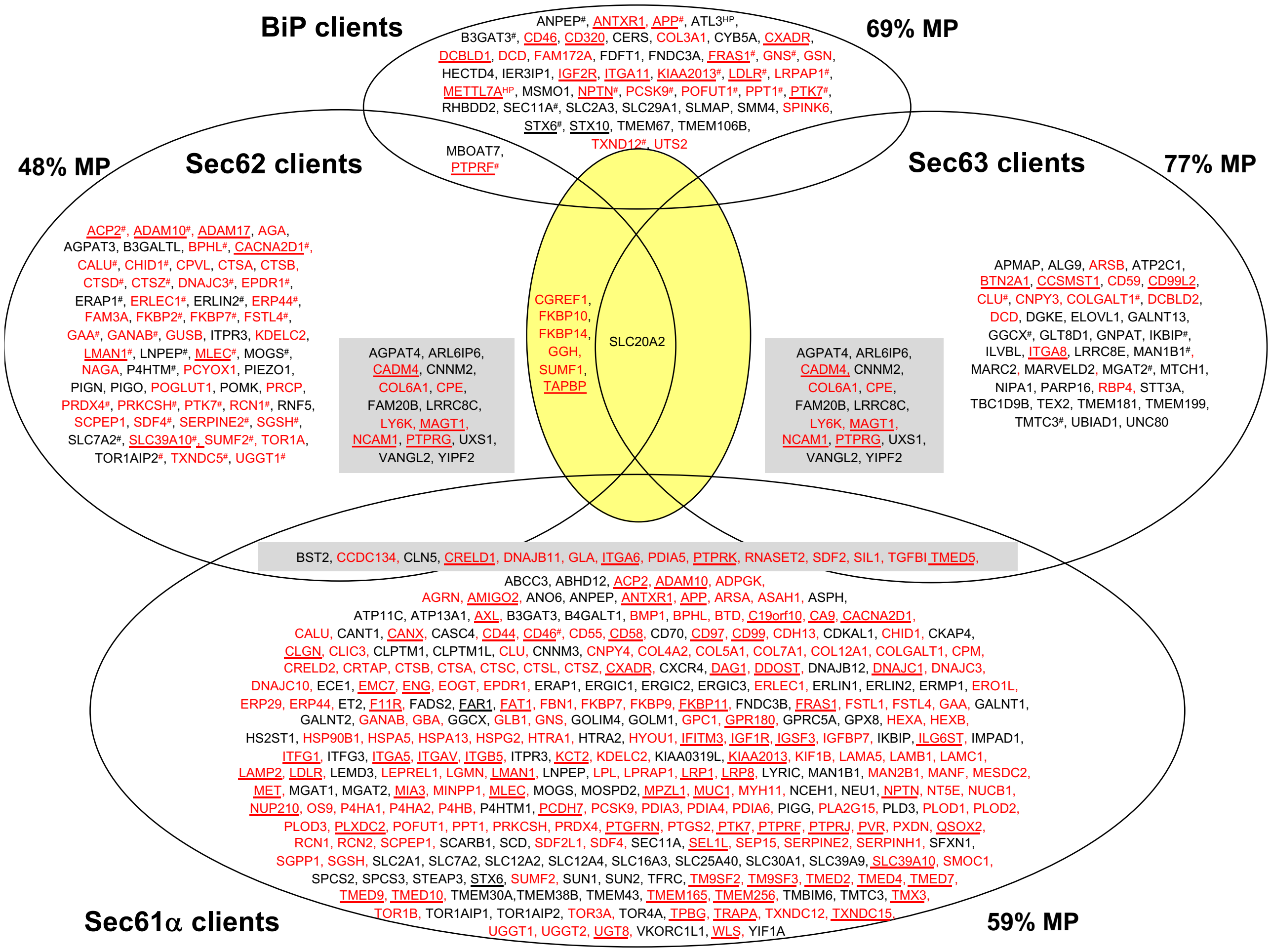

Figure S5. Venn diagram for the putative clients of BiP and various components for protein biogenesis at the human ER. The clients were determined by quantitative MS and differential protein abundance analysis following depletion of the respective component in HeLa cell for 72 or 96 h. Clients are defined as such by the presence of either an SP or at least one TMH. Shown with their gene names, clients with SPs are shown in red, SP containing membrane proteins are underlined, clients with TMH are shown in black, TA membrane proteins are underlined, hairpin proteins are indicated by superscript HP and italics highlight clients of two targeting components, which could not be properly fitted into the Venn diagram and are named twice. The overlaps in clients between Sec61α and Sec62 plus Sec63 as well as between Sec62 and Sec63 are highlighted in grey, between BiP and Sec61α plus Sec62 as well as between BiP, Sec61α, Sec62 plus Sec63 in yellow. The data were previously reported by Nguyen et al., 2018 [200] and Schorr et al., 2020 [94].
