# Supplementary material for: Rules of Engagement for Components of Membrane Protein Biogenesis at the Human Endoplasmic Reticulum
Source: Int J Mol Sci. 2025 Sep 10;26(18):8823. doi: 10.3390/ijms26188823 (PMC12469465; doi:10.3390/ijms26188823)
Supplement: Supplementary file 1 [file ijms-26-08823-s001.zip › supplementary files/IJMS-3803115_Figure S4.pdf]

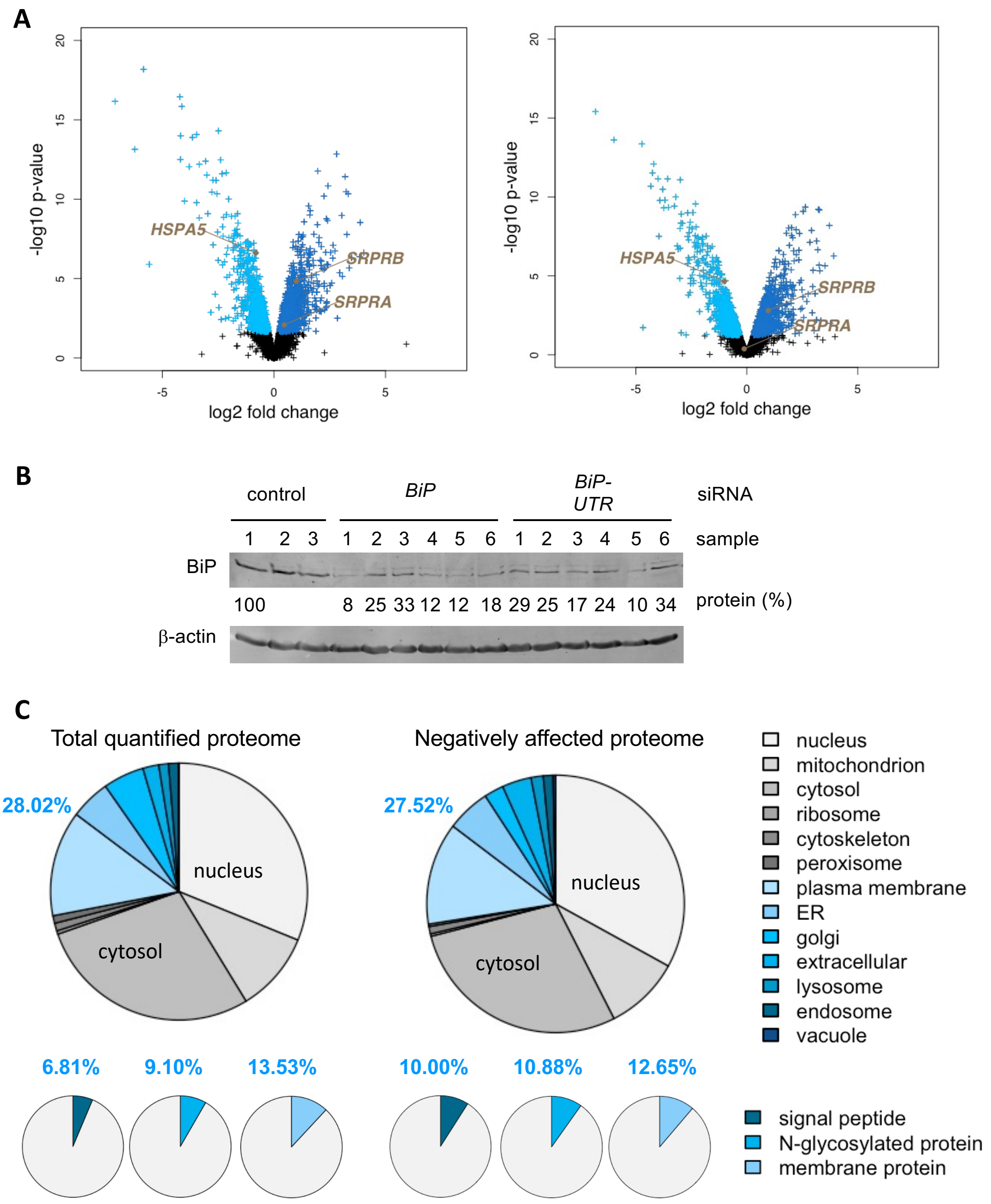

Figure S4. Identification of BiP clients and compensatory mechanisms by knock-down of BiP in HeLa cells. (**A**) The experimental strategy included siRNA-mediated gene silencing using two different siRNAs for the target and one non-targeting (control) siRNA for 72 h with three replicates for each siRNA, label-free quantitative proteomic analysis, and differential protein abundance analysis to identify negatively affected proteins (i.e. clients) and positively affected proteins (i.e. compensatory mechanisms). Original data are shown in Tables S9-12 [94]. (**B**) Knockdown efficiencies were evaluated by Western blot. (**C**) Differentially affected proteins were characterized by the mean difference of their intensities plotted against the respective permutation false discovery rate-adjusted p-values in volcano plots (n=1). The results for single siRNAs are shown in A. For validation of clients, protein annotations of signal peptides, membrane location, and N-glycosylation in humans were extracted from UniProtKB, and used to determine the enrichment of Gene Ontology (GO) annotations among the secondarily affected proteins. The colors of GO annotations (large pies) and the three others (small pies) are indicated in the Figure. Statistical analysis was carried out as described previously [94,200].
