# Supplementary material for: Rules of Engagement for Components of Membrane Protein Biogenesis at the Human Endoplasmic Reticulum
Source: Int J Mol Sci. 2025 Sep 10;26(18):8823. doi: 10.3390/ijms26188823 (PMC12469465; doi:10.3390/ijms26188823)
Supplement: Supplementary file 1 [file ijms-26-08823-s001.zip › supplementary files/IJMS-3803115_Figure S6.pdf]

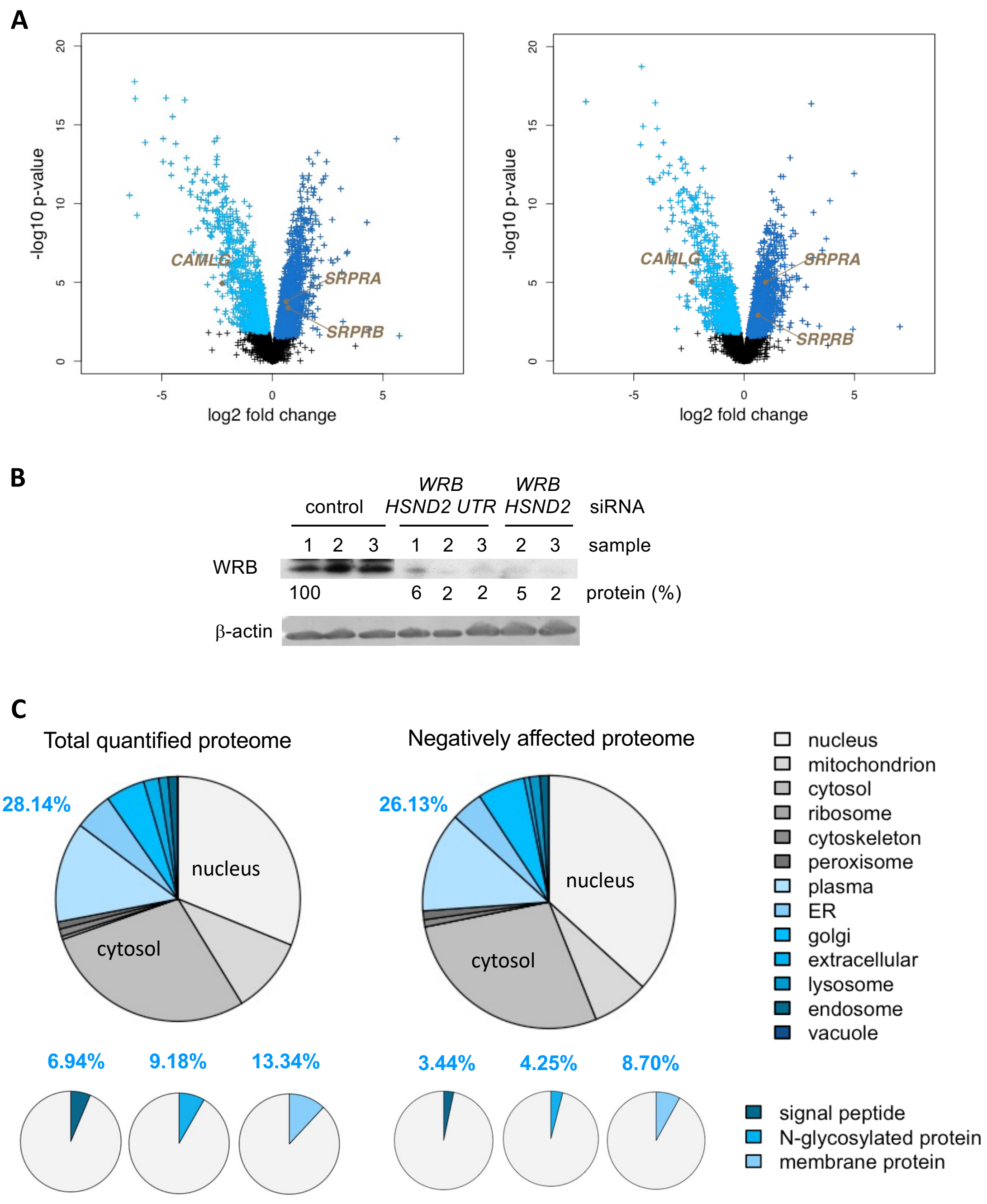

Figure S6. Identification of Wrb- and Snd2-clients and compensatory mechanisms by knock-down of Wrb and Snd2 in HeLa cells. (**A**) The experimental strategy included siRNA-mediated gene silencing using two different siRNAs for Snd2 in combination with a single siRNA for Wrb or one non-targeting (control) siRNA for 96 h with three replicates, label-free quantitative proteomic analysis, and differential protein abundance analysis to identify negatively affected proteins and positively affected proteins. Original data are shown in Tables S13-16. (**B**) Knockdown efficiencies were evaluated by Western blot. (**C**) Differentially affected proteins were characterized by the mean difference of their intensities plotted against the respective permutation false discovery rate-adjusted p-values in volcano plots (n=1). The results for single siRNA combinations are shown in A. For validation of clients, protein annotations of signal peptides, membrane location, and N-glycosylation in humans were extracted from UniProtKB, and used to determine the enrichment of Gene Ontology (GO) annotations among the secondarily affected proteins. The colors of GO annotations (large pies) and the three others (small pies) are indicated in the Figure. Statistical analysis was carried out as described previously [94,200].
