# Supplementary material for: Rules of Engagement for Components of Membrane Protein Biogenesis at the Human Endoplasmic Reticulum
Source: Int J Mol Sci. 2025 Sep 10;26(18):8823. doi: 10.3390/ijms26188823 (PMC12469465; doi:10.3390/ijms26188823)
Supplement: Supplementary file 1 [file ijms-26-08823-s001.zip › supplementary files/IJMS-3803115_Figure S7.pdf]

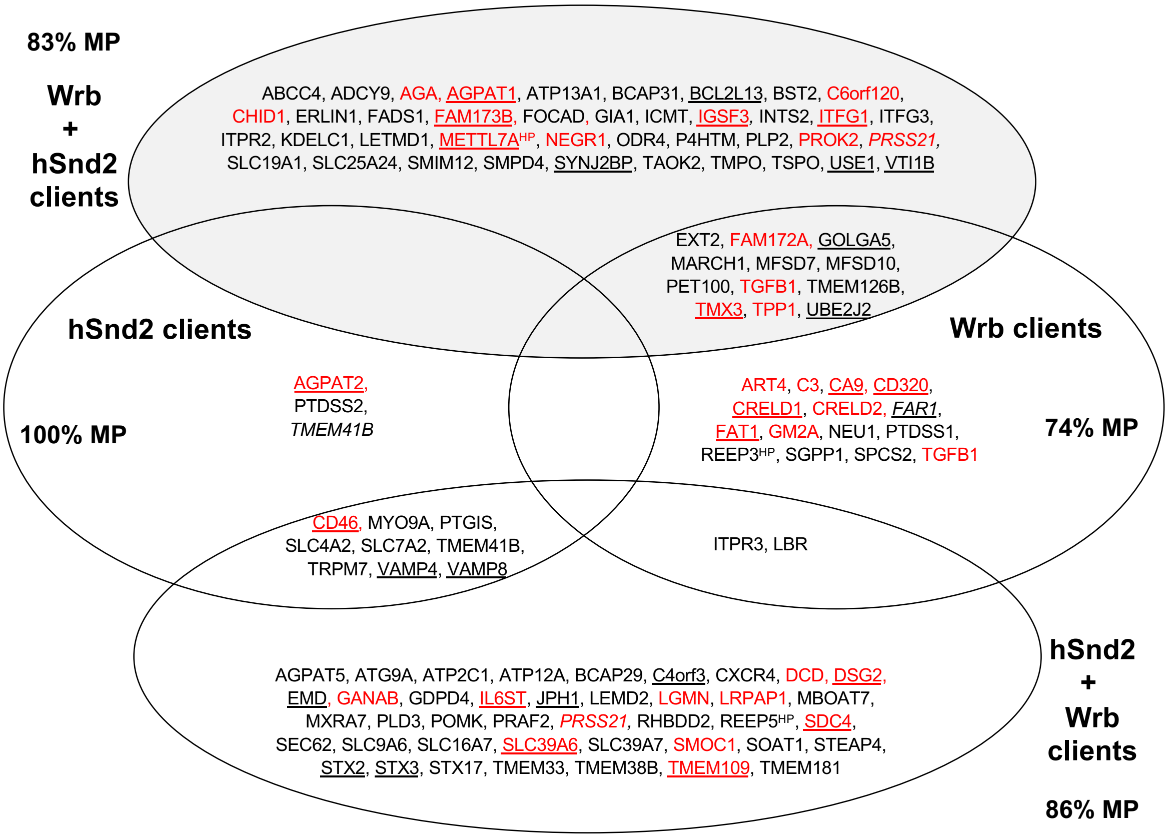

Figure S7. Venn diagram for the clients of two components for protein targeting to the human ER. The clients were determined by quantitative MS and differential protein abundance analysis following depletion of the respective component in HeLa cell for 96 h. Clients are defined as such by the presence of either an SP or at least one TMH. Shown with their gene names, clients with SPs are shown in red, SP containing membrane proteins are underlined, clients with TMH are shown in black, TA membrane proteins are underlined, hairpin proteins are indicated by superscript HP, and italics highlight clients of two targeting components, which could not be properly fitted into the Venn diagram and are named twice. With the exception of the concurrent WRB + hSND2 double silencing (shown in grey), the data were previously reported by Tirincsi et al., 2022 [174].
