# Supplementary material for: Rules of Engagement for Components of Membrane Protein Biogenesis at the Human Endoplasmic Reticulum
Source: Int J Mol Sci. 2025 Sep 10;26(18):8823. doi: 10.3390/ijms26188823 (PMC12469465; doi:10.3390/ijms26188823)
Supplement: Supplementary file 1 [file ijms-26-08823-s001.zip › supplementary files/IJMS_Table S3.pdf]

Table S3. Receptor clients as determined by MS and differential protein abundance analysis.

Clients of	Clients with			Clients with				
	SP	SP/ss	SP/ms	TMH	TMH/ss	TMH/ms	HP	TA
RRBP1	**22**	**6**	**-**	**17**	**7**	**8**	**2**	**-**
Total	*39*							
% of total	*56.4 SP*			*43.6 TMH*				
% of total	*59.0 MP*							
% of TMH					*41.2*	*47.1*	*11.8*	*-*
% of total	*41.0sol*	*15.4*	*-*		*17.9*	*21.0*	*5.1*	*-*
% of MP		*26.1*			*30.4*	*34.8*	*8.7*	*-*
KTN1	**3**	**-**	**1**	**8**	**4**	**3**	**-**	**1**
Total	*11*							
% of total	*27.3 SP*			*72.7 TMH*				
% of total	*81.8 MP*							
% of TMH					*50.0*	*37.5*	*-*	*12.5*
% of total	*18.2sol*	*-*	*9.1*		*36.4*	*27.3*	*-*	*9.1*
% of MP		*-*	*11.1*		*44.4*	*33.3*	*-*	*11.1*
ERj1	**6**	**1**	**1**	**9**	**4**	**4**	**-**	**1**
Total	*15*							
% of total	*40.0 SP*			*60.0 TMH*				
% of total	*73.3 MP*							
% of TMH					*44.4*	*44.4*	*-*	*11.1*
% of total	*26.7sol*	*6.7*	*6.7*		*26.7*	*26.7*	*-*	*6.7*
% of MP		*9.1*	*9.1*		*36.4*	*36.4*	*-*	*9.1*
SR	**24**	**14**	**-**	**32**	**11**	**18**	**3**	**-**
Total	*56*							
% of total	*42.9 SP*			*57.1 TMH*				
% of total	*82.1 MP*							
% of TMH					*33.3*	*56.3*	*9.4*	*-*
% of total	*17.9sol*	*25.0*	*-*		*19.6*	*32.1*	*5.4*	*-*
% of MP		*30.4*	*-*	*69.6*	*23.9*	*39.1*	*6.5*	*-*
Clients with N-terminal topogenic sequences in % of MP					*80*			
Clients with N-terminal topogenic sequences in % of total					*84*			
Snd2	**16**	**8**	**2**	**41**	**6**	**27**	**1**	**7**
Total	*57*							
% of total	*28.1 SP*			*71.9 TMH*				
% of total	*89.5 MP*							
% of TMH					*14.6*	*65.9*	*2.4*	*17.1*
% of total	*10.5sol*	*14.0*	*3.5*		*10.5*	*47.4*	*1.8*	*12.3*
% of MP		*15.7*	*3.9*	*80*	*11.8*	*52.9*	*2.0*	*13.7*
Clients with N-terminal topogenic sequences in % of MP					*65*			
Clients with N-terminal topogenic sequences in % of total					*68*			

Wrb	***26***	***11***	***2***	***47***	***6***	***31***	***2***	***8***
Total	*73*							
% of total	*35.6 SP*			*64.4 TMH*				
% of total	*82.2 MP*							
% of TMH					*12.8*	*66.0*	*4.3*	*17.0*
% of total	*17.8sol*	*13.9*	*2.5*		*8.2*	*42.5*	*2.7*	*11.0*
% of MP		*18.3*	*3.3*	*78*	*10*	*51.7*	*3.3*	*13.3*
Clients with N-terminal topogenic sequences in % of MP					*57*			
Clients with N-terminal topogenic sequences in % of total					*67*			
PEX3	***28***	***5***	***1***	***23***	***8***	***7***	***2***	***6***
Total	*51*							
% of total	*54.9 SP*			*45.1 TMH*				
% of total	*56.8 MP*							
% of TMH					*34.8*	*30.4*	*8.7*	*26.1*
% of total	*43.1sol*	*9.8*	*2*		*15.7*	*13.7*	*3.9*	*11.8*
% of MP		*17.2*	*3.4*	*79*	*27.6*	*24.1*	*6.9*	*20.7*
Clients with N-terminal topogenic sequences in % of MP					*55*			
Clients with N-terminal topogenic sequences in % of total					*78*			

HP, hairpin proteins; MP, membrane proteins; ms, multispanning membrane proteins; sol, soluble proteins; SP, signal peptide-containing proteins; ss, single-spanning membrane proteins; TA, tail anchor proteins; TMH, membrane proteins with transmembrane helix that serves as SP-equivalent; Notably, Wrb and Snd2, respectively, refers to the pool of clients that were detected after the respective single depletion (Snd2 or Wrb) plus the double depletion (Snd2+Wrb); PEX3 refers to the pool of clients that were detected after PEX3 depletion in HeLa cells and in the Zellweger patient fibroblasts with a PEX3 deficiency. The Table was updated for putative membrane protein clients that have their functional location in lipid droplets, peroxisomes, or mitochondria.
