# Supplementary material for: Rules of Engagement for Components of Membrane Protein Biogenesis at the Human Endoplasmic Reticulum
Source: Int J Mol Sci. 2025 Sep 10;26(18):8823. doi: 10.3390/ijms26188823 (PMC12469465; doi:10.3390/ijms26188823)
Supplement: Supplementary file 1 [file ijms-26-08823-s001.zip › supplementary files/IJMS_Table S2.pdf]

Table S2. Quantitative MS data were deposited to the ProteomeXchange Consortium via the PRIDE partner repository with the indicated dataset identifiers (http://www.proteomexchange.org)

**MS run**<br>**Sample number**	**Project title**<br>**Sample description**	**Dataset identifier**	**References**
**Orbi1763**	**Clients of human TRAP complex**	**PXD008178**	
Sample 1-3	scr control siRNA		Nguyen et al., 2018
Sample 4-6	SEC61A1 siRNA #4		Nguyen et al., 2018
Sample 7-9	SEC61A1 siRNA #5		Nguyen et al., 2018
**Orbi1931**	**Clients of human TRAP complex**	**PXD008178**	
Sample 1-3	scr control siRNA		
Sample 4-6	SEC63 siRNA #5		Schorr et al., 2019
Sample 7-9	SEC63 siRNA #7		Schorr et al., 2019
Sample 10-12	TRAPB siRNA #2		Nguyen et al., 2018
Sample 13-15	TRAPB siRNA #3		Nguyen et al., 2018
Sample 16-18	scr control siRNA		
Sample 19-21	SEC62 siRNA #2		Schorr et al., 2019
Sample 22-24	SEC62 siRNA UTR		Schorr et al., 2019
Sample 25-27	TRAM siRNA #2		Klein et al., 2020
Sample 28-30	TRAM siRNA #6		Klein et al., 2020
**Orbi1986**	**Clients of human TRAP complex**	**PXD008178**	
Sample 1-3	scr control siRNA		
Sample 4-6	SRA siRNA #3		Tirincsi et al., 2022
Sample 7-9	SRA siRNA #6		Tirincsi et al., 2022
Sample 10-12	TRAP siRNA #2		Nguyen et al., 2018
Sample 13-15	TRAP siRNA #3		Nguyen et al., 2018
**Orbi2048**	**Targeting of precursor polypeptides to the human ER**	**PXD008178**	
Sample 1-3	scr control siRNA		
Sample 4-6	BIP siRNA CA		Schorr et al., Supplement
Sample 7-9	BIP siRNA UTR#1		Schorr et al., Supplement
Sample 10-12	SEC61A1 siRNA #4		Nguyen et al., 2018
Sample 13-15	SEC61A1 siRNA #5		Nguyen et al., 2018
Sample 16-18	scr control siRNA		
Sample 19-21	WRB siRNA #3		Tirincsi et al., 2022
Sample 22-24	WRB siRNA #4		Tirincsi et al., 2022
Sample 25-27	WRB siRNA #4 + SND2 siRNA #2 = WS		this study
Sample 28-30	WRB siRNA #4 + SND2 siRNA #3 = WS		this study
Sample 31	control fibroblasts #1		Nguyen et al., 2018
Sample 32	control fibroblasts #2		Nguyen et al., 2018
Sample 33	CDG patient fibroblasts @SSR3		Nguyen et al., 2018
Sample 34	CDG patient fibroblasts @SSR4 #1		Nguyen et al., 2018
Sample 35	CDG patient fibroblasts @SSR4 #2		Nguyen et al., 2018
**Orbi2085**	**Clients of human TRAP complex**	**PXD008178**	
Sample 1-3	scr control siRNA		
Sample 4-6	TRAP siRNA #2		Nguyen et al., 2018
Sample 7-9	TRAP siRNA #3		Nguyen et al., 2018
Sample 10-12	TRAM siRNA #2		Klein et al., 2020
Sample 13-15	TRAM siRNA #6		Klein et al., 2020
**Orbi2155**	**Clients of human Sec62/Sec63 complex**	**PXD012078**	
Sample 1-3	scr control siRNA		
Sample 4-6	SEC62 siRNA #2		Schorr et al., 2019
Sample 7-9	SEC62 UTR siRNA		Schorr et al., 2019
Sample 10-12	SRA siRNA #3		Tirincsi et al., 2022
Sample 13-15	SRA siRNA #6		Tirincsi et al., 2022

MS run<br>Sample number	Project title<br>Sample description	Dataset identifier	Reference
**Orbi2288**	**mRNA targeting to the human ER**	**PXD011989**	
Sample 1-3	scr control siRNA		Bhadra et al., 2021
Sample 4-6	P180 siRNA #1		Bhadra et al., 2021
Sample 7-9	P180 siRNA #2		Bhadra et al., 2021
**Orbi2314**	**mRNA targeting to the human ER**	**PXD011990**	Bhadra et al., 2021
Sample 1-3	scr control siRNA		
Sample 4-6	KTN1 siRNA #3		Bhadra et al., 2021
Sample 7-9	KTN1 siRNA #4		Bhadra et al., 2021
Sample 10-12	ERJ1 UTR siRNA #4		Bhadra et al., 2021
Sample 13-15	ERJ1 siRNA #6		Bhadra et al., 2021
**Orbi2514**	**Targeting of precursor polypeptides to the human ER**	**PXD011993**	
Sample 1-3	scr control siRNA		
Sample 4-6	SND2 siRNA #2		Tirincsi et al., 2022
Sample 7-9	SND2 siRNA #3		Tirincsi et al., 2022
Sample 10-12	WRB siRNA #3 + SND2 siRNA #2 = SW		Tirincsi et al., 2022
Sample 13-15	WRB siRNA #3 + SND2 siRNA #3 = SW		Tirincsi et al., 2022
Sample 16-18	HEK293 Mock CRISPR		Schorr et al., 2019
Sample 19-21	HEK293 SEC62 CRISPR		Schorr et al., 2019
Sample 22-24	HEK293 SEC63 CRISPR		Schorr et al., 2019
**Orbi2695**	**Targeting of proteins to the human ER**	**PXD12005**	
Sample 1-3	scr control siRNA		Zimmermann et al., 2021
Sample 4-6	PEX3 siRNA #1		Zimmermann et al., 2021
Sample 7-9	PEX3 siRNA #2		Zimmermann et al., 2021
Sample 10-12	control fibroblasts #3		Zimmermann et al., 2021
Sample 13-15	Zellweger patient fibroblasts ◎PEX3		Zimmermann et al., 2021

We note that the siRNA numbering may be different from the siRNA numbering in the respective reference and that in some siRNA experiments the scrambled (scr) control siRNA served as negative control for several targets and, possibly, different references. Therefore, in these latter cases no reference is given.
