# Supplementary material for: Rules of Engagement for Components of Membrane Protein Biogenesis at the Human Endoplasmic Reticulum
Source: Int J Mol Sci. 2025 Sep 10;26(18):8823. doi: 10.3390/ijms26188823 (PMC12469465; doi:10.3390/ijms26188823)
Supplement: Supplementary file 1 [file ijms-26-08823-s001.zip › supplementary files/IJMS_Table S1.pdf]

Table S1. Components/*complexes* for membrane protein biogenesis at the ER and linked diseases.

Component/subunit	Abundance [1]	Location [2]	Linked Diseases
**80S ribosomes**	1500	C	
**for N-terminal modification**			
NAC [3,4]		C	
- NACα	1412		Dermatitis
- NACβ			Scleral Staphyloma
METAP1 [3]	203	C	Microsporidosis
METAP2 [3]	346	C	Microsporidosis
NatA [3]		C	Ogden syndrome
- Naa10 [5]	821		
- Naa15	374		
- Naa16	35		
NatB [3]		C	Intellectual Develompental Disorder
- Naa20 [5]	45		
- Naa25	130		
NatC [3]		C	Microphthalmia
- Naa30 [5]	27		
- Naa35	20		
- Naa38			
NatD (Naa40) [5,6]		C	Pulmonary Subvalvular Stenosis
NatE [3]		C	Ogden Syndrome, Chops Syndrome
- Naa10 [5]	821		
- Naa15	374		
- Naa50	146		
NatF (Naa60)		C	Basal Ganglia Calcification
**for mRNA or ribosome targeting**			
AEG-1 (LYRIC, MTDH)	575	ERM	GBM, Hepatocellular Carcinoma
KTN1 (Kinectin 1)	263	ERM	Hepatocellular Carcinoma
LRRC59 [3] (LRC59, p34)	2480	ERM	Ogden Syndrome
RRBP1 [3] (p180)	135	ERM	
**for precursor targeting**			
SRP [3]		C	
- SRP72	355		Aplasia, Myelodysplasia
- SRP68	197		
- SRP54	228		NP, Pancreas Insufficiency
- SRP19	33		NP
- SRP14	4295		Prostate Cancer
- SRP9	3436		Hepatocellular & Colorectal Carcinoma
- 7SL RNA			Breast-, Liver-, Lung-, Stomach-Cancer
SRP receptor		ERM	
- SRα (docking protein)	249		NP
- SRβ	173		Testis Seminoma
Calmodulin	9428	C	
hSnd1			
Snd receptor		ERM	
- hSnd2 (TMEM208)	81		CVID, Developmental Delay
- TMEM109 [7]	49		NP
PEX19	80	C	Zellweger Syndrome
PEX3	103	ERM,PexM	Zellweger Syndrome
PEX16	9	ERM,PexM	Zellweger Syndrome
**for precursor targeting and membrane insertion**			
Bag6 complex [3]		C	
- TRC35 (Get4)	171		CDG, PLD
- Ubl4A (Get5)	177		Rett Syndrome
- Bag6 (Bat3)	133		Retinits Pigmentosa, Lung Cancer
SGTA [3]	549	C	Breast Cancer, Lung Cancer
TRC40 (Asna1, Get3)	381	C	CDG, Zellweger Syndrome

TA receptor and insertase		ERM	
- CAML (CAMLG, Get2)	5		CDG, CVID, PKD
- WRB [8] (CHD5, Get1)	4		Congenital Heart Disease
**for membrane insertion**			
EMC		ERM	
- EMC1	124		Visual Disorders
- EMC2	300		
- EMC3 [8]	270		
- EMC4	70		
- EMC5 (MMGT1)	35		
- EMC6 (TMEM93)	5		
- EMC7	247		
- EMC8	209		
- EMC9	1		
- EMC10	3		Developmental Delay
GEL complex [3]		ERM	Glaucoma, Cerebrofaciothoracic Dysplasia
- TMCO1 [7,8]	2013		
- OPTI (RAB5IF)	5		
- CCDC47 (Calumin)	193		
BOS complex [3]		ERM	
- NCLN (Nicalin)	99		Hirschsprung Disease
- TMEM147	21		Cystinosis
- NOMO 1/2	267		Pseudoxanthoma Elasticum
PAT complex [3]		ERM	
- PAT10 (Asterix)			
- CCDC47 (Calumin)	193		
**for membrane insertion and translocation**			
Sec61 complex [3,7]		ERM	Buruli Ulcer, Pneumonia, Sepsis
- Sec61α1	139		CVID, NP, TKD,
- Sec61β	456		NP, PLD, Colorectal Cancer
- Sec61γ	400		GBM, Hepatocellular Carcinoma, NP
Sec62/63 complex [3]		ERM	
- Sec62 (TLOC1)	26		Breast-, Prostate-, Cervix-, Lung-Cancer, NP
- Sec63 (ERj2)	168		CDG, NP, PLD, Colorectal Cancer
ERj1 [3] (DNAJC1)	8	ERM	GBM, Pyometritis
TRAM1 [3]	26	ERM	Pancreatitis
TRAM2	40	ERM	PKD
TRAP complex [3]		ERM	
- TRAPα (SSR1)	568		NP
- TRAPβ (SSR2)			
- TRAPγ (SSR3)	1701		CDG, NP, Hepatocellular Carcinoma
- TRAPδ (SSR4)	3212		CDG
RAMP4 [3] (SERP1)		ERM	Arachnoiditis
**for folding and assembly**			
ER chaperones			
- BiP (Grp78, HSPA5)	8253	ERL	HUS
- Calreticulin (CaBP3, ERp60)	14521	ERL	
- Calnexin$_{palmitoylated}$ [3]	7278	ERM	CDG
- ERj3 (DNAJB11)	1001	ERL	PKD, PLD
- ERj4 (DNAJB9)	12	ERL	
- ERj5 (DNAJC10)	43	ERL	
- ERj6 (DNAJC3, p58^{IPK})	237	ERL	Diabetes, MSS, Neurodegeneration, NP
- ERj7 (DNAJC25)	10	ERM	Hyperinsulinismus, Allergic Asthma
- ERj8 (DNAJC16)	24	ERM	Prostatic Cyst
- ERj9 (DNAJC22)		ERM	
- Grp94 (CaBP4, Hsp90B1)	4141	ERL	
- Grp170 (HYOU1)	923	ERL	
- Sil1 (BAP)	149	ERL	MSS

Folding enzymes			
- PDI (PDIA1, P4HB))	3625	ERL	Cole-Carpenter Syndrome
- PDIp (PDIA2)		ERL	Immune Deficiency Disease
- ERp57 (Grp58, PDIA3)	2543	ERL	Gastric Cancer
- CypB (PPIB)	1291	ERL	Brittle Bone Disorder
- FKBP13 (FKBP2)	894	ERL	Ehlers-Danlos Syndrome
**for covalent modification**			
Oligosaccharyltransferase A [3] *(OST-A)*		ERM	
- RibophorinI (Rpn1)	1956		
- RibophorinII (Rpn2)	527		
- OST48 (DDOST)	273		CDG
- Dad1	464		
- OST4			NP
- TMEM258			
- Stt3A[5]	430		CDG
- DC2 (OSTC)			CDG
- Kcp2			
Oligosaccharyltransferase B (OST-B)			
- RibophorinI (Rpn1)	1956		
- RibophorinII (Rpn2)	527		
- OST48	273		CDG
- Dad1	464		
- OST4			NP
- TMEM258			
- Stt3B [5]	150		CDG
- TUSC3			CDG
- MagT1 (OST3B)	33		CDG
Signal peptidase complex A (SPC-A)		ERM	
- SPC12 (SPCS1)	2733		
- SPC18 [5] (SEC11A)			Gastric Cancer
- SPC22/23 (SPCS3)	334		Glycogen Storage Disease
- SPC25 (SPCS2)	94		
Signal peptidase complex C (SPC-C)		ERM	
- SPC12	2733		
- SPC21 [5] (SEC11C)			Polyposis Syndrome
- SPC22/23 (SPCS3)	334		Glycogen Storage Disease
- SPC25 (SPCS2)	94		
GPI transamidase (GPI-T)		ERM	
- GPAA1	9		Epilepsy
- PIG-K [5]	38		CDG, Epilepsy
- PIG-S	86		CDG
- PIG-T	20		CDG, Epilepsy
- PIG-U	42		CDG, Epilepsy

[1] Abundance refers to the concentration (nM) of the respective protein in HeLa cells (Hein, M.Y. et al., 2015).
[2] Localization refers to the intracellular localization(s): C, Cytosol, ERL, ER lumen, ERM, ER membrane, PexM, Peroxisome membrane. [3] ribosome association. [4] Complexes are given by italics. [5] catalytic activity.
[6] Alternative protein names are given in parentheses. [7] ion channel activity. [8] Oxa1-like insertase activity.
- Abbreviations for protein names: BOS, Back of Sec61 complex, Cyp, cyclophilin, EMC, ER membrane complex, ERj, ER J-domain protein, FKBP, FK506 binding protein, GEL, GET- and EMC-like, GET, Guided entry of tail-anchored proteins, GPI, Glycosylphosphatidylinositol, METAP, Methionine aminopeptidase, NAC, Nascent polypeptide-associated complex, NAT, N-acetyltransferase, OST, Oligosaccharyltransferase, PDI, Protein disulfide isomerase, PPI, Peptidyl-prolyl cis/trans isomerase, SEC, Protein involved in secretion, SND, SRP-independent, SPC, Signal peptidase complex, SR, SRP receptor, SRP, Signal recognition particle, SSR, Signal sequence receptor, TA, Tail anchor, TMEM, Transmembrane (protein), TRAM, translocating chain-associating membrane, TRAP, Translocon-associated protein, TRC, Transmembrane recognition complex.
- Abbreviation for diseases: CDG, Congenital disorder of glycosylation, CVID, Common variable immunodeficiency, GBM, Glioblastoma multiforme, HUS, Hemolytic-uremic syndrome, MSS, Marinesco-Sjögren syndrome, NP, Severe combined congenital neutropenia, PKD, Polycystic kidney disease, PLD, Polycystic liver disease, TKD, Tubulointerstitial kidney disease.
