# Supplementary material for: Rules of Engagement for Components of Membrane Protein Biogenesis at the Human Endoplasmic Reticulum
Source: Int J Mol Sci. 2025 Sep 10;26(18):8823. doi: 10.3390/ijms26188823 (PMC12469465; doi:10.3390/ijms26188823)
Supplement: Supplementary file 1 [file ijms-26-08823-s001.zip › supplementary files/IJMS_Table S6.pdf]

Table S6. Characteristics of THM-containing clients of mRNA- or protein-targeting components RRBP1, KTN1, SR, Snd, Wrb and PEX3.

Protein	TMDs	Type	TMH	TMH Sequence	Size	NG	LD	ΔM	NAT
**RRBP1**									
NCEH1	1	II	5-25	CVLLTALVALAAYYVYIPLPG	408	3	1		
SPTLC1	1	III	16-36	ALYEAPAYHLILEGILILWII	513				
CYP51A1	1		24-44	GNLLSMLLIACAFTLSLVYLI	509				
CD151	4		19-39	LLFTYNCCFWLAGLAVMAVGI	253	1			
GPRC5A	7	III	34-54	VATAGVVTSVAFMLTLPILVC	357	1			
APMAP	1	II	41-61	VTFLMLAVSLTVPLLGAMMLL	416	2	1	ΔM	NAT
SLC38A5	11	II	49-71	SFGMSVFNLSNAIMGSGILGL	472	1	1		NAT
DPY19L1	11	II	92-108	LYYSYFKTIVEAPSFLNGVWMIM	748		3		
DEGS1	6	II	41-61	PNLIWIIIMMVLTQLGAFYIV	323		1		
SOAT1	9	II	141-159	IYHMFIALLILFILSTLVV	550		1		NAT
PARL	7	II	102-121	LYYSYFKTIVEAPSFLNGVWMIM	379				
SLC3A2	1	II	185-205	LLLFWLGWLGMLAGAVVIIVR	630	4	1	ΔM	NAT
SUN2	1	II	213-233	FLWFLLPLLLLTCLTYGAWYF	717	1	1		
FADS2	4	II	132-152	FFLLLLAHIIALESIAWFTVF	444		1		
TIMM21 [M]	1		108-128	FTYLIVVLFGISITGGLFYTI	248				
ATL2	1	HP	477-497	TLFAVMFAMYIISGLTGFIGL	583				
ATL3	1	HP	446-466	VLFTGIVALYIASGLTGFIGL	541				
17									
**KTN1**									
GALT4	1	II	13-35	LLLAFLTVAYIFVELLVSTFHAS	578	1	1		
QPCTL	1	II	35-55	LLPLLLALAVGSAFYTIWSGW	382		1		
ATP6V0C	4	III	11-33	ASFFAVMGASAAMVFSALGAAYG	155				
PTPLB	6	II	42-60	LVIYNVVMTAGWLVIAVGL	254	1		ΔM	NAT
TMC1	6	II	200-220	MVLFILTFSLIMLPEYLWGLP	760		2		
AVL9	1		214-230	LVGALMTVLSLFPGMIE	648				
TMEM106B	1	II	97-117	YVMASVFVCLLLSGLAVFFLF	247	5	1	ΔM	
BCL2L1	1	TA	210-226	FNRWFLTGMTVAGVVLL	233				
8									
**ERJ1**									
GALT4	1	II	13-35	LLLAFLTVAYIFVELLVSTFHAS	578	1	1		
COMT	1	II	7-26	LLLAAVLLGLVLLVVLLLLL	271		1		
NEU1	1		20-41	LGFWGGCRVWVFAAIFLLLSLAA	415	3			
QPCTL	1	II	35-55	LLPLLLALAVGSAFYTIWSGW	382		1		
ATP6V0C	4	III	11-33	ASFFAVMGASAAMVFSALGAAYG	155				
PTPLB	6	II	42-60	LVIYNVVMTAGWLVIAVGL	254	1		ΔM	NAT
SOAT1	9	II	141-159	IYHMFIALLILFILSTLVV	550		1		NAT
TMEM131	2	II	1091-1111	FVFILNASLPYHMLATCAEAL	1883	1	1		
BCL2L1	1	TA	210-226	FNRWFLTGMTVAGVVLL	233				
9									
**SR**									
TMTC3	9	II	9-29	ITLIVGVVTACYWNSLFCGFV	914	3			
B3GALTL	1	II	7-27	WWLLAPPALLALLTCSLAFGL	498	1	1		
SLC35B2	9		5-25	WWAVVVLAAFPSLGAGGETPE	432				
ERLIN2	1	II	4-24	LGAVVAVASSFFCASLFSAVH	339	1	1		
REEP3	2	HP	1-21	MVSWMISRAVVLVFGMLYPAY	255				
SPTLC1	1		16-36	ALYEAPAYHLILEGILILWII	513				
SLC16A3	12	II	18-38	GGWGWAVLFGCFVITGFSYAF	465		1		
ZMPSTE24	7	III	19-39	IFGAVLLFSWTVYLWETFLAQ	475				
PDE3A	6	II	61-81	LSSALCAGSLSFLLALLVRLV	1141		1		
ATP13A1	10	II	67-87	VLPFAGLLYPAWLGAAAAGCW	1204	2		ΔM	NAT
TMEM209	2	II	28-48	VVLAWGLLNVSMAGMIYTEMT	561	2			
PEX3 [P]	2		16-36	CIFLGTVLGGVYILGKYGQKK	373				
ABCC4	13	II	93-113	LVLGIFTLIEESAKVIQPIFL	1325	7	2		
ASPH	1	II	54-74	FFTWFMVIALLGVWTSVAVVW	758	2	1		
ATP2B1	10	II	98-118	FLQLVWEALQDVTLIILEIAA	1220			ΔM	NAT
ERGIC2	2	II	34-54	GTVSLIAFTTMALLTIMEFSV	377				
LNPEP	1	II	111-131	MVVCAFVIVVAVSVIMVIYLL	1025	18	1		NAT
IKBIP	1		46-62	CLSLLSLGTCLGLAWFV	377	2			

DEGS1	6	II	41-61	PNLIWIIIMMVLTQLGAFYIV	323				
BST2	1	II	21-48	KLLLGIGILVLLIIVILGVPLIIFTIKA	180	2	1		
TMEM41B	6	II	52-72	MSLLILVSIFLSAAFVMFLVY	291				
TVP23B	4	II	34-53	PVASFFHLFFRVSAIIVYLL	205			ΔM	NAT
CEPT1	10	II	87-107	LITIIGLSINICTTILLVFYC	416	1			
PEX14 [P]	1		109-126	YGALAIIMAGIAFGFHQL	377			ΔM	NAT
ANO10	8	II	208-228	IALYFGFLEYFTFALIPMAVI	660				
SUN1	1	II	316-335	ICKFLVLLIPLFLLLAGLSL	785		1		
TOR1AIP2	1		215-235	FWSYGPVILVVLVVAVVASSV	470	2		ΔM	NAT
SLC25A24 [M]	6		198-215	LLAGGIAGAVSRTSTAPL	427				
YIPF5	5	II	125-145	TDLAGPMVFCLAFGATLLLAG	257				
CAV1	1	HP	105-125	ALFGIPMALIWGIYFAILSFL	178			ΔM	NAT
ITPR3	6	II	2203-2223	LWGSISFNLAVFINIIIAFFY	2671				
ATL2	1	HP	477-497	TLFAVMFAMYIISGLTGFIGL	583				
32									
**Snd2**									
PTGIS	1		1-20	MAWAALLGLLAALLLLLLLS	500				
MBOAT7	7	III	9-29	LVVLLISIPIGFLFKKAGPGL	472	1			
SLC39A7	6	II	10-30	WVAVGLLTWATLGLLVAGLGG	469				
SLC16A7	12	II	16-36	GGWGWIVVGAAFISIGFSYAF	478				
GDPD4	6	II	18-38	WVTFLGTGYWFFWSIFILSLA	520	2	1		
SLC9A6	13		28-48	LWLLLAVGVFDWAGASDGGGG	679	1			
RHBDD2	5		11-31	WCLCPEVPSATFFTALLSLLV	364				
AGPAT5	3		15-35	LLPSVVLLGTAPTYVLAWGVW	364				
BCAP29	3	III	7-27	AVATFLYAEIGLILIFCLPFI	241				
MYO9A	1		175-195	IYTYVGSILIVINPFKFLPIY	2548				
SLC7A2	14	II	38-59	DLIALGVGSTLGAGVYVLAGEV	658	3			
ATP2C1	10	II	71-91	LWKKYISQFKNPLIMLLLASA	919				
POMK	1	II	21-43	VGLLLIMALMNTLLYLCLDHFFI	350	3	1		NAT
ATG9A	6	II	67-87	IFELMQFLFVVAFTTFLVSCV	839	1		ΔM	NAT
MXRA7	1		7-27	LLAALPALATALALLLAWLLV	170		1		
PLD3	1	II	39-59	VLLVLILAVVGFGALMTQLFL	490	2	1		
ATP12A	10	II	102-123	EIVKFLKQMVGGFSILLWVGA	1039		1		
ABCB8 [M]	5		86-102	SAWCWVGGALLGPMVLS	735				
CXCR4	7	II	39-63	IFLPTIYSIIFLTGIVGNGLVILVM	352	3			
PTDSS2	7	II	63-83	AHTLTVLFILTCTLGYVTLLE	487	1	1		
TMEM33	3	II	32-52	LFTVYCSALFVLPLLGLHEAA	247		1	ΔM	NAT
TMEM38B	4	III	50-70	SWFTAMLHCFGGGILSCLLLA	291		1		
TMEM41B	6		52-72	MSLLILVSIFLSAAFVMFLVY	291				
PET100 [M]	1		7-24	IFRMIIYLTFPVAMFWVS	73				
REEP5	2	HP	35-55	SFIALGVIGLVALYLVFGYGA	189				
SOAT1	9	II	141-159	IYHMFIALLILFILSTLVV	550				NAT
PRAF2	4	II	42-62	LYYQTNYLLCFGIGLALAGYV	178				
TMEM181	9	II	153-173	HFVLVFVVFFICFGLTIFVGI	475				
TRPM7	6	II	756-776	NSWYKVILSILVPPAILLLEY	1865		2		NAT
LEMD2	2	II	213-233	LLLWASLGLLLVFLGILWVKM	503		1	ΔM	NAT
STEAP4	6	II	196-216	LFPMWRFPFYLSAVLCVFLFF	459	1			
SEC62	2	II	197-217	FVMGLILVIAVIAATLFPLWP	399				
SLC4A2	10	II	708-731	CLAAVIFIYFAALSPAITFGGLLG	1241	3			
STX17	2		229-249	LAALPVAGALIGGMVGGPIGL	302			ΔM	NAT
C4orf3	1	TA	45-65	SYWLDLWLFILFDVVVFLFVYFL	65				
VAMP8	1	TA	76-96	MVLICVIVFIIILFIVLFAT	100				NAT
VAMP4	1	TA	116-136	IKAIMALVAAILLLVIIILIV	141				
EMD	1	TA	223-243	VPLWGQLLLFLVFVIVLFFIY	254				NAT
STX3	1	TA	264-284	LIIIIVLVVVLLGILALIIGLSV	289				
STX2	1	TA	265-288	WIIIAVSVVLVAIIALIIGLSVGK	288				
JPH1	1	TA	640-660	IMIVLVMLLNIGLAILFVHFL	661				
41									
**Wrb**									
MBOAT7	7	III	9-29	LVVLLISIPIGFLFKKAGPGL	472	1			
SLC39A7	6		10-30	WVAVGLLTWATLGLLVAGLGG	469				
REEP3	2	HP	1-21	MVSWMISRAVVLVFGMLYPAY	255				
EXT2	1	II	26-46	YITLFSIVLLGLIATGMFQFW	718	2	1		
SLC16A7	12	II	16-36	GGWGWIVVGAAFISIGFSYAF	478				

GDPD4	6	II	18-38	WVTFLGTGYWFFWSIFILSLA	520	2	1		
SLC9A6	13		28-48	LWLLLAVGVFDWAGASDGGGG	679	1			
RHBDD2	5		11-31	WCLCPEVPSATFFTALLSLLV	364				
AGPAT5	3		15-35	LLPSVVLLGTAPTYVLAWGVW	364				
BCAP29	3	III	7-27	AVATFLYAEIGLILIFCLPFI	241				
MFSD7	12	II	30-50	WVFLLAISLLNCSNATLWLSF	559				
NEU1	1		20-41	LGFWGGCRVWVFAAIFLLLSLAA	415	3			
MFSD10	11	II	27-47	VVFLGLLLDLLAFTLLLPLLP	455				
ATP2C1	10	II	71-91	LWKKYISQFKNPLIMLLLASA	919				
POMK	1	II	21-43	VGLLLIMALMNTLLYLCLDHFFI	350	3	1		NAT
ATG9A	6	II	67-87	IFELMQFLFVVAFTTFLVSCV	839	1		ΔM	NAT
PTDSS1	9	II	36-56	FFYRPHTITLLSFTIVSLMYF	473			ΔM	NAT
MXRA7	1		7-27	LLAALPALATALALLLAWLLV	170				
PLD3	1	II	39-59	VLLVLILAVVGFGALMTQLFL	490	2	1		
ATP12A	10	II	102-123	EIVKFLKQMVGGFSILLWVGA	1039		1		
ABCB8 [M]	5		86-102	SAWCWVGGALLGPMVLS	735				
CXCR4	7	III	39-63	IFLPTIYSIIFLTGIVGNGLVILVM	352	3			
TMEM33	3	II	32-52	LFTVYCSALFVLPLLGLHEAA	247		1	ΔM	NAT
TMEM38B	4	III	50-70	SWFTAMLHCFGGGILSCLLLA	291		1		
PET100 [M]	1		7-24	IFRMIIYLTFPVAMFWVS	73				
REEP5	2	HP	35-55	SFIALGVIGLVALYLVFGYGA	189				
SOAT1	9	II	141-159	IYHMFIALLILFILSTLVV	550				NAT
PRAF2	4	II	42-62	LYYQTNYLLCFGIGLALAGYV	178				
SGPP1	9	II	132-152	FCFGTELGNELFYILFFPFWI	441				
TMEM181	9	II	153-173	HFVLVFVVFFICFGLTIFVGI	475				
TMEM126B [M]	4		72-92	IYQMATFGTTAGFSGIFSNFL	230				
LBR	8	II	212-232	VPGVFLIMFGLPVFLFLLLLM	615				
SPCS2	2	II	87-107	ICTISCFFAIVALIWDYMHPF	226			ΔM	NAT
LEMD2	2	II	213-233	LLLWASLGLLLVFLGILWVKM	503		1	ΔM	NAT
STEAP4	6	II	196-216	LFPMWRFPFYLSAVLCVFLFF	459				
SEC62	2	II	197-217	FVMGLILVIAVIAATLFPLWP	399				
MARCH1	2	II	155-175	IFCSVTFHVIAITCVVWSLYV	289				
STX17	2		229-249	LAALPVAGALIGGMVGGPIGL	302			ΔM	NAT
ITPR3	2	II	2203-2223	LWGSISFNLAVFINIIIAFFY	2671		1		
C4orf3	1	TA	45-65	SYWLDLWLFILFDVVVFLFVYFL	65				
UBE2J2	1	TA	227-247	GLLGGALANLFVIVGFAAFAYTW	259				
EMD	1	TA	223-243	VPLWGQLLLFLVFVIVLFFIY	254				NAT
FAR1 [P]	1	TA	466-483	IRYGFNTILVILIWRIF	515				
STX3	1	TA	264-284	LIIIIVLVVVLLGILALIIGLSV	289				
STX2	1	TA	265-288	WIIIAVSVVLVAIIALIIGLSVGK	288				
GOLGA5	1	TA	699-719	VFVIIYMALLHLWVMIVLLTYTP	731			ΔM	NAT
JPH1	1	TA	640-660	IMIVLVMLLNIGLAILFVHFL	661				
47									
**PEX3**									
AIFM2 [M,LD]	1		7-27	VESGALHVVIVGGGFGGIAAA	373				
COLEC12	1	II	38-58	FSIILLYILCALLTITVAILG	742	4	1		
CYBRD1	6	II	12-32	LLGSALLVGFLSVIFALVWVL	286	1			
MAN1A1	1	II	42-62	FVLLLVFSAFITLCFGAIFFL	653	1	1		
ERMP1	9	II	64-84	AGTGLSEVRAALGLALYLIAL	904	2	2		NAT
DHRS7B	1	II	18-38	FITSTAILPLLFGCLGVFGLF	325		1		
ENPP1	1	II	77-97	VLSLVLSVCVLTTILGCIFGL	925	9	1		
ABCD3 [P]	4	II	84-104	GYLVLIAVMLVSRTYCDVWMI	659	3	1		
TMUB2	3	III	36-56	VMVVAGVVVLILALVLAWLST	321				
SGCD	1	II	37-57	FFVLLLMILILVNLAMTIWIL	256	3	1		
PXMP2 [P]	4	II	31-51	LYPVLTKAATSGILSALGNFL	195				
TMEM192	4	II	47-67	TVIIVNLLWFIHLVFVVLAFL	271				
TMEM237	4	II	227-247	MIGLFSHGFLAGCAVWNIVVI	408				
PEX13 [P]	1		227-251	AATSAKSWPIFLFFAVILGGPYLIW	403				
TOR1AIP1	1		339-355	WLLPLIAALASGSFWFF	583				
ATL1	1	HP	450-470	TLFVVIFITYVIAGVTGFIGL	558				
RTN3	2	HP	864-887	LIMLLSLAAFSVISVVSYLILALL	1032				
VAMP3	1	TA	78-98	MWAIGITVLVIFIIIIIVWVV	100			ΔM	NAT
FAR1 [P]	1	TA	466-483	IRYGFNTILVILIWRIFI	515				
STX6	1	TA	235-255	WCAIAILFAVLLVVLILFLVL	255			ΔM	NAT
ACBD5 [P]	1	TA	497-517	GVLTFAIIWPFIAQWLVYLYY	525				

RHOT1 [M]	1	TA	593-615	WLRASFGATVFAVLGFAMYKALL	618
CCDC136	1	TA	1130-1150	IFSLPLVGLVVISALLWCWWA	1154

23

Component (in **bold** face); Protein, client protein; TMDs, number of transmembrane domains in client (including TMH); Type, membrane protein type; TA, tail anchor; TMH, most N-terminal transmembrane domain; TMH Sequence, primary structure of TMH; Size, number of amino acid residues of client; NG, number of N-glycosylations; LD, number of ER lumenal domains with a content of > 50 amino acid residues; ΔM, N-terminal methionine excision; NAT, N-terminal acetylation. MP types shown in red as well as LDs of multispanning MPs were determined by employing the most advanced prediction tool for this purpose (https://dtu.biolib.com/DeepTMHMM/). Clients were screened for N-terminal methionine excision and N-acetylation in the NCBI protein database (https://www.ncbi.nlm.nih.gov/protein).

* highlights peroxisomal membrane proteins among the SR, Wrb and PEX3 clients;
§ highlights overlap with Snd2 and/or Wrb clients;
# highlights overlap with Sec61 clients; $ highlights overlap with SR clients;
+ highlights overlap with TRAP clients.

Notably, Wrb and Snd2, respectively, refers to the pool of clients that were detected after the respective single depletion (Snd2 or Wrb) plus the double depletion (Snd2+Wrb), and PEX3 refers to the pool of clients that were detected after the PEX3 depletion in HeLa cells and in the Zellweger patient fibroblasts with a PEX3 deficiency. The Table was updated for putative membrane protein clients that have their functional location in lipid droplets, peroxisomes, or mitochondria.
