# Supplementary material for: Rules of Engagement for Components of Membrane Protein Biogenesis at the Human Endoplasmic Reticulum
Source: Int J Mol Sci. 2025 Sep 10;26(18):8823. doi: 10.3390/ijms26188823 (PMC12469465; doi:10.3390/ijms26188823)
Supplement: Supplementary file 1 [file ijms-26-08823-s001.zip › supplementary files/IJMS_Table S7.pdf]

Table S7. Insertase clients as determined by MS and differential protein abundance analysis.

Clients of	Clients with			Clients with				
	SP	SP/ss	SP/ms	TMH	TMH/ss	TMH/ms	HP	TA
Sec61	***197***	*68*	*9*	***89***	***44***	***40***	**-**	***5***
Total	*286*							
% of total	*68.9 SP*			*31.1 TMH*				
% of total	*58.0 MP*							
% of TMH					*49.4*	*44.8*	-	*5.7*
% of total	*42.2sol*	*23.8*	*3.1*		*15.4*	*14.0*	-	*1.7*
% of MP		*41.0*	*5.4*	*54*	*26.5*	*24.1*	-	*3.1*
Clients with N-terminal topogenic sequences in % of MP					*86*			
Clients with N-terminal topogenic sequences in % of total					*92*			
TRAP	***67***	*15*	*7*	***57***	***13***	***42***	**-**	***2***
Total	*124*							
% of total	*54.0 SP*			*46.0 TMH*				
% of total	*63.7 MP*							
% of TMH					*22.8*	*73.7*	-	*3.5*
% of total	*36.3sol*	*12.1*	*5.6*		*10.5*	*33.9*	-	*1.6*
% of MP		*19.0*	*8.9*	*72*	*16.5*	*53.2*	-	*2.5*
Clients with N-terminal topogenic sequences in % of MP					*76*			
Clients with N-terminal topogenic sequences in % of total					*85*			
TRAM1	***13***	*2*	*2*	***17***	***10***	***5***	**-**	***2***
Total	*30*							
% of total	*43.3 SP*			*56.7 TMH*				
% of total	*70.0 MP*							
% of TMH					*58.8*	*29.4*	-	*11.8*
% of total	*30.0sol*	*6.7*	*6.7*		*33.3*	*16.7*	-	*6.7*
% of MP		*9.5*	*9.5*	*81*	*47.6*	*23.8*	-	*9.5*
Clients with N-terminal topogenic sequences in % of MP					*76*			
Clients with N-terminal topogenic sequences in % of total					*83*			
Sec62	***74***	*17*	*4*	***28***	***12***	***15***	**-**	***1***
Total	*102*							
% of total	*72.5 SP*			*27.5 TMH*				
% of total	*48.1 MP*							
% of TMH					*42.9*	*53.6*	-	*2.9*
% of total	*52.0sol*	*16.7*	*3.9*		*11.8*	*14.7*	-	*1.0*
% of MP		*34.7*	*8.2*	*57*	*24.5*	*30.6*	-	*2.1*
Clients with N-terminal topogenic sequences in % of MP					*84*			
Clients with N-terminal topogenic sequences in % of total					*92*			

Sec63	***32***	*11*	*3*	***35***	***13***	***21***	**-**	***1***
Total	*67*							
% of total	*47.8 SP*			*52.2 TMH*				
% of total	*73.1 MP*							
% of TMH					*37.1*	*60.0*	-	*2.9*
% of total	*26.9sol*	*16.4*	*4.5*		*19.4*	*32.3*	-	*1.5*
% of MP		*22.4*	*6.1*	*71*	*26.5*	*42.9*	-	*2.0*
Clients with N-terminal topogenic sequences in % of MP					*78*			
Clients with N-terminal topogenic sequences in % of total					*84*			
EMC	***11***	**-**	*2*	***48***	***1***	***45***	**-**	***2***
Total	*59*							
% of total	*18.6 SP*			*81.3 TMH*				
% of total	*84.7 MP*							
% of TMH					*2.1*	*93.8*	-	*4.2*
% of total	*15.3sol*	-	*3.4*		*1.7*	*76.3*	-	*3.4*
% of MP		-	*4.0*	*96*	*2.0*	*90.0*	-	*4.0*
Clients with N-terminal topogenic sequences in % of MP					*74*			
Clients with N-terminal topogenic sequences in % of total					*78*			

HP, hairpin proteins; MP, membrane proteins; ms, multispanning membrane proteins; sol, soluble proteins; SP, signal peptide-containing proteins; ss, single-spanning membrane proteins; TA, tail anchor proteins; TMH, membrane proteins with transmembrane helix that serves as SP-equivalent. Notably, TRAP clients refer to the pool of clients that were detected after TRAP depletion in HeLa cells plus in the CDG patient fibroblasts with a TRAP deficiency, due to either TRAPγ or TRAPδ deficiency; Sec62 and Sec63 clients, respectively, refer to the pool of clients that were detected after knock down in HeLa cells plus knock out in HEK293 cells. The Table was updated for putative membrane protein clients that have their functional location in lipid droplets, peroxisomes, or mitochondria.
