# Supplementary material for: Rules of Engagement for Components of Membrane Protein Biogenesis at the Human Endoplasmic Reticulum
Source: Int J Mol Sci. 2025 Sep 10;26(18):8823. doi: 10.3390/ijms26188823 (PMC12469465; doi:10.3390/ijms26188823)
Supplement: Supplementary file 1 [file ijms-26-08823-s001.zip › supplementary files/IJMS_Table S8.pdf]

Table S8. Characteristics of THM-containing clients of membrane insertion components Sec61, TRAP, TRAM1, Sec62, Sec63 and EMC.

Protein	TMDs	Type	TMH	TMH Sequence	Size	NG	LD	ΔM	NAT
**Sec61**									
ERAP1	1	II	2-21	VFLPLKWSLATMSFLLSSLL	948	5	1		
ANPEP	1	II	9-31	KSLGILGILLGVAAVCTIIALSVV	967	10	1		
SLC20A2	12	III	6-26	YLWMVILGFIIAFILAFSVGA	652	1			
CNNM3	4	II	11-27	LGWLFAALCLGNAAGEA	707	1	1		
ABCC3	16	III	33-53	TENPDLTPCFQNSLLAWVPCI	1527	2			
GALNT2	1	II	7-24	MLLCFAFLWVLGIAYYMY	571	1	1		
GOLIM4	1	II	13-33	IFQTLLLLTVVFGFLYGAMLY	696	1	1		
GALNT1	1	II	9-28	VVLATSLIWVLLDMFLLLYF	559	2	1		
NCEH1	1	II	5-25	CVLLTALVALAAYYVYIPLPG	408	3	1		
KIAA0319L	2	II	30-50	SLYLFYTCFCFSVLWLSTDAS	1049	5	1		
CLPTM1L	6	II	11-31	LVVGVFVVYVVHTCWVMYGIV	538	3	1		
MGAT1	1	II	7-29	AGLVLWGAILFVAWNALLLLFFW	445		1		
SLC30A1	6	II	11-31	LLCMLALTFMFMVLEVVVSRV	507	1			
ERLIN2	1	II	4-24	LGAVVAVASSFFCASLFSAVH	339	1	1		
SCARB1	2	II	12-32	GALGVAGLLCAVLGAVMIVMV	509	6	1		
MGAT2	1	II	10-29	VLILTLVVAACGFVLWSSNG	447	2	1		
SLC2A1	12	II	12-33	LMLAVGGAVLGSLQFGYNTGVI	492	1			NAT
SLC39A9	8	III	4-24	FISISLLSLAMLVGCYVAGII	307	2			
PIGG	12	II	432-452	IYSMMVGTVVVLEVLTLLLLSV	983	1			
ERLIN1	1	III	8-28	VLVAAVVGLVAVLLYASIHKI	348		1		
B3GAT3	1	II	8-28	VFLAYFLVSIAGLLYALVQLG	335	1	1		
HS2ST1	1	II	12-28	LQLLAVVAFAVAMLFLE	356		1		
CASC4	1	II	15-35	SLVLVVLLVVIVVLAFNYWSI	436		1		NAT
MOGS	1	II	39-59	STAGGVALAVVVLSLALGMSG	837	1	1		
SLC16A3	12		18-38	GGWGWAVLFGCFVITGFSYAF	465				
GOLM1	1	II	13-35	SPPLVLAALVACIIVLGFNYWIA	401	3	1		NAT
IMPAD1	1	II	13-33	VAVFCLLGLGVLYHLYSGFLA	359	1	1		NAT
ATP13A1[#]	10	II	67-87	VLPFAGLLYPAWLGAAAAGCW	1204	2	1	ΔM	NAT
ATP11C	10	II	67-85	FRRIANFYFLIIFLVQVTV	1132		2		
NEU1	1		19-42	ILGFWGGCRVWVFAAIFLLLSLAA	415	3			
SLC7A2	14	II	38-59	DLIALGVGSTLGAGVYVLAGEV	658	3			
ERMP1	9	II	64-84	AGTGLSEVRAALGLALYLIAL	904	2	2		NAT
ASPH	1	II	54-74	FFTWFMVIALLGVWTSVAVVW	758	2	1		
SLC25A40	6	II	19-39	MLASCTGAILTSVIVTPLDVV	338				
B4GALT1	1	II	25-44	LLVAVCALHLGVTLVYYLAG	398	1	1		
TMEM38B	7	III	20-33	FFDIAHYLVSVMAV	291				NAT
ERGIC3	2	II	26-46	GGATVTIVSGLLMLLLFLSEL	383	1	1		
GGCX	5	II	61-81	PASLAVFRFLFGFLMVLDIPQ	758	2	2	ΔM	NAT
CLN5	1	II	24-44	ASWCWALALLWLAVVPGWSRV	358	8	1		
PLD3	1	II	39-59	VLLVLILAVVGFGALMTQLFL	490	2	1		
LYRIC	1	III	49-69	GWVILVGTGALGLLLLFLLGY	582				
TFRC	1	II	68-88	YGTIAVIVFFLIGFMIGYLGY	760	4	1		
ECE1	1	II	69-89	LVVLVVLLAAGLVACLAALGI	770	9	1		
TMEM43	4	II	32-52	TSGGMFVGLMAFLLSFYLIFT	400			ΔM	NAT
SLC12A2	13	II	287-313	ESKGVVKFGWIKGVLVRCMLNIWGVM	1212		1		NAT
ITFG3	1	II	50-70	AAFFLSLFLCLFVVFVVSFVI	552	4	1		
ERGIC2	2	II	34-54	GTVSLIAFTTMALLTIMEFSV	377		1		
LNPEP	1	II	111-131	MVVCAFVIVVAVSVIMVIYLLPRCT	1025	17	1		NAT
SLC12A4	10	II	119-139	GTLMGVYLPCLQNIFGVILFL	1085	4	2		
SPCS3	1	II	12-32	FAFSLSVMAALTFGCFITTAF	180	1	1		
GPRC5A	7	III	34-54	AEAWGIVLETVATAGVVTSVA	357	1			
P4HTM	1	II	61-81	FLVLMVFVHLYLGNVLALLLF	563	3	1		
ERGIC1	1	II	27-47	IISICCCLFILFLFLSELTGF	290	1	1		
CANT1	1	II	45-62	VILTFFVGAAILWLLCSH	401	1	1		
MAN1B1	1	II	85-105	MILFLLAFLLFCGLLFYINLA	699		1		
GPX8	1		18-40	VFAVLLSIVLCTVTLFLLQLKFL	209				NAT
CXCR4	7	III	39-63	IFLPTIYSIIFLTGIVGNGLVILVM	352	3			
IKBIP	1	II	46-62	CLSLLSLGTCLGLAWFV	377	2	1		
CD70	1	II	18-38	VLRAALVPLVAGLVICLVVCI	193	2	1		
SEC11A	1	II	17-36	QLYYQVLNFGMIVSSALMIW	185		1		
VKORC1L1	4		17-37	VARYAVCAAGILLSIYAYHVE	176				

TMEM30A	2		50-70	VLPIFFIIGLIFIPIGIGIFV	361	4	1	ΔM	NAT
TMBIM6[#]	6	II	30-50	VYASFALCMFVAAAGAYVHMV	237				
BST2	1	II	21-48	KLLLGIGILVLLIIVILGVPLIIFTIKA	180	2	1		
CKAP4	1	II	107-127	ALNFLFYLALVAAAAFSGWC	602		1		
ABHD12	1	II	75-95	ILFCVLGLYIAIPFLIKLCPGI	398	1	1		
SCD	4	II	73-93	WRNIILMSLLHLGALYGITLI	359				
HTRA2	1	II	105-125	AWLAVALGAGGAVLLLLWGGG	458		1		
SUN2	1	II	213-233	FLWFLLPLLLLTCLTYGAWYF	717	1	1		
TOR4A	1	II	122-138	CLLLLVAIVGFQVLNAIENLDDNAQRY	423		1		
FADS2	4	II	132-152	FFLLLLAHIIALESIAWFTVF	444				
ANO6[#]	10	II	301-321	WLGYYTQMLLLAAVVGVACFL	910	6	2		
SFXN1	5	II	103-120	ITGCMMTFYRTTPAVLFW	322			ΔM	NAT
TOR1AIP1	1		339-355	LLPLIAALASGSFWFF	583	1			
SPCS2	2	II	87-107	ICTISCFFAIVALIWDYMHPF	226			ΔM	NAT
STEAP3	6	II	208-228	LLPAWKVPTLLALGLFVCFYA	488	2			
TOR1AIP2	1		215-235	FWSYGPVILVVLVVAVVASSV	470	2		ΔM	NAT
YIF1A	5	II	139-159	LYIPTMAFITYVLLAGMALGI	293			ΔM	NAT
LEMD3	2	II	475-495	MFLLTAACLFFLILGLTYLGM	911		1	ΔM	NAT
CLPTM1	5	II	355-375	YLLALTIIVSIVHSVFEFLAF	669	1	1	ΔM	NAT
DNAJB12	1	II	244-264	LGVFVQLMPILILILVSALSQ	375		1		NAT
ITPR3	6	II	2203-2223	LWGSISFNLAVFINIIIAFFY	2671		1		
FAR1	1	TA	466-483	IRYGFNTILVILIWRIF	515				
STX6	1	TA	235-255	VSHMTSDRRQWCAIAILFAVLLVVLILFLVL	255			ΔM	NAT
MOSPD2	1	TA	497-518	LLLSLTMLLLAFVTSFFYLLYS	518				
CDKAL1	1	TA	556-578	DCALRMSVGLALLGLLFAFFVKVY	579				
FNDC3B	1	TA	1182-1202	IIVLGFATLSILFAFILQYFLM	1204				

87

TRAP

SLC38A10	10	II	4-24	AAASNWGLITNIVNSIVGVSV	1119				
LMBRD2	9	III	6-21	LGLEIVFVFFLALFLL	695	1			
FAM20B	1	II	7-25	VVLLAILLVIFIFTKVFLI	409	1	1		
SLC2A1	12	II	12-33	LMLAVGGAVLGSLQFGYNTGVI	492	1			NAT
TPST2	1	II	9-25	LLAAGCALVLVLAVQLG	377	2	1		
ARMC10	1		5-27	RGAGWVAAGLLLGAGACYCIYRL	343				
SLC2A10	12	II	16-36	LLGGLTFGYELAVISGALLPL	541	1	1		
WLS	7	II	16-36	IVGGILLVFQIIAFLVGGLIA	541		1		
GLT8D1	1	II	8-28	IIILVLAVALFLLVLHHNFLS	371	2	1		
EXT2	1	II	26-46	YITLFSIVLLGLIATGMFQFW	718	2	1		
C1GALT1	1		7-29	LNFLTFLCGSAIGFLLCSQLFSI	363				
ARMCX6	1		7-27	VGWMAAGLMIGAGACYCVYKL	300				
ATP2A3	10	II	49-69	LWELVLEQFEDLLVRILLLAA	999				NAT
SLC44A2[+]	10	II	34-54	IICCVFLLLAIVGYVAVGIIA	706	3	2		
SLC39A11	7	III	12-32	LLGTFFTWGMTAAGAALVFVF	342				
NEU1	1		19-42	ILGFWGGCRVWVFAAIFLLLSLAA	415	3			
CHRM2	7	III	23-45	VVFIVLVAGSLSLVTIIGNILVM	466				
CDIPT	5	II	6-26	IFLFVPNLIGYARIVFAIISF	213				
MFSD10	11	II	27-47	VVFLGLLLDLLAFTLLLPLLP	455				
SLC22A18	10	II	26-46	LLTYVLAATELTCLFMQFSI	424				
SLC7A14	15	II	58-78	ISLGVGSCVGTGMYVVSGLV	771	2			
STT3B	11	II	66-85	AGWQSLLSFTILFLAWLAGF	826	5	2	ΔM	NAT
CD63	4	II	12-32	LLYVLLLAFCACAVGLIAVG	238	3	1		
ATP1A2	10	II	86-106	EWVKFCRQLFGGFSILLWIGA	1020				
LPCAT4	2	II	40-62	CLLGALLAPIRVLLAFIVLFLLW	524	1			
TRAM1	8	II	30-50	VAMVFLLGLMFEITAKASIIF	374	1			
HM13	9	III	32-52	IALAYGSLLLMALLPIFFGAL	377	2			
LNPEP	1	II	111-131	MVVCAFVIVVAVSVIMVIYLLPRCT	1025	17	1		
CHST14	1		40-60	LLPSMLMFAVIVASSGLLLMI	376	2			
TMEM205	4	III	18-38	VLSGAWGMQMWVTFVSGFLLF	189				
DEGS1	6	II	41-61	PNLIWIIMMVLTQLGAFYIV	323				
TMEM39A	8	II	72-92	SLLFEFLFFIYLLVALFIQYI	488	1			
TMBIM6[+]	6	II	30-50	VYASFALCMFVAAAGAYVHMV	237				
ATP13A3[+]	11	II	206-226	LLIKEVLNPFYIFQLFSVILW	1226				
SGPP1	9	II	133-152	CFGTELGNELFYILFFPFWI	441				
TMEM189	3	II	48-68	WCSVILCFSLIAHNLVHLLLL	271		1		
KCNJ2	2	II	82-106	RWMLVIFCLAFVLSWLFFGCVFWLI	427				

SGMS2	6	II	80-100	IAFIYAVFNLVLTTVMITVV	365				
TSPAN10	3	II	79-99	IFLSNFPFSLLGLLALAIGL	355	1	1		
TMEM223	2	II	44-64	FTILGLFCAGQGVFWASMAV	202				
SOAT1 +	9	II	139-160	RTIYHMFIALLILFILSTLVVD	550				NAT
TMEM166	1		35-55	ALYFVSGVCIGLVLTLAALVI	152				
TMEM159	2	II	44-61	YLDSHPFLAFTLLVFIVM	161	1			
ANO10	8	II	208-228	IALYFGFLEYFTFALIPMAVI	660				
DAD1	3	II	31-51	LLYILLTGALQFGYCLLVGTF	113				NAT
SPCS2	2	II	87-107	ICTISCFFAIVALIWDYMHPF	226			ΔM	NAT
PIGG	12	II	432-452	IYSMMVGTVVVLEVLTLLLLSV	983	1			
TMEM261	2	II	52-69	VLSGLGLMGAGGYVYWVA	116				
SLC4A2 +	10	II	708-731	CLAAVIFIYFAALSPAITFGGLLG	1241	3			
PEX13	1		227-251	AATSAKSWPIFLFFAVILGGPYLIW	403				
NOC4L	3		297-317	ACDLGGALSLLALNGLFILI	516				
TMEM199 +	2	II	146-166	LVITIFNFIVTVVAAFVCTYL	208			ΔM	NAT
STX17	2		229-249	LAALPVAGALIGGMVGGPIGL	302			ΔM	NAT
TMEM55B	9	II	212-232	ICCFLLGLLLAVTATGLAFG	277				
WDR11	1		1127-1147	ALLVLLSLGCFFSVAETLHSM	1224		1		
MOSPD2	1	TA	497-518	LLLSLTMLLLAFVTSFFYLLYS	518				
JPH2	1	TA	675-695	ILICMVILLNIGLAILFVHLL	696				
57									
**TRAM1**									
GALNT3	1	II	20-37	FWKLGAVIFFFIIVLVLM	633	3	1		
ERLIN2	1	II	4-24	LGAVVAVASSFFCASLFSAVH	339	1	1		
NEU1	1		19-42	ILGFWGGCRVWVFAAIFLLLSLAA	415	3			
KCNN4	6	II	29-49	LVLAGTGIGLMVLHAEMLWF	427				
CLN5	1	II	24-44	ASWCWALALLWLAVVPGWSRV	358	8	1		
PLD3	1	II	39-59	VLLVLILAVVGFGALMTQLFL	490	2	1		
LYRIC	1	III	49-69	GWVILVGTGALGLLLLFLLGY	582				
LNPEP	1	II	111-131	MVVCAFVIVVAVSVIMVIYLLPRCT	1025	17	1		NAT
P4HTM	1	II	61-81	FLVLMVFVHLYLGNVLALLLF	563	3	1		
SEC11A	1	II	17-36	QLYYQVLNFGMIVSSALMIW	185		1		
DEGS1	6	II	41-61	PNLIWIIIMMVLTQLGAFYIV	323				
PLP2	4	II	25-45	GILLFAEIILCLVILICFSAS	152	2			
TMEM223	2	II	44-64	FTILGLFCAGQGVFWASMAV	202				
TOR4A	1		122-138	CLLLLVAIVGFQVLNAIENLDDNAQRY	423		1		
STEAP3	6	II	208-228	LLPAWKVPTLLALGLFVCFYA	488	2			
BNIP3L	1	TA	188-208	VFIPSLFLSHVLALGLGIYIG	219				
JPH1	1	TA	640-660	IMIVLVMLLNIGLAILFVHFL	661				
17									
**Sec62**									
PIEZO1	36	II	5-25	VLGAVLYWLLLPCALLAACLL	2521	2	2		
PIGN	15		2-24	LLFFTLGLLIHFVFFASIFDIYF	931	3	1		
PIGO	14		4-24	ASVLLFLAWVCFLFYAGIALF	1089	1	1		
ERAP1	1	II	2-21	VFLPLKWSLATMSFLLSSLL	948	5	1		
SLC20A2	12	III	6-26	YLWMVILGFIIAFILAFSVGA	652	1			
B3GALTL	1	II	7-27	WWLLAPPALLALLTCSLAFGL	498	1	1		
MBOAT7	7	III	6-22	WTYLVVLLISIPIGFLF	472	1			
FAM20B	1	II	7-25	VVLLAILLVIFIFTKVFLI	409	1	1		
ERLIN2	1	II	4-24	LGAVVAVASSFFCASLFSAVH	339	1	1		
LRRC8C	4	II	23-43	WWDVFTDYLSVAMLMIGVFGC	803	2	1		
AGPAT4	4	II	11-31	FLCHLVFCYVFIASGLIINTI	378		1		
MOGS	1	II	39-59	STAGGVALAVVVLSLALGMSG	837	1	1		
UXS1	1	II	20-40	LLLGIALLAYVASVWGNFVNM	425	1	1		NAT
SLC7A2	14	II	38-59	DLIALGVGSTLGAGVYVLAGEV	658	3			
POMK	1	II	21-43	VGLLLIMALMNTLLYLCLDHFFI	350	3	1		NAT
CLN5	1	II	24-44	ASWCWALALLWLAVVPGWSRV	358	8	1		
LNPEP	1	II	111-131	MVVCAFVIVVAVSVIMVIYLLPRCT	1025	17	1		NAT
P4HTM	1	II	61-81	FLVLMVFVHLYLGNVLALLLF	563	3	1		
BST2	1	II	21-48	KLLLGIGILVLLIIVILGVPLIIFTIKA	180	2	1		
VANGL2	4	II	109-129	VAAGATLALLSFLTPLAFLLL	521				
CNNM2	3		251-271	FLLPFWLQVIFISLLLCLSGM	875	1			
AGPAT3	2	II	125-145	ELLYVPLIGWTWYFLEIVFCK	376		1		
YIPF2	5	II	125-145	FWICATLAFVLAVTGNLTLVL	316			ΔM	NAT

TOR1AIP2	1		215-235	FWSYGPVILVVLVVAVVASSV	470	1	1	ΔM	NAT
ARL6IP6	3	II	111-131	ILCSLLFAILLAFLLAIAYLI	226				
RNF5	2	II	118-138	GGFHFSFGVGAFPFGFFTTVF	180			ΔM	NAT
ITPR3	6	II	2203-2223	LWGSISFNLAVFINIIAFFY	2671		1		
RHOT2 [M]	1	TA	593-615	GLLGVVGAAVAAVLSFSLYRVLV	618				

28

Sec63

TMTC3	9	II	9-29	ITLIVGVVTACYWNSLFCGFV	914	3	2		
SLC20A2	12	III	6-26	YLWMVILGFIIAFILAFSVGA	652	1			
GALNT13	1	II	5-27	VYCKVVLATSLMWVLVDVFLLLY	556	3	1		
FAM20B	1	II	7-25	VVLLAILLVIFIFTKVFLI	409	1	1		
STT3A	11	II	18-38	LKLLILSMAAVLSFSTRLFAV	705	3	1		
LRRC8C	4	II	23-43	WWDVFTDYLSVAMLMIGVFGC	803	2	1		
LRRC8E	4	II	23-43	WWDVLAEYLTVAMLMIGVFGC	796	2	1		
ILVBL	1		13-33	LFPSFLLLACGTLVAALLGAA	510				
MGAT2	1	II	10-29	VLILTLVVAACGFVLWSSNG	447	2	1		
GLT8D1	1		8-28	IIILVLAVALFLLVLHHNFLS	371	2			
AGPAT4	4	II	11-31	FLCHLVFCYVFIASGLIINTI	378		1		
DGKE	2		22-42	LILWTLCSVLLPVFITFWCSL	567				
UXS1	1	II	20-40	LLLGIALLAYVASVWGNFVNM	425	1	1		NAT
ATP2C1	10	II	71-91	LWKKYISQFKNPLIMLLLASA	919				
GGCX	5	II	61-81	PASLAVFRFLFGFLMVLDIPQ	758	2	2	ΔM	NAT
CLN5	1	II	24-44	ASWCWALALLWLAVVPGWSRV	358	8	1		
NIPA1	9	III	28-48	VSLGLGVAVVSSLVNGSTFVL	329				
ELOVL1	7	III	23-43	PLMGSPLLMTSILLTYVYFVL	279				NAT
APMAP	1	II	41-61	VTFLMLAVSLTVPLLGAMMLL	416	2	1	ΔM	NAT
MAN1B1	1	II	85-105	MILFLLAFLLFCGLLFYINLA	699		1		
IKBIP	1		46-62	CLSLLSLGTCLGLAWFV	377	2			
BST2	1	II	21-48	KLLLGIGILVLLIIVILGVPLIIFTIKA	180	2	1		
VANGL2	2	II	109-129	VAAGATLALLSFLTPLAFLLL	521				
ALG9	8	II	136-156	ILVFYFLRCLLAFVSCICELY	618	2	1		
TMEM181	9	II	153-173	HFVLVFVVFFICFGLTIFVGI	612				
UBIAD1	8	II	83-103	LLVGCAVAVLAVHGAGNLVNT	338			ΔM	NAT
CNNM2	3	II	251-271	FLLPFWLQVIFISLLLCLSGM	875	1			
MARVELD2	6	II	195-215	ILGVVELLLGAGVFACVTAYI	558				
YIPF2	5	II	125-145	FWICATLAFVLAVTGNLTLVL	316			ΔM	NAT
TEX2	2	II	475-495	TLGFFIMCVYVYLILPLPHYV	1127	1			
ARL6IP6	3	II	111-131	ILCSLLFAILLAFLLAIAYLI	226				
TBC1D9B	1		668-688	LSWFLTLFLSVMPFESAVVIV	1233				
UNC80	4		2268-2288	PFVLQLFASVAPLLEFPDAAN	3258				
TMEM199	2	II	146-166	LVITIFNFIVTVVAAFVCTYL	208			ΔM	NAT
PARP16	1	TA	288-308	SHWFTVMISLYLLLLLIVSVI	322				

35

EMC

PIEZO1	36	II	5-25	VLGAVLYWLLLPCALLAACLL	2521	2	2		
ALG10	12	II	7-27	YYFSAALSCTFLVSCLLFSAF	473				
LRRC8E	4	II	23-43	WWDVLAEYLTVAMLMIGVFGC	796	2	1		
GDE1	2		4-24	WEDQGGLLGPFSFLLLVLLLV	331	2	1		
SLC9A7	14		22-42	LLLLPLLLGWGLRVAAAASAS	726	1			
PLPP2	6	II	5-25	WVFVLLDVLCLLVASLPFAIL	288	1	1		
SLC43A3	12	II	17-37	LLECLGFAGVLFGWPSLVFVF	491	2			
SLC44A1	9	II	31-51	WLLLFILFCIGMGFICGFSIA	657		2		
SLC44A2 [+]	10	II	34-54	IICCVFLLLAIVGYVAVGIIA	706	2	1		
ATP11A	10	II	62-82	PKNLFEQFRRVANFYFLIIFL	1134				
ATP13A1 [$#]	10	II	67-87	VLPFAGLLYPAWLGAAAAGCW	1204	2	1	ΔM	NAT
BCAP31	3	III	7-27	AVATFLYAEVFVVLLLCIPFI	246				
BCAP29 [§]	3	III	7-27	AVATFLYAEIGLILIFCLPFI	241				
SLC7A1	14	II	36-57	DLVALGVGSTLGAGVYVLAGAV	629	2			
TMEM19	6	II	15-35	MITNIVILSLIICISLAFWII	336		1		
ABCC4	14	II	93-113	LVLGIFTLIEESAKVIQPIFL	1325	1	1		
TMBIM6 [#+]	6	II	30-50	VYASFALCMFVAAAGAYVHMV	237				
ZDHHC6	4	II	25-45	IIALGVIAICSTMAMIDSVLW	413				
ATP2B4	9	II	93-113	FLELVWEALQDVTLILEIAA	1170		1		
SGPL1	1	III	41-61	LIAWSVVWTLLIVWGYEFVF	568				

ATP2B1	10	II	106-126	LQDVTLIILEIAAIVSLGLS	1220			ΔM	NAT
ADCY9	12	II	118-138	YALFYIGFACLLWSIYFAVHM	1353	2	1		
SLC6A6	12	II	50-70	FVLSVAGGFVGLGNVWRFPYL	620	3			
TM4SF1	4	II	10-30	IGHSLVGLALLCIAANILLYF	202	2			
SLC1A3	8	II	48-68	NAFVLLTVTAVIVGTILGFTL	542				
TMEM97	4	II	10-30	VEWLLGLYFLSHIPITLFMDL	176				
SLC38A5	11	II	49-71	SFGMSVFNLSNAIMGSGILGLAY	472				NAT
DIRC2	11	II	52-72	WLVLLLFSLLAFVQGLVWNTW	478				
ATP6V0C	4	III	11-33	ASFFAVMGASAAMVFSALGAAYG	155				
CXCR4 [§]	7	III	39-63	IFLPTIYSIIFLTGIVGNGLVILVM	352	1			
MYADM	8	II	41-61	LLQLVSTCVAFSLVASVGAWT	322				
EBP	4	III	29-49	WHILAGLFSVTGVLVVTTWLL	230			ΔM	NAT
SLC38A1	10	II	75-97	MSVFNLSNAIMGSGILGLAFALA	487	1			
ATP13A3 [+]	10	II	206-226	LLIKEVLNPFYIFQLFSVILW	1226				
CLCN3	10	II	209-232	MNYIMYIFWALSFAFLAVSLVKVF	818	2			
SOAT1 [§+]	9	II	139-160	RTIYHMFIALLILFILSTLVVD	550				NAT
ANO6 [#]	10	II	301-321	WLGYYTQMLLLAAVVGVACFL	910	5	2		
YIPF3	5	II	149-169	IAGELYGPLMLVFTLVAILLH	350	1	1	ΔM	NAT
TCIRG1	8	II	386-404	VNPAPYTIITFPFLFAVMF	830		1		
ATP6V0A1	8	II	389-407	INPAPYTIITFPFLFAVM	837	1	1		
SLC4A2 [§+]	10	II	708-731	CLAAVIFIYFAALSPAITFGGLLG	1241	3			
ABCG2	6	II	396-416	IAQIIVTVVLGLVIGAIYFGL	655	1	1		
TMEM199 [+]	2	II	146-166	LVITIFNFIVTVVAAFVCTYL	208			ΔM	NAT
MOSPD1	2	II	159-179	SLLTVFLGVVCIAALMLPTLG	213				
ITPR1	6	II	2283-2335	FWSSISFNLAVLMNLLVAFFYPF	2758	1	1		
ZFPL1	1	TA	267-287	LLLLLGLLGFLALLALMSRLG	310				
FDFT1	1	TA	384-404	PIYLSFVMLLAALSWQYLTTL	417				
47									

Component (in **bold** face); Protein, client protein; TMDs, number of transmembrane domains in client (including TMH); Type, membrane protein type; TA, tail anchor; TMH, most N-terminal transmembrane domain; TMH Sequence, primary structure of TMH; Size, number of amino acid residues of client; NG, number of N-glycosylations; LD, number of ER lumenal domains with a content of > 50 amino acid residues; ΔM, N-terminal methionine excision; NAT, N-terminal acetylation. MP types shown in red as well as LDs of multispanning MPs were determined by employing the most advanced prediction tool for this purpose (https://dtu.biolib.com/DeepTMHMM/). Clients were screened for N-terminal methionine excision and N-acetylation in the NCBI protein database (https://www.ncbi.nlm.nih.gov/protein).

[#] highlights overlap between Sec61 and EMC clients;
[+] highlights overlap between TRAP and EMC clients;
[§] highlights overlap between EMC and WRB and SND.

Notably, TRAP clients refer to the pool of clients that were detected after TRAP depletion in HeLa cells plus in the CDG patient fibroblasts with a TRAP deficiency, due to either TRAPγ or TRAPδ deficiency; Sec62 and Sec63 clients, respectively, refer to the pool of clients that were detected after knock down in HeLa cells plus knock out in HEK293 cells. The Table was updated for putative membrane protein clients that have their functional location in lipid droplets, peroxisomes, or mitochondria.
