# Supplementary material for: Rules of Engagement for Components of Membrane Protein Biogenesis at the Human Endoplasmic Reticulum
Source: Int J Mol Sci. 2025 Sep 10;26(18):8823. doi: 10.3390/ijms26188823 (PMC12469465; doi:10.3390/ijms26188823)
Supplement: Supplementary file 1 [file ijms-26-08823-s001.zip › supplementary files/IJMS_Table S18.pdf]

Table S18. siRNAs for depletion of BiP or Snd2 and Wrb in HeLa cells.

**Name**	**Sequence**	**Source**	**Concentration (nM)**	**Time (h)**
HSPA5 siRNA	CCUUCGAUGUGUCUCUUCUtt	Qiagen	35	72
HSPA5-UTR siRNA	GCGGCUGUUUACUGCUUUUtt	Qiagen	35	72
**Name**	**Target sequence**	**Source**	**Concentration (nM)**	**Time (h)**
HSND2-UTR siRNA #2	CTCTATAGGGTCGTTGAATAA	Qiagen	20	96
HSND2 siRNA #3	AAGGGCAAAGTGGGCACGAGA	Qiagen	20	96
WRB-UTR siRNA #3	TGACACGTATGTACTAGTGAA	Qiagen	20	96
WRB siRNA #4	CACAGTCAACATGATGGACGA	Qiagen	20	96
