# Supplementary material for: Rules of Engagement for Components of Membrane Protein Biogenesis at the Human Endoplasmic Reticulum
Source: Int J Mol Sci. 2025 Sep 10;26(18):8823. doi: 10.3390/ijms26188823 (PMC12469465; doi:10.3390/ijms26188823)
Supplement: Supplementary file 1 [file ijms-26-08823-s001.zip › supplementary files/IJMS-3803115_Figure S9.pdf]

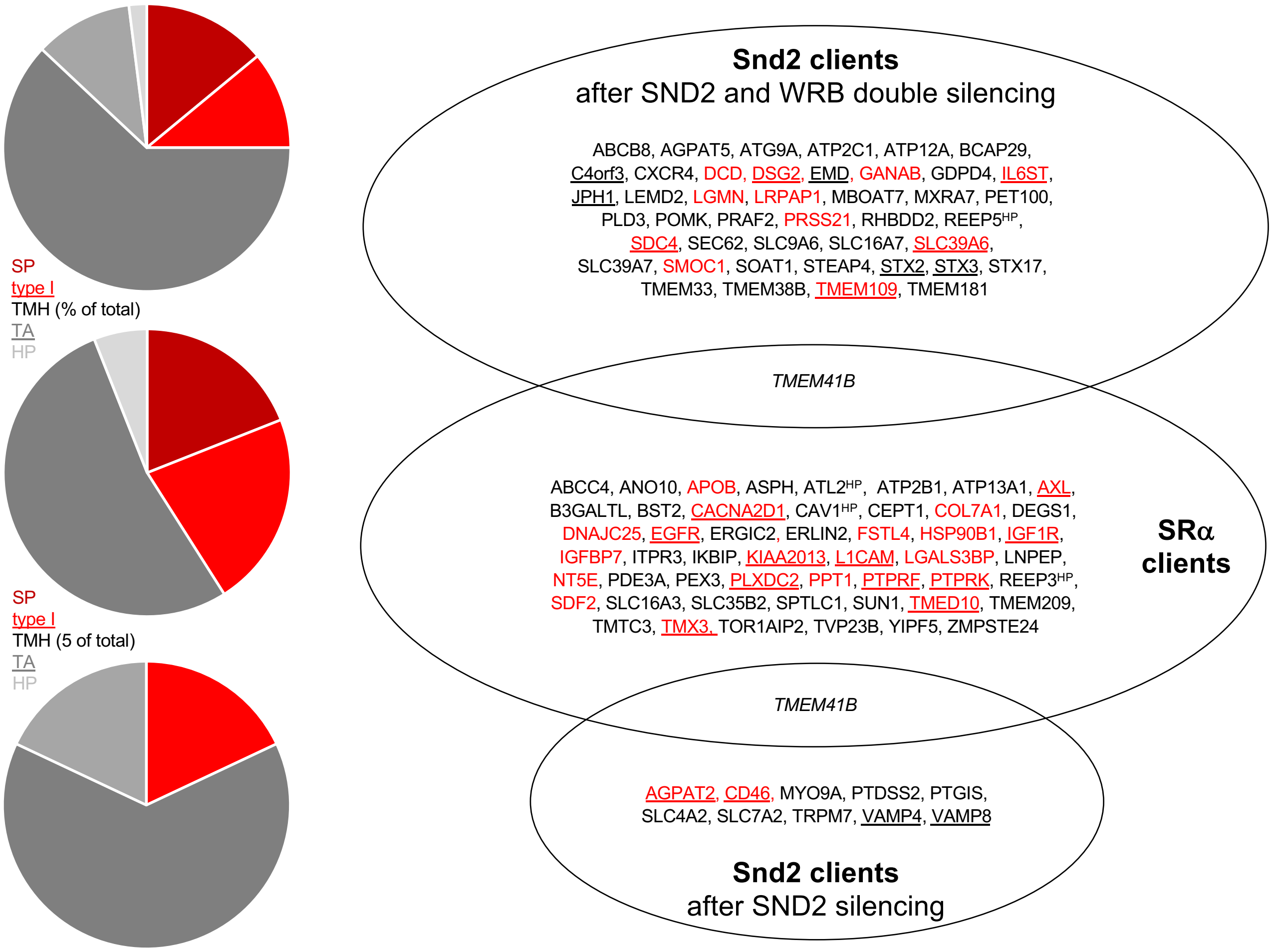


**Figure S9.** Pies and Venn diagrams for the clients of two components for protein targeting to the human ER: Snd2 and SRα. The clients were determined by quantitative MS and differential protein abundance analysis following depletion of the respective component(s) in HeLa cell for 96 h. Clients are defined as such by the presence of either an SP or at least one TMH. Pies: The client types are given in % of total proteins. Venn diagrams: Shown with their gene names, clients with SPs are shown in red, SP containing membrane proteins (type I MPs) are underlined, clients with TMH are shown in black and grey, TA proteins are underlined, hairpin proteins are indicated by superscript HP, and italics highlight clients of targeting components, which could not be properly fitted into the Venn diagram and are named twice. Notably, the upper pannels represents the Snd2 clients that were observed after SND2 plus WRB double silencing, the lower pannels represents the clients that were deteced after SND2 silencing. The data were previously reported by Tirincsi et al., 2022 [174].
