# Supplementary material for: Rules of Engagement for Components of Membrane Protein Biogenesis at the Human Endoplasmic Reticulum
Source: Int J Mol Sci. 2025 Sep 10;26(18):8823. doi: 10.3390/ijms26188823 (PMC12469465; doi:10.3390/ijms26188823)
Supplement: Supplementary file 1 [file ijms-26-08823-s001.zip › supplementary files/IJMS-3803115_Figure S8.pdf]

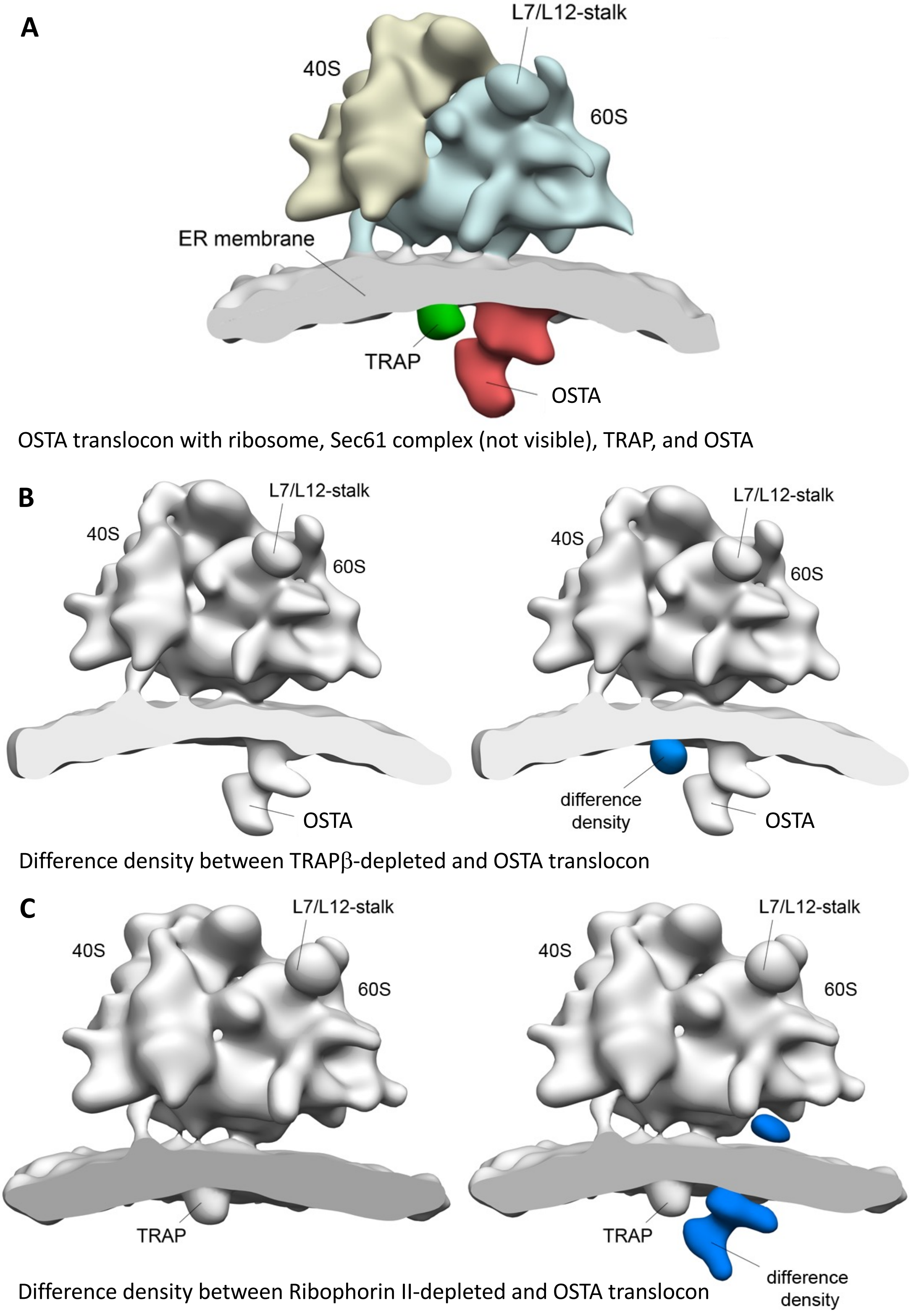

Figure S8. Localization of TRAP and OSTA in subtomogram averages of human ER membrane-associated ribosomes. (**A**) Subtomogram average for microsomes, isolated from HeLa cells treated with non-silencing control siRNA. (**B**) HeLa cells were treated with TRAPβ or control siRNA. The subtomogram average for microsomes, isolated from TRAPβ-depleted HeLa cells is shown with (right) and without (left) the difference density to the control (blue). (**C**) HeLa cells were treated with Ribophorin II or control siRNA. The subtomogram averages for microsomes, isolated from Ribophorin II-depleted HeLa cells are shown with (right) and without (left) the difference density to the control (blue). All structures were filtered to a resolution of 40 Å. The Figure and its legend were adapted from Pfeffer et al. [106].
