# Supplementary material for: Rules of Engagement for Components of Membrane Protein Biogenesis at the Human Endoplasmic Reticulum
Source: Int J Mol Sci. 2025 Sep 10;26(18):8823. doi: 10.3390/ijms26188823 (PMC12469465; doi:10.3390/ijms26188823)
Supplement: Supplementary file 1 [file ijms-26-08823-s001.zip › supplementary files/Legends to xlsx Tables S9-S20..pdf]

Legends to Tables 9 through 19.

Table S9_bip_all_summary

Alphabetical list of genes corresponding to proteins quantified in the BiP depletion experiments. Gene names, protein accession numbers, log2 fold changes resulting from siRNA-mediated BiP depletion, and log10 p values are indicated. Minus sign denotes negatively affected proteins. The number of listed proteins differs from the total number of quantified proteins because some proteins were quantified in less than two of the triplicates in at least one experiment. The original Orbitrap data for all quantified proteins are deposited at Proteome Exchange: http://www.proteomexchange.org

Table S10_bip_full_lo

Proteins that were negatively affected by BiP depletion, i.e. putative BiP clients. Gene names, protein accession numbers, and log2 fold changes resulting from siRNA-mediated BiP depletion are presented together with full protein names and Gene Ontology (GO) annotations for subcellular location(s), as extracted from UniProtKB entries using custom scripts. Proteins are listed according to decreasing negative effects of BiP depletion. BiP, product of the HSPA5 gene, is located at positions 144.

Table S11_new_bip_full_lo_2

Proteins that were negatively affected by siRNA-mediated BiP depletion and comprise signal peptides or transmembrane helices, which is serving as signal peptide, i.e. BiP clients. Gene names, protein accession numbers, and log2 fold changes resulting from BiP depletion are presented together with presence of signal peptide (SP) or transmembrane helix (TMH), total number of transmembrane helices, number of N-glycosylation sites (N-glc), amino acid sequences of SP or TMH (in single letter code), TMH position within the total amino acid sequence (all as extracted from UniProtKB entries using custom scripts) and glycine plus proline content of SP or TMH (GP%), hydrophobicity (Hph), predicted delta G (all as described in Methods). Proteins are listed according to decreasing negative effects of BiP depletion. BiP is not listed.

Table S12_bip_full_up

Proteins that were positively affected by BiP depletion. Gene names, protein accession numbers, and log2 fold changes resulting from siRNA-mediated BiP depletion are presented together with Gene Ontology (GO) annotations for subcellular location(s), as extracted from UniProtKB entries using custom scripts. Proteins are listed according to decreasing positive effects of BiP depletion.

Table S13_ws_all_summary

Alphabetical list of genes corresponding to proteins quantified in the hitherto unpublished hSnd2/Wrb double depletion experiment, termed WS in order to the previous double depletion that was termed SW []. Gene names, protein accession numbers, log2 fold changes resulting from siRNA-mediated depletion, and log10 p values are indicated. Minus sign denotes negatively affected proteins. The number of listed proteins differs from the total number of quantified proteins because some proteins were quantified in less than two of the triplicates in at least one experiment. The original Orbitrap data for all quantified proteins are deposited at Proteome Exchange: http://www.proteomexchange.org

Table S14_ws_full_lo

Proteins that were negatively affected by the hitherto unpublished hSnd2/Wrb double depletion, i.e. putative hSnd2 and Wrb clients. Gene names, protein accession numbers, and log2 fold changes resulting from siRNA-mediated depletion are presented together with full protein names and Gene Ontology (GO) annotations for subcellular location(s), as extracted from UniProtKB entries using custom scripts. Proteins are listed according to decreasing negative effects of depletion.

Table S15_ws_full_lo_2

Proteins that were negatively affected by the hitherto unpublished hSnd2/Wrb double depletion and comprise signal peptides or transmembrane helices, which is serving as signal peptide, i.e. hSnd2 and Wrb clients. Gene names, protein accession numbers, and log2 fold changes resulting from depletion are presented together with presence of signal peptide (SP) or transmembrane helix (TMH), total number of transmembrane helices, number of N-glycosylation sites (N-glc), amino acid sequences of SP or TMH (in single letter code), TMH position within the total amino acid sequence (all as extracted from UniProtKB entries using custom scripts) and glycine plus proline content of SP or TMH (GP%), hydrophobicity (Hph), predicted delta G (all as described in Methods). Proteins are listed according to decreasing negative effects of depletion.

Table S16_ws_full_up

Proteins that were positively affected by the hitherto unpublished hSnd2/Wrb double depletion. Gene names, protein accession numbers, and log2 fold changes resulting from siRNA-mediated depletion are presented together with Gene Ontology (GO) annotations for subcellular location(s), as extracted from UniProtKB entries using custom scripts. Proteins are listed according to decreasing positive effects of depletion.

Table S17_Summary of putative genetic interactions.

Table S18_ siRNAs for depletion of BiP or Snd2 and Wrb in HeLa cells.

HeLa cells (DSMZ no. ACC 57) were obtained from German Collection of Microorganisms and Cell Cultures and cultivated at 37°C in Dulbecco´s modified Eagle´s medium (DMEM; Gibco) containing 10% foetal bovine serum (FBS; Biochrom) and 1% penicillin/streptomycin (GE Healthcare) in a humidified environment with a 5% CO_2 atmosphere. Cell growth was monitored using the Countess® Automated Cell Counter (Invitrogen) according to the manufacturer´s instructions. The HeLa cells are replaced every five years and routinely tested for mycoplasma contamination by VenorGeM Mycoplasm Detection Kit (Biochrom AG, WVGM).

For most gene silencing experiments, 5.2×10^5 HeLa cells were seeded in a 6-cm culture plate in normal culture medium and then transfected with targeting siRNA (Table S18) or control siRNA (AllStars Negative Control siRNA, Qiagen) to a final concentration of 20 or 35 nM using HiPerFect Reagent (Qiagen) as described previously [49,92]. After 24 h, the medium was changed and the cells transfected a second time. After additional 24 or 48 h, silencing efficiencies were evaluated by Western blot analysis using the corresponding antibodies and a mouse anti-ß-actin antibody. The primary antibodies were visualized using goat anti-rabbit IgG-peroxidase conjugate and ECL™, ECL™ Plex goat anti-rabbit IgG-Cy5 or ECL™ Plex goat anti-mouse IgG-Cy3 conjugate, and the Fusion SL (peqlab) luminescence imaging system or the Typhoon-Trio imaging system in combination with Image Quant TL 7.0 software (GE Healthcare).

Table S19_Statistics for the identification of clients of targeting pathways to the ER.

Table S20_Statistics for the identification of clients of membrane insertion components.
